# A single probe for solvent dependent optical recognition of iron(II/III) and arsenite: discrimination between iron redox states with single crystal X-ray structure evidence

**DOI:** 10.1038/s41598-023-43154-2

**Published:** 2023-10-21

**Authors:** Jayanta Das, Milan Ghosh, Biplab Ghosh, Prasenjit Mandal, Sangita Maji, Debasis Das

**Affiliations:** Department of Chemistry, The University of Burdwan, Burdwan, West Bengal 713104 India

**Keywords:** Analytical chemistry, Fluorescent probes, Sensors

## Abstract

The detection and discrimination of Fe^2+^ and Fe^3+^ ions have been investigated using a simple probe (L), produced by the condensation of ethylenediamine and 3-ethoxysalicyaldehyde. Single crystal X-ray structures demonstrate that L interacts with Fe^2+^ and Fe^3+^. In aqueous-DMSO media, the L recognises AsO_2_^−^ by fluorescence and colorimetry techniques. The AsO_2_^−^ aided PET inhibition and H-bond assisted chelation enhanced fluorescence (CHEF) boost fluorescence by 91-fold. The L can detect 0.354 ppb Fe^2+^, 0.22 ppb Fe^3+^ and 0.235 ppt AsO_2_^−^.

## Introduction

Iron, the most widely distributed element plays key role in biology at different valence state^1–13^. In physiological systems, iron controls various cofactors in several proteins through redox shuttle, Fe^2+/3+^. It stimulates reactive oxygen species induced lipid peroxidation, DNA fragmentation to degrade the cellular contents and mitochondrial dysfunction. Its imbalance in homeostasis system leads various diseases and severe infections. Its lower (hypoferremia) or higher (hyperferremia) concentration may result Alzheimer, Parkinson’s and Huntington’s diseases^14–20^. Thus, detection, estimation and discrimination of Fe^2+^ and Fe^3+^ at trace level is really demanding in environmental and biological systems in order to decipher several correlated events.

On the other hand, arsenic, a carcinogen and teratogenic toxic species causes serious health problems like dermal toxicity, cardiovascular disease and neurodegenerative disorders^21,22^. Inorganic arsenic (arsenite and arsenate) are more abundant and toxic over organo arsenic species (monomethyl and dimethyl arsonic acid)^23^. Again, arsenite is more toxic than arsenate as it binds sulfhydryl unit of proteins intervening the reactions of other enzymes and proteins^24–30^.

Design of probes that convert molecular recognition into detectable signals is an attractive research area^1–7^. Moreover, bare eye (colorimetric) detection is preferable in biomedical analysis and environmental monitoring^10,11^, particularly in developing countries having poor infrastructure. In general, optical sensors that are simple, selective, sensitive, inexpensive and allow real-time monitoring of target analyte without requirement of any sophisticated instrument are still demanding^8,9^.

Literature suggests that most of the fluorescence sensors for iron suffer from paramagnetic quenching, and/interference from other common metal ions/high detection limit^31–43^. Available fluorescence sensors either can detect Fe^3+^ or Fe^2+^ following the protocol “fluorescence quenching amplification”, and most of them contain highly pH sensitive rhodamine moiety^44–48^. In addition, they can’t discriminate Fe^2+^ and Fe^3+^ ions. Neither, they could provide the most authenticate and desirable single crystal X-ray structure of the [probe-iron] complex/adduct.^9,49^.

So far literature is concerned, there are hardly any report on a single optical probe that can selectively detect as well as discriminate between Fe(II) and Fe(III) redox states in bare eye. The detection is highly sensitive and instantaneous in aqueous methanol. Most importantly, the binding event of the probe with both Fe^3+^ and Fe^2+^ have been established by single crystal X-ray structures of the resulting Fe(II/III) complexes.

On the other hand, there are few reports on arsenite selective TURN ON fluorescence probe having detection limit as low as 2 × 10^–12^ M. Having said that every probe has its own merits and demerits, very few reports deal with the use of the probe for solid phase extractive removal of arsenite from real samples^50,51^.

Interestingly, in present case, the L is capable for selective recognition of arsenite ion in aqueous DMSO (DMSO/H_2_O, 4/1. v/v)^52–57^.

Thus, novelty of our present report lies in the use of a single optical probe for detection of multiple analyte (Fe(II/ III), arsenite) in a solvent dependent manner. Moreover, the experimental findings have been substantiated by TDDFT studies.

## Experimental

### Materials and methods

High-purity buffer HEPES, 3-ethoxysalicyaldehyde and ethylenediamine (98%) have been procured from SigmaAldrich (India). FeSO_4_.7H_2_O, Mohr salt, FeCl_3_.6H_2_O has been procured from Merck (India). The spectroscopic grade solvents have been used. Other analytical reagent grade chemicals have been used without further purification unless otherwise specified. Milli-Q Millipore 18.2 MΩ cm^−1^ water is used whenever necessary. A Shimadzu Multi Spec 2450 spectrophotometer has been used for recording UV−vis spectra. FTIR spectra are recorded on a Shimadzu FTIR (model IR Prestige 21 CE) spectrophotometer. Mass spectra have been recorded using a QTOF 60 Micro YA 263 mass spectrometer in ES positive mode. Element analysis is carried out using Bruker ARTAX (serial number: 411280712). The steady state emission and excitation spectra are recorded with a Hitachi F-4500 spectrofluorimeter. A Systronics digital pH meter (model 335) is used for pH measurement. Time-resolved fluorescence lifetime measurements have been performed with a pico-second pulsed diode laser-based time-correlated single-photon counting (TCSPC) spectrometer (IBH, UK, λ_ex_ = 384 nm) coupled to MCP-PMT detector (model FL-1057). ^1^HNMR have been recorded on a Bruker Advance III HD (400 MHz) spectrometer. Chemical shifts values are reported in parts per million (ppm), and the residual solvent peak is used as an internal reference: tetramethylsilane (TMS, δ 0.00) is used as a reference. Multiplicity are indicated as follows: s (singlet), d (doublet), t (triplet), q (quartet), m (multiplet). Coupling constants are reported in Hertz (Hz). Data are fitted to multi-exponential functions after deconvolution of the instrument response function by an iterative reconvolution technique using IBH DAS 6.2 data analysis software. The single crystal X-ray diffractions have been performed on a Bruker X8 APEXIII CCD diffractometer, using graphite-monochromated Mo-Kα radiation (λ = 0.71073 Å). Data are processed and corrected for Lorentz and polarization effects. Structures are solved by the standard direct methods and refined by full matrix least squares on F2. All non-hydrogen atoms are anisotropically refined. Hydrogen atoms are included in the structure factor calculation in geometrically idealized positions, with thermal parameters depending on the parent atom, using a riding model. Cyclic voltammetry (CV) experiments of two complexes are recorded on a CHI620D potentiometer using the three electrode configuration: a platinum disk working electrode, a platinum wire auxiliary electrode and a calomel reference electrode. The electrochemical measurement is carried out in DMF solution, extensively purged with N_2_ prior to the measurements, using TBAP as supporting electrolyte. All potentials are determined at a scan rate of 100 mVs^−1^ at room temperature.

### Synthesis of L

**Scheme 1:**
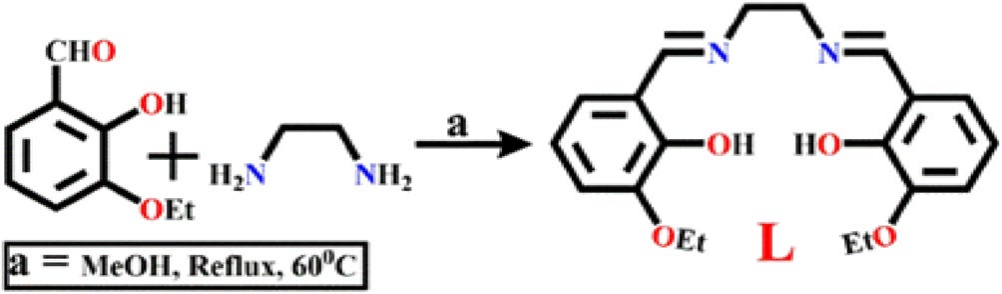
Synthetic protocol ((**a**): MeOH, reflux, 60°C).

The probe, L has been synthesized by refluxing the mixture of 3-ethoxysalicyaldehyde, (0.50 g, 3.01 mmol) and ethylenediamine (0.09 g, 1.50 mmol) in methanol for 6 h at 60 °C (Scheme 1). The yellow crystals obtained after slow evaporation of the solvent in 96% (1.03 g) yield. Single crystal of L, suitable for X-ray diffraction is analyzed at 296 K. It’s CCDC No. 1571936. Table S1 (ESI) listed the crystal data and refinement parameters of L. Anal. calcd (%): C, 67.40; H, 6.79 and N, 7.86; found: C, 67.01; H, 6.50 and N, 7.91. QTOF–MS ES^+^ (Fig. S1, ESI): m/z calcd. for C_20_H_24_N_2_O_4_, 356.17, found,357.25 ([M + H]^+^), 375.24 ([M + H_2_O + H]^+^). ^1^HNMR (Fig. S2a, ESI) (400 MHz, CDCl_3_), δ (ppm): 13.614 (1 H, s), 8.379 (1 H, s), 8.312 (1 H, s), 7.259 (1 H, s), 6.913–6.731 (4 H, m, J = 2.8), 4.116–3.937 (8 H, t, J = 6.8), 3.022 (solvent peak).^13^CNMR (Fig. S2b, ESI): 167.64, 152.03, 147.55, 123.72, 118.88, 118.29, 116.51, 64.33, 58.93, 40.59, 39.34 and 15.24. FTIR (cm^−1^) (Fig. S3, ESI): υ(O–H) 3707 (H– bonded), υ (C–H, aromatic) 2974, 2949, υ (CH = N, imine) 1678, υ (C=C, stretch) 1523, υ (C–N, stretch) 1382, υ (C–O, stretch) 1056. UV–Vis. (Fig. S4, ESI): λ (nm) in MeOH/H_2_O (4/1, v/v) (ε, M^−1^ cm^−1^), 298 nm (6.9 × 10^4^), 348 nm (5.93 × 10^5^). Excitation and emission spectra (Fig. S4, ESI): λ_ex_ = 322 nm and λ_em_ = 414 nm in MeOH/H_2_O (4/1, v/v).

### Synthesis of [L-Fe^2+^] complex

Methanol solution of Mohrs’ salt, (NH_4_)_2_Fe(SO_4_)_2_(H_2_O)_6_ (0.70 g, 1.78 mmol) is added drop-wise to a magnetically stirred solution of L (0.63 g, 1.78 mmol) in methanol in presence of sodium azide (0.50 g, 7.69 mmol) to maintain a reducing atmosphere of the reaction media at room temperature. Upon removal of solvent by slow evaporation, intense red crystals have been obtained. Single crystal has been subjected to diffraction using the instrument as mentioned *supra*. The crystal belongs to ‘P 21/c’ space group and received CCDC No. 1576234. The crystal data and refinement details are listed in Table S1 (ESI). Anal. calcd. (%): C, 47.40; H, 5.97 and N, 25.13; found: C, 47.36; H, 5.89 and N, 25.39. The QTOF–MS ES^+^ (Fig.S5, ESI), m/z calcd. for C_20_H_22_FeN_8_O_4_: 494.11, found: 495.16 ([M + H]^+^), 513.12 ([M + H_2_O + H]^+^). FTIR (cm^−1^) (Fig. S6, ESI): υ(C–H) 2976, 2968, 2888; υ (N=N, azide) 1797, υ(C–O) 1456, υ(C=C), 1244.

### Synthesis of [L-Fe^3+^] complex

Methanol solution of FeCl_3_.6H_2_O (0.50 g, 1.85 mmol) has been added dropwise to a magnetically stirred solution of L (0.69 g, 1.85 mmol) in methanol at room temperature. Slow evaporation of solvent yields brown crystals suitable for single crystals X-ray diffraction in 96% yield. The crystal belongs to P 21/n space group and received CCDC No. 1542015. The crystal data and refinement details are listed in Table S1 (ESI). Anal. calcd (%): C, 51.80; H, 5.22 and N, 6.04; found: C, 51.96; H, 5.20 and N, 5.96. The QTOF–MS ES^+^ (Fig. S7, ESI): m/z calcd. for C_20_H_24_FeClN_2_O_5_: 463.07, found: 464.01 ([M + H]^+^), 486.11 ([M + Na]^+^). FTIR (cm^−1^) (Fig. S8, ESI): υ(O–H) 3369, υ(C–H) 2980 and 2875, υ(C=N) 1627, υ(C=C) 1571, υ(C–N) 1328, υ (C–O, attached with carbonyl group) 1244, 1112.

### Synthesis of [L-AsO_2_^−^] adduct

The [L-AsO_2_^−^] adduct is isolated as a solid residue upon slow evaporation of the reaction mixture in methanol containing sodium arsenite (0.26 g, 1.99 mmol) and L (0.69 g, 1.99 mmol). Anal. calcd (%): C, 51.85; H, 5.22 and N, 6.05; found: C, 51.96; H, 5.20 and N, 5.96. QTOF–MS ES^+^ (Fig. S9, ESI):m/z calcd.: 463.19, found: 464.36 ([M + H]^+^), 486.40 ([M + Na]^+^). FTIR (cm^−1^) (Fig. S10, ESI): υ(O–H) 3321, υ(C-H) 2855, υ(C=N) 1622, υ(C=C) 1510, υ(C–N) 1444, υ(C–O, attached with carbonyl group) 1259, 1141.

## Results and discussion

### Crystal structure description

**Figure 1:**
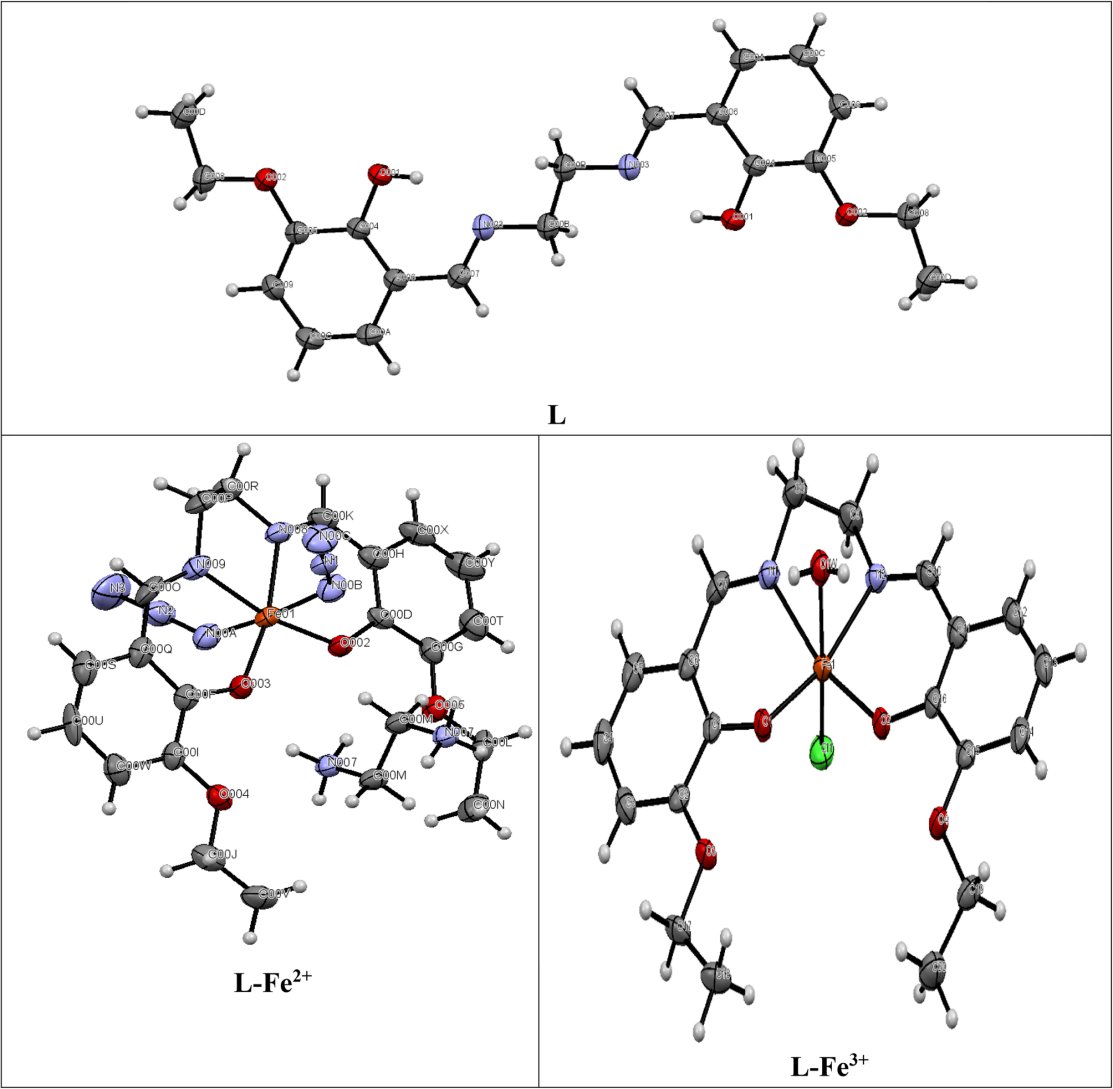
ORTEP view with 50% probability ellipsoid.

Figure 1 shows the ORTEP views of single crystal X-ray structures of L and its mononuclear Fe(II) and Fe(III) complexes. The crystal refinement parameters, bond lengths and angles are presented in Tables S1-S4 (ESI). The effective van der Waals charge distribution across multiple layers is depicted in Fig. S11 (ESI). Fig. S12 (ESI) provides a space-filled image of the L, Fe(II) and Fe(III) complexes. Both the intra and inter-molecular hydrogen bonding that are present in parallel layers have been presented in Fig. S13 (ESI). The crystal packing in wire frame mode of L, [L-Fe^2+^] and [L-Fe^3+^] complexes is portrayed in Fig. S14 (ESI). The 3D structure (polygon view) is shown in Fig. S15 (ESI), which has 1D open channels adorned by synchronized Fe centers.

The L belongs to triclinic crystal system having space group P -1'. It's three dimensional connectivity is illustrated in Fig. S11a (ESI) in terms of Van der Waals hypothesis regarding distribution of charge across distinct layers. A clear potential barrier connects two L(Fig. S13a, ESI). The nitrogen atom of the imine forms two intra-molecular hydrogen bonds involving hydrogen atoms on the -OH moiety. Figure S16 (ESI) shows the efficient 3D crystal packing of L, [L-Fe^2+^] and [L-Fe^3+^] in a wire frame model.

Figure 1 points out that the asymmetric unit of the [L-Fe^2+^] complex possesses deformed octahedral geometry. The Fe(II) center is surrounded by two imine nitrogen, two oxygen from the –OH moiety and two azide nitrogen aligned axially. Two imine nitrogen of L (N008, N009) and two oxygen (O002, O003) of –OH moieties adorn the equatorial positions, while two azide nitrogen (N00A, N00B) acquire the axial positions, in opposite directions. The selected bond distances and angles are presented in Table S3 (ESI). Furthermore, protonated ethylene diamine has also find its space in between alternate layers. Overall, hydrogen bond assisted powerful 3D- network, comprising of the complex and protonated ethylene diamine molecules have been observed. Moreover, substantial hydrogen bonding (N–H···O) interaction exists among oxygen centers of the neighbouring salicylaldehyde moieties of L as evident from the bond lengths (O003···H-N007, 1.978Å and N007-H···O002, 2.219Å) and bond angles (O–H···O, 154.590° and 133.250° respectively (Fig. S13, ESI). The 3D connections in the [L-Fe^2+^] complex in terms of van der Waals charge distribution in discrete layers are shown in Fig. S11b (ESI).

On the other hand, the Fe^3+^complex of L assumes distorted octahedral geometry where two phenol oxygen and two ethylenediamine nitrogens are coordinated (Figure 1). The oxygen (O2, O3) and imine nitrogen (N1, N2) in the complex, [Fe(L)(H_2_O)(Cl)] remain in equatorial plane while water and chloride are in axial positions. It's selected bond distance and angles are presented in Table S4 (ESI). Notably, the gap between the Fe(I) and Cl(I) centers is slightly larger than that of Fe(I) and O(1w). Intermolecular hydrogen bonding between an oxygen of the salicylaldehyde moiety and two O–H units of water is clearly visible in Fig. S13c (ESI).

### Spectroscopic studies

**Scheme 2:**
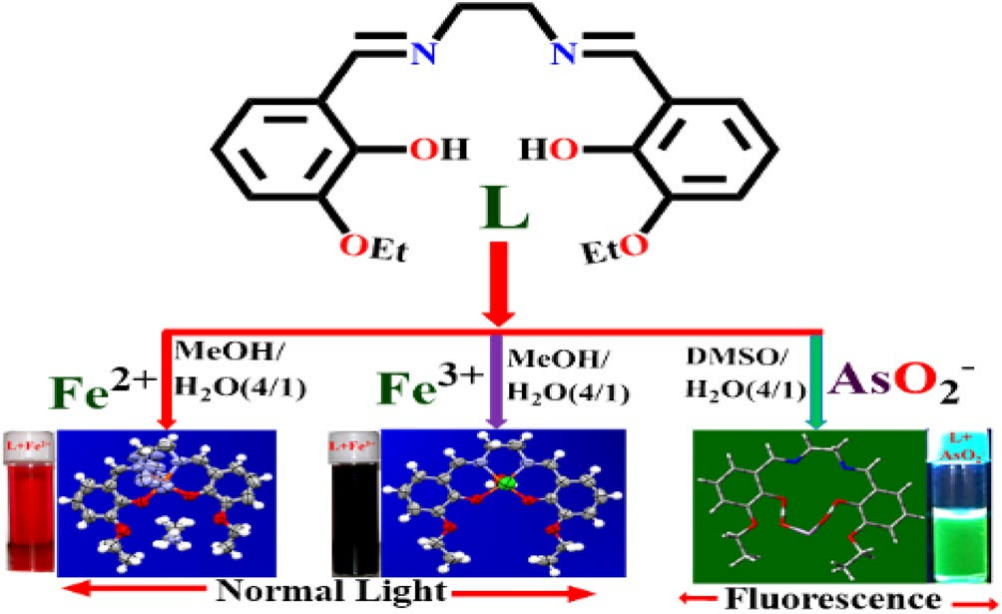
Solvent dependent instantaneous discrimination of Fe^2+^, Fe^3+^ and AsO_2_^−^ ions.

**Figure 2:**
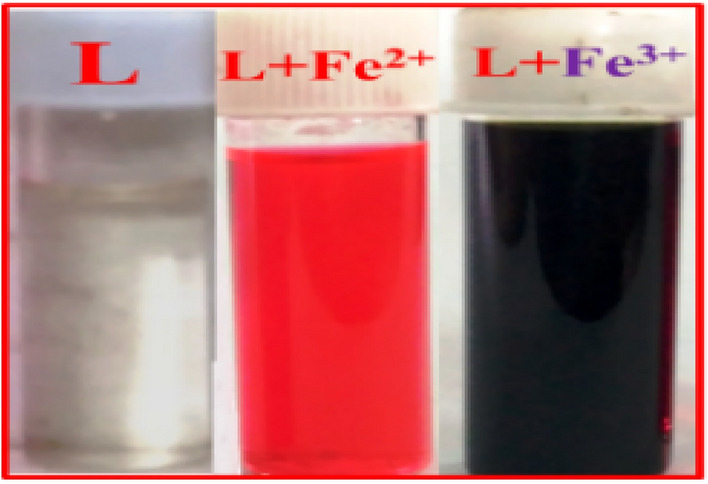
Bare eye view of L (20 µM) in presence and absence of Fe^2+^ and Fe^3+^ (MeOH/ H_2_O, 4/1, v/v, pH 7.4).

L is successfully synthesized in 96% yield and its physicochemical properties have been thoroughly characterized (Figs. S1–S4, ESI). Intense peaks at 298 nm (ε = 6.9 × 10^4^ M^−1^ cm^−1^) have been assigned to the π–π* electronic transition in the experimental UV–vis. spectra of L (20 µM in MeOH/H_2_O, 4/1, v/v, HEPES buffer, pH 7.4)^58^. The transition between n and π* is attributed to the 348 nm peak (ε = 5.93 × 10^5^ M^−1^ cm^−1^) is assigned to n-p (symbol of pi)* transition. Common cations such as Li^+^, Na^+^, Mg^2+^, K^+^, Ca^2+^, Al^3+^, V^3+^, Cr^3+^, Mn^2+^, Co^2+^, Ni^2+^, Cu^2+^, Fe^2+^, Fe^3+^, Hg^2+^ and Pb^2+^ remain silent in the context of interaction, reflected in the absorption spectrum (Fig. S17, ESI) (Scheme 2) (Fig. 2).

Three distinct binary mixtures of L and Fe^2+^/Fe^3+^/AsO_2_^−^ at varying pH levels (3.0–12.0) have been used to investigate the impact of pH on spectroscopic characteristics. We conducted all studies at pH 7.4 due to a considerable difference in absorbance between L and three independent systems at pH 7.4 (Fig. S18, ESI).

**Figure 3:**
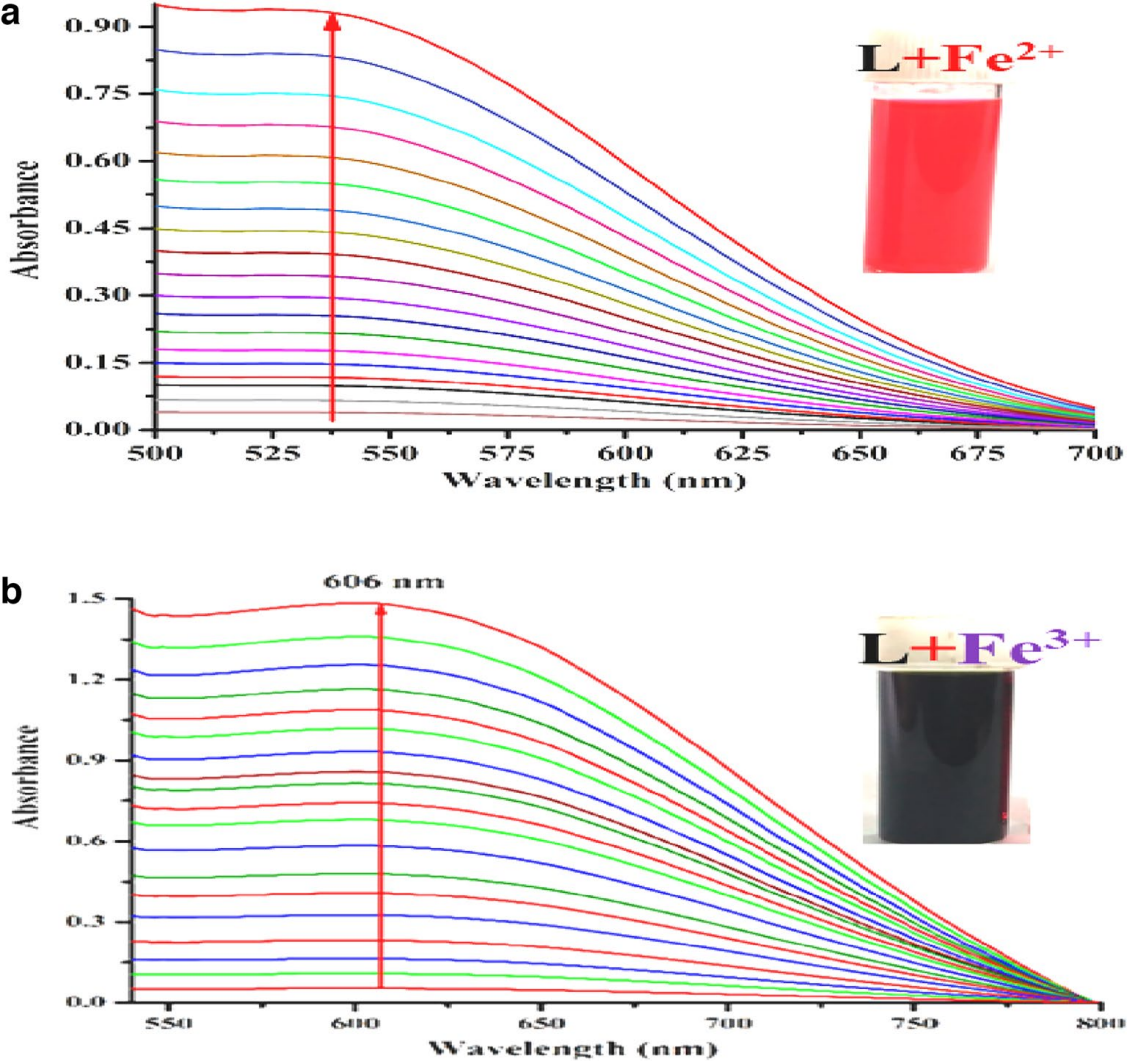
Changes in the absorption spectra of L (20 μM in 20 mM HEPES-buffered MeOH/H_2_O, 4/1, v/v, pH 7.4) with increasing concentration of (**a**) Fe^2+^ and (**b**) Fe^3+^ (0.0, 0.05, 0.1, 0.5, 1.0, 5.0, 10, 20, 50, 75, 100, 200, 300, 500, 1000, 1200, 1600, 2000 and 3000 μM).

Intense blood red and violet colors are generated during spectrophotometric titration of L with Fe^2+^ and Fe^3+^, with peaks (LMCT) located at 538 nm (Fig. 3a) and 606 nm (Fig. 3b), respectively. Additionally, the absorbance at 217 nm and 341 nm (for Fe^2+^) and 225 nm and 349 nm (for Fe^3+^) increased (Figs. S20–S21, ESI). The liner region of the absorbance vs. concentration plot for low levels (ppb) of Fe^2+^ and Fe^3+^ is shown in Fig. S22 (ESI), which can be used to determine the concentrations of these ions. Competitive experiments have been conducted to ensure that no other cations are interfering with the identification of Fe^2+^/^3+^. There are no such disruptions visible in Fig. S23 (ESI). As solvent plays key role on the selectivity of the probe, the absorption and emission characteristics of L in presence of iron(II/III) and arsenite have been investigated in different solvents. The sensitivity and selectivity is optimum for Fe^2+^ and Fe^3+^ in MeOH/H_2_O, (4/1, v/v, pH 7.4), AsO_2_^−^ in HEPES-buffered DMSO/ H_2_O (4/1, v/v, pH 7.4) and hence entire studies has been carried out in this media. (Fig. S46–S47, ESI).

**Scheme 3:**
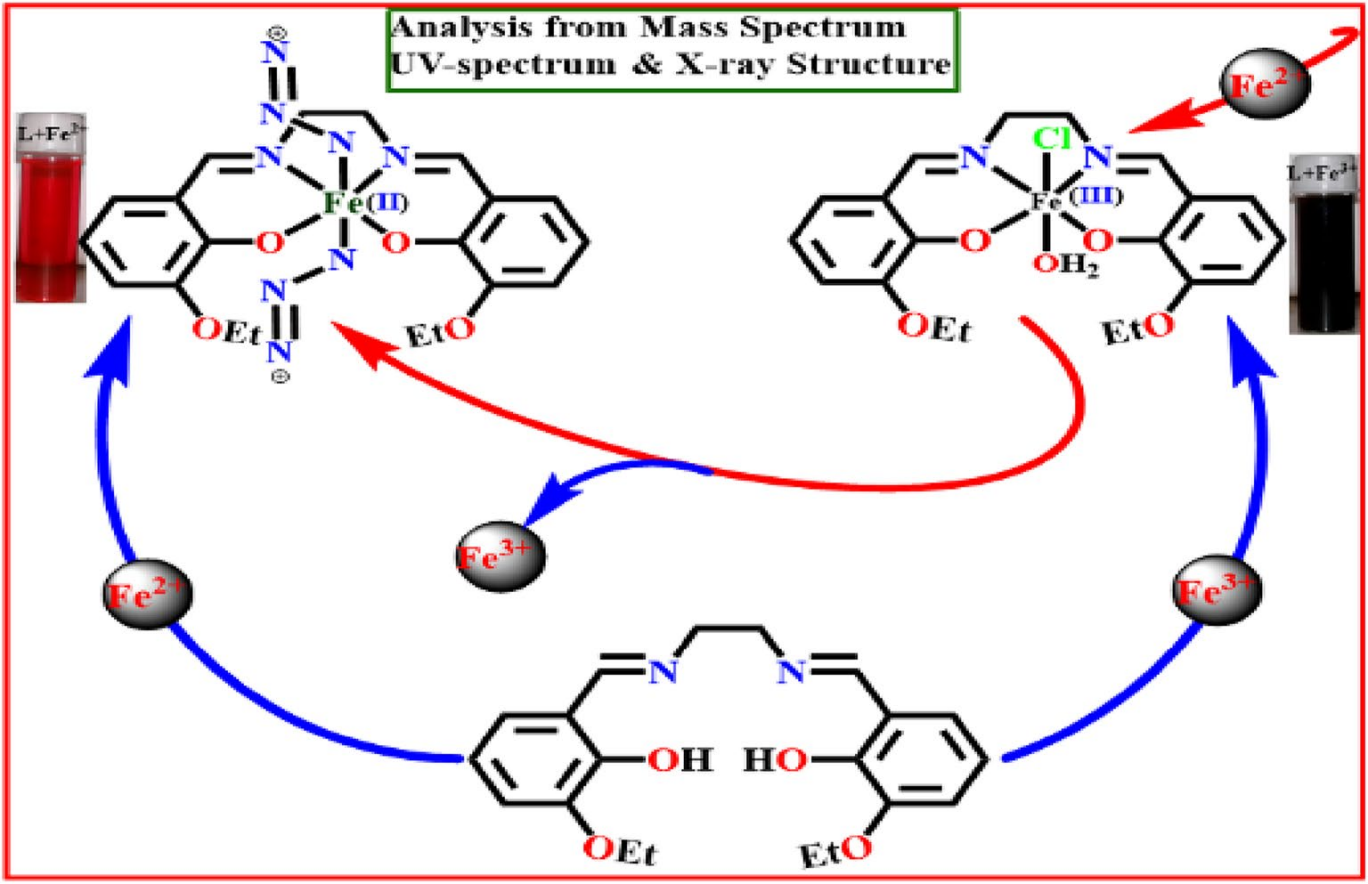
Displacement of Fe^3+^ from [L-Fe^3+^] by Fe^2+^.

**Figure 4:**
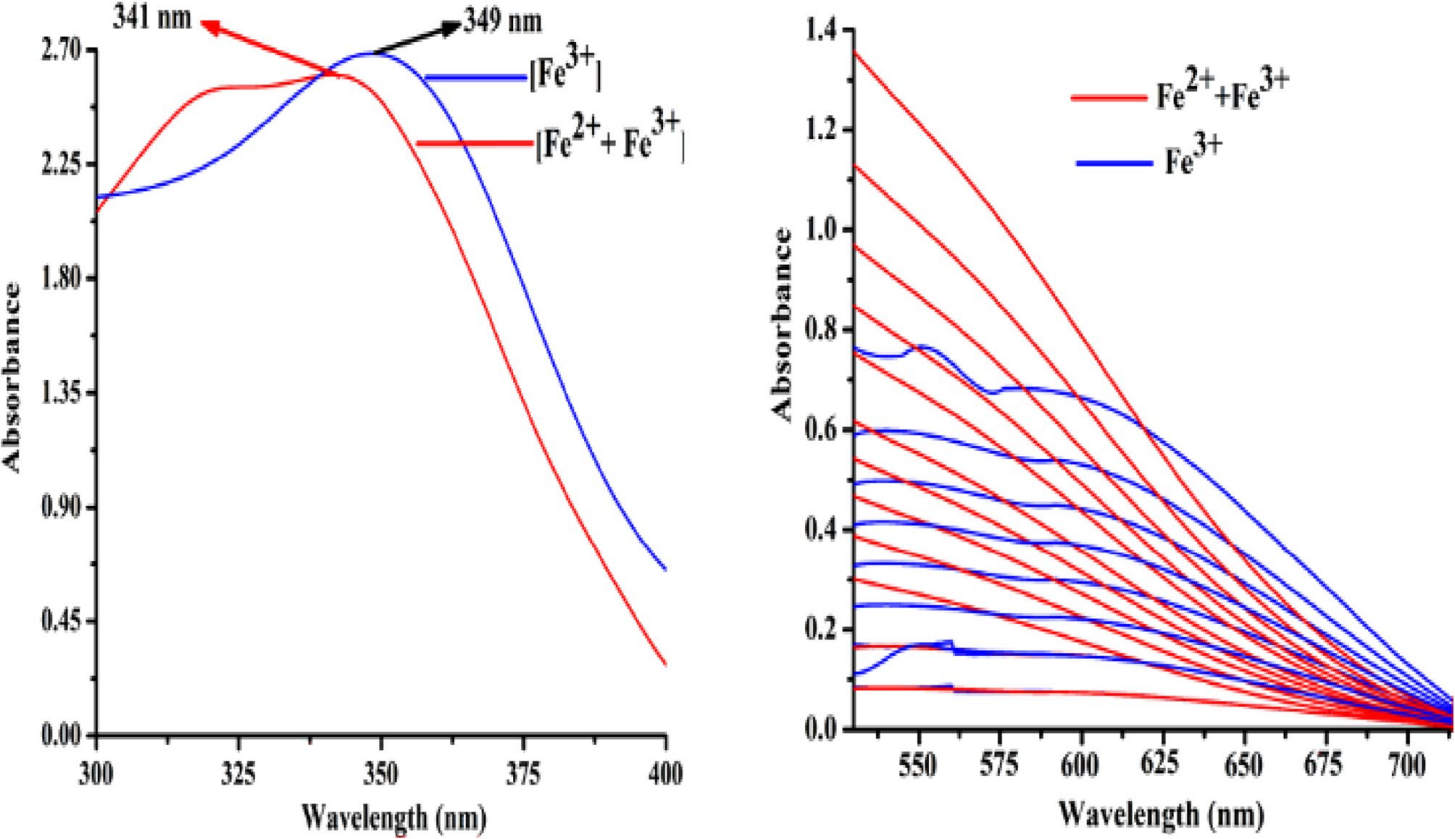
Changes in absorption spectra of [L-Fe^3+^] complex upon gradual addition of Fe^2+^.

As shown in Scheme 3, the formation of the more stable [L-Fe^2+^] complex is facilitated by the increased affinity of Fe^2+^ for L (higher association constant). It follows that the [L-Fe^3+^] combination is more effective Fe^2+^ sensor than L, since it makes use of displacement protocol. Upon gradual addition of Fe^2+^ to a solution of [L-Fe^3+^], the absorption band is shifted from 606 to 538 nm, a blue shift is observed (Fig. 4).

When L is excited at 322 nm in the presence of common metal ions, only Fe^2+^ and Fe^3+^ are able to quench the fluorescence at 414 nm (Fig. S23 (ESI)). The results of competitive studies (supra) to test for any disruptive effects from other common cations during Fe^2+^ and Fe^3+^ identification turned out negative (Fig. S24, ESI). Emission intensity (measured at 414 nm) versus Fe^2+^/Fe^3+^ concentration shows up in Fig. S25 (ESI).The reversible binding behaviour of the designed probe L for Fe^2+^ and Fe^3+^ complex is reflected in absorption spectra as shown in Fig. S46 (ESI).

L forms a 1:1 adducts with Fe^2+^/Fe^3+^, as observed from Job’s plot (Fig. S27, ESI) and confirmed by corresponding mass spectra. Binding constants for Fe^2+^ and Fe^3+^, determined by absorption spectroscopy applying Hill method^59^ has been found 3.29 × 10^6^ M^−1^ and 9.09 × 10^5^ M^−1^, respectively. Both the values are quite similar to the values obtained by the fluorescence method (2.99 × 10^6^ M^−1^ and 3.01 × 10^5^ M^−1^). The L can detect Fe^2+^ and Fe^3+^ concentrations as low as 0.008 µM and 0.005 µM, respectively, as shown in Table S5 (ESI) and Fig. S22 (ESI).

The addition of paramagnetic iron ion further reduces the already weak emission of L at 414 nm, due to the PET process.

Compared to other published probes (Table S5, ESI), the sensitivity and response time of the current probe (L), has been improved. In contrast to the reported probes, our current probe is a “multi-analyte” sensor. Figure S30 (ESI) displays the ‘bare-eye’ colors of L after addition of Fe^2+^ and Fe^3+^ (Fig. 2).

**Scheme 4:**
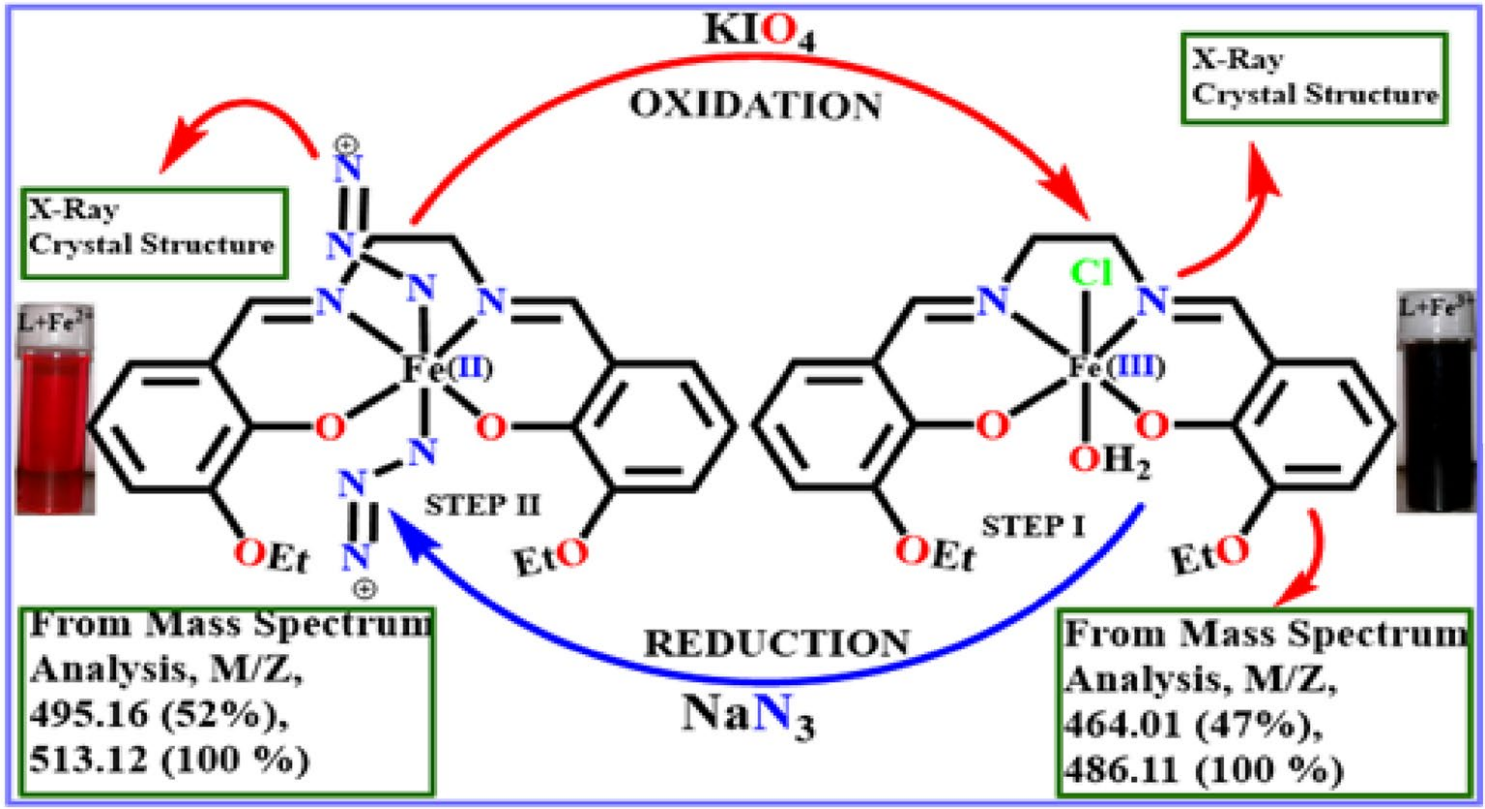
Interconversion between Fe^2+^ and Fe^3+^establishes detection of iron red-ox states in metal complexes with L.

Reversible redox triggering of the L-bound iron centre is visible in bare eye and absorption spectra. Absorption and mass spectroscopy studies corroborate the observation that the addition of aqueous NaN_3_ to the bright violet [L-Fe^3+^] complex in methanol rapidly transforms the solution to blood red, suggesting the conversion of Fe^3+^ to Fe^2+^. However, this vivid blood red solution changes back to its original bright violet color when aqueous KIO_4_ is added to it. It is possible to study inorganic iron speciation owing to the reversible inter-conversion between Fe^3+^/Fe^2+^ complexes of L (Scheme 4).

**Figure 5:**
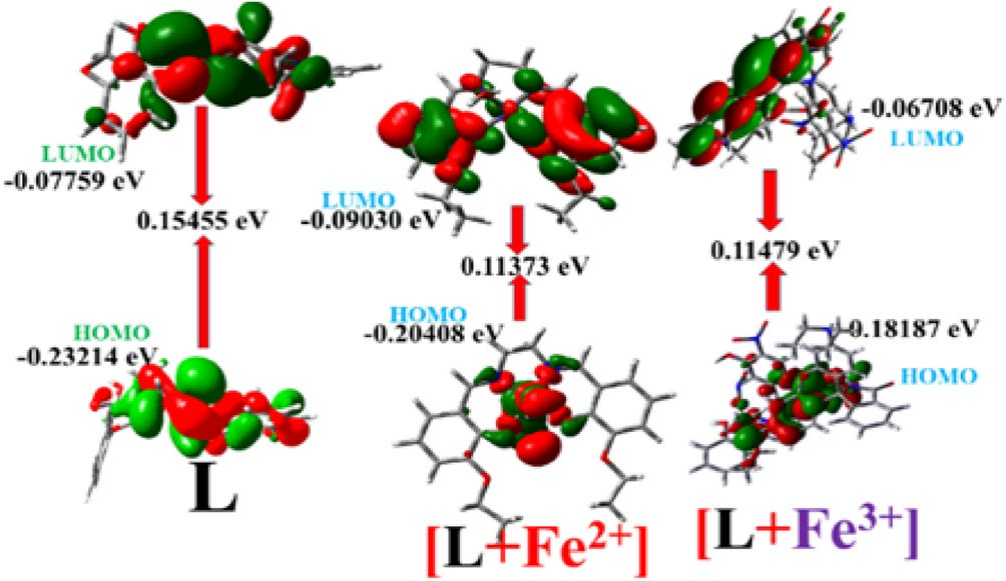
HOMO–LUMO energy gaps of **L** and its Fe^2+^/ Fe^3+^ complexes.

TDDFT calculations^58,60^ have been performed on the systems utilising the B3LYP/LanL2DZ basis set to look into the orbital level interaction and associated energy characteristics. Figure 5 presents the orbital energy diagrams of L and its complexes'. The electronic transition energies (gas phase) are presented in Tables S6–S8 (ESI). When L and Fe^3+^/Fe^2+^ ions combine, the band gap decreases, revealing a red shift in the absorption band.

**Figure 6:**
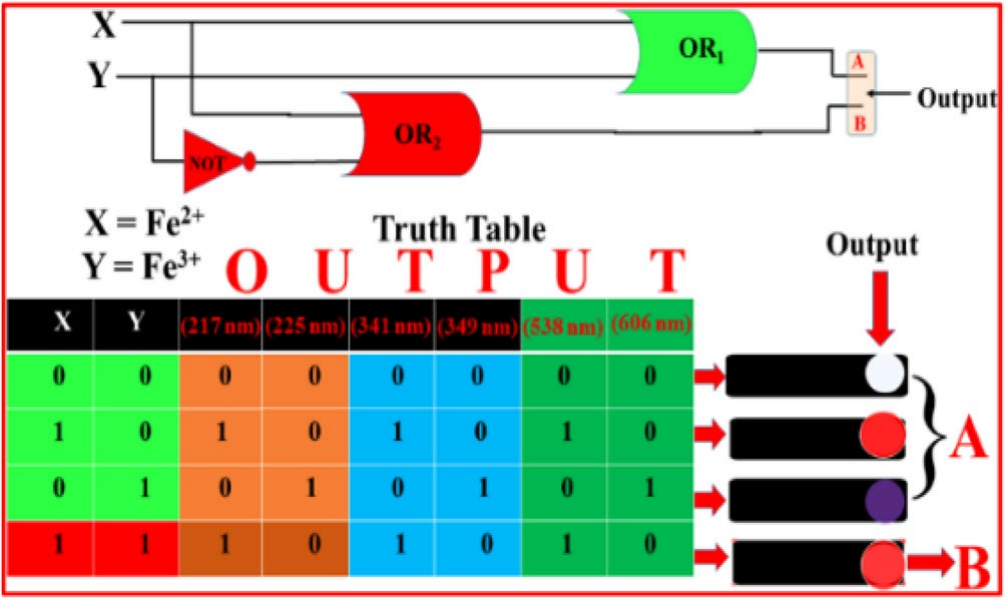
Truth table and logic gate diagram for simultaneous monitoring of Fe^2+^ and Fe^3+^ as input, and absorbance at 217 nm, 225 nm, 341 nm, 349 nm, 538 nm and 606 nm as output.

Employing the developed method, a binary logic gate (Fig. 6) suitable in digital electronics^61^ may be built by modulating the absorption wavelength of L using Fe^2+^ and Fe^3+^ ions. Fe^2+^/Fe^3+^ is the “1” input state, while neither is present is the “0” state. The ‘A’ output of our system is generated by an OR gate (green color) and the ‘B’ output (red color) is generated by a combination OR and NOT gate and a switch that states the scenario explicitly.

## Cyclic voltammetry studies

**Figure 7:**
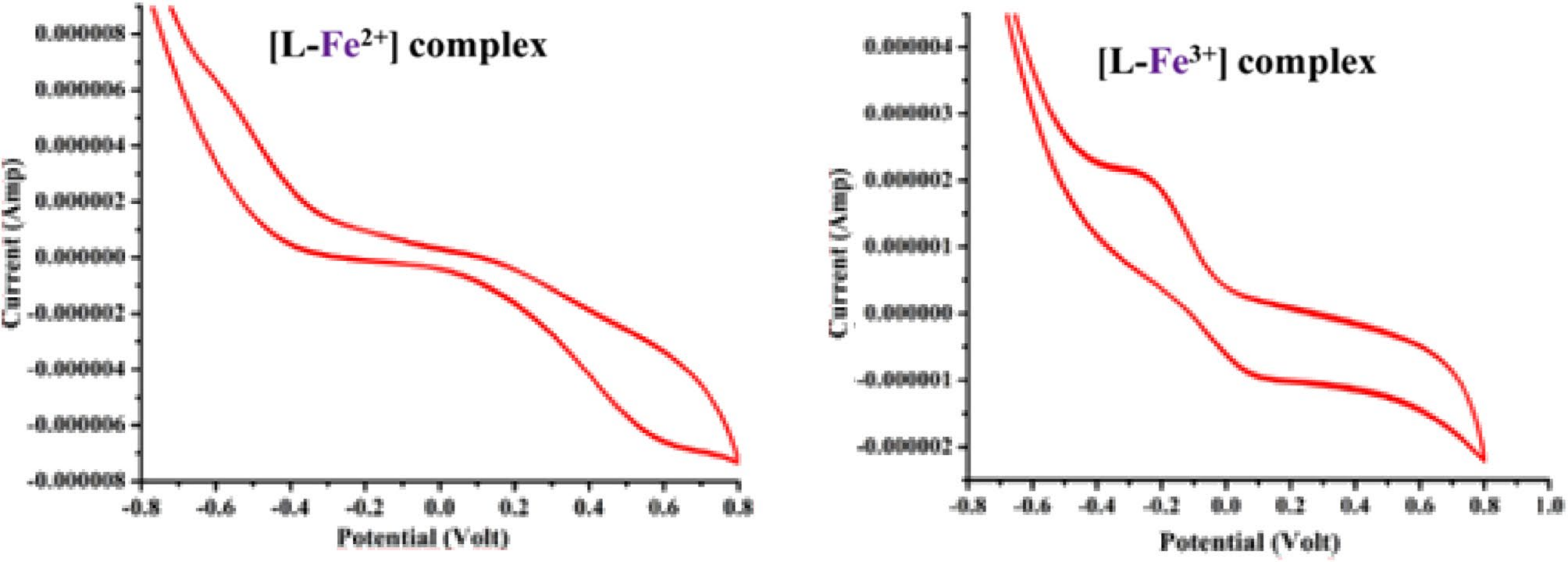
Cyclic voltammograms of [**L-** Iron] complexes in anhydrous dimethyl formamide. Scan rate: 100 mVs^-1^. [L] = 1 × 10^–4^ M, [Fe–L] = 1 × 10^–4^ M, [TBAB] = 0.200 M, T = 30 °C.

To establish that the ligand framework is robust enough to activate the oxidation states of iron, cyclic voltametric (CV) studies have been conducted. It appears that the probe is well suited for its intended function, as CVs recorded in anhydrous dimethyl formamide with Ag/AgCl as reference electrode reveal solely metal-based redox phenomena. Figure 7 displays the CVs of the [L-Fe^2+^] and [L-Fe^3+^] complexes, revealing a quasi-reversible pair with E_1/2_, + 0.59 V and E_1/2_, −0.28 V.

### Fluorescence sensing of arsenite

**Figure 8:**
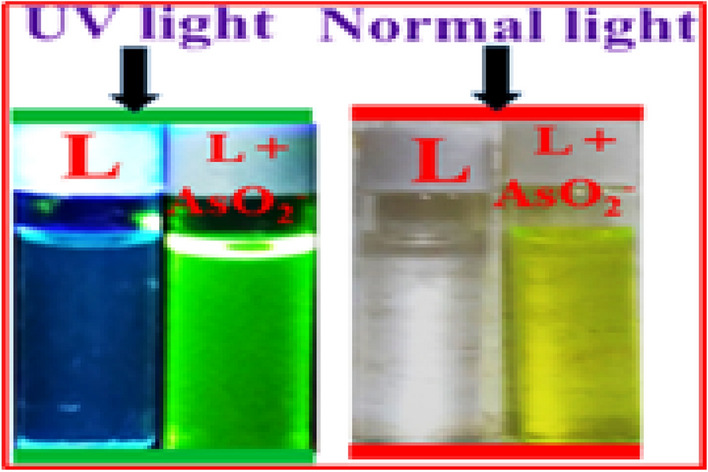
Colors of L under UV and day light after addition of AsO_2_^-^ in HEPES-buffered DMSO/ H_2_O (4/1, v/v, pH 7.4).

**Figure 9:**
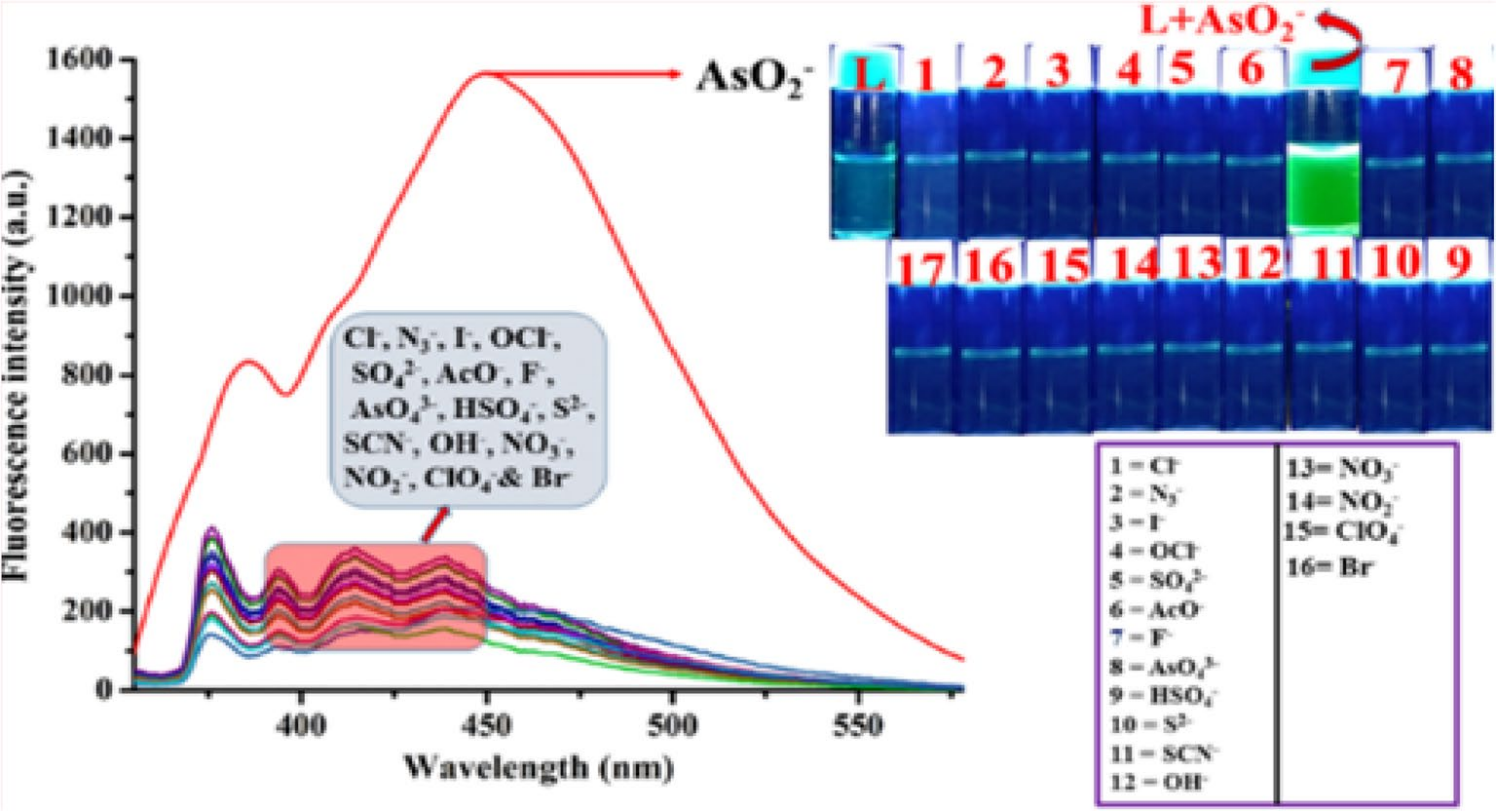
Emission spectra of **L** (20 μM, DMSO/H_2_O, 4/1, v/v, 20 mM HEPES buffer, pH 7.4) in presence of mentioned anions (λ_ex_ = 338 nm).

**Figure 10:**
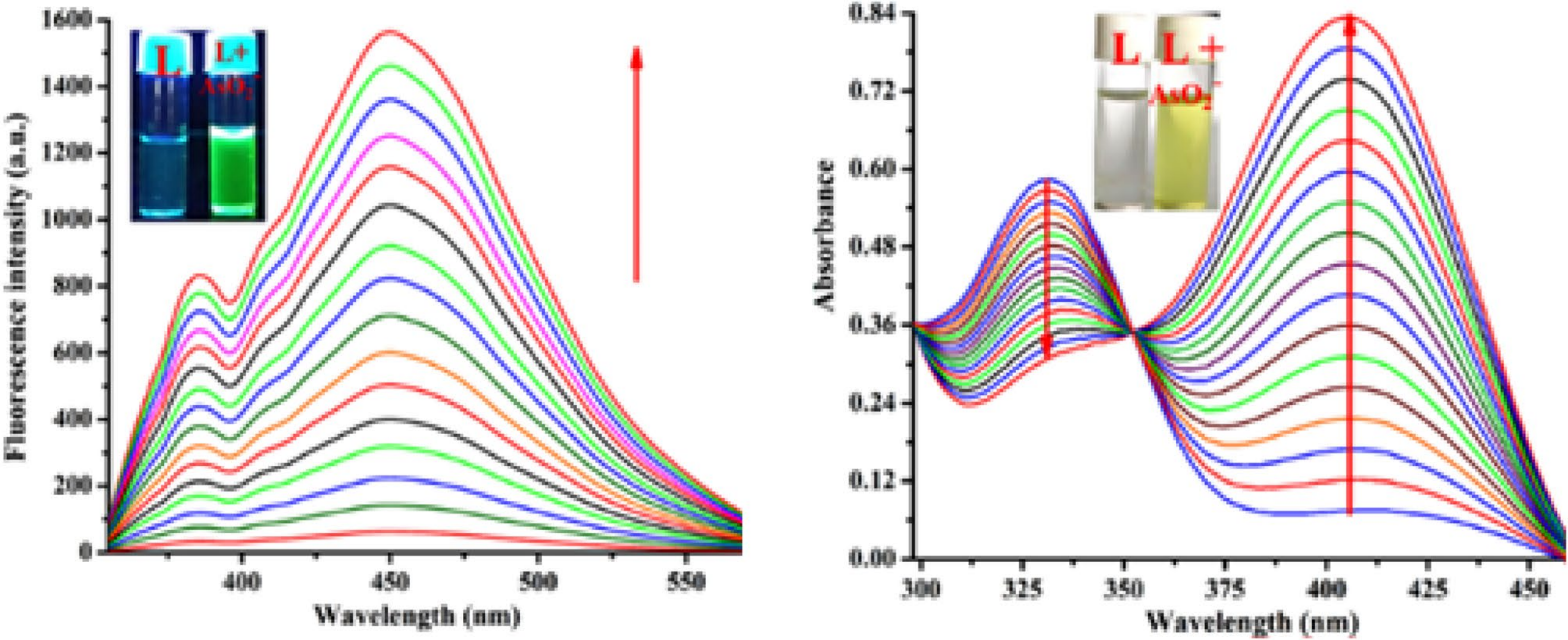
Changes in emission spectra of L (20 μM) in HEPES-buffered (20 mM, DMSO/ H_2_O, 4/1, v/v, pH 7.4) solution upon gradual addition of AsO_2_^−^ (0.0, 0.005, 0.01, 0.05, 0.1, 0.5, 1.0, 2.0, 5.0, 10, 20, 30, 50, 75, 100, 200, 300, 500, 1000, 1500, 2000, 2500 and 3000 μM) (λ_ex_, 338 nm) (left); Corresponding changes in absorption spectra (right).

In aqueous DMSO (DMSO/H_2_O, 4/1, v/v, pH 7.4, Fig. S31, ESI), the addition of AsO_2_^-^ enhances the weak fluorescence of L to intense green at 450 nm (λ_ex_ = 338 nm) (Fig. 8). The pH 7.4 is chosen to perform all experiments as maximum difference in emission intensities between L and its AsO_2_^−^ adduct has been observed at this pH (Fig. S32, ESI). There is little to no interference during AsO_2_^−^ determination (Fig. S33, ESI) in a binary mixture of common anions such as Cl^−^, Br^−^, F^−^, I^−^, SCN^−^, AcO^−^, NO_3_^−^, SO_4_^2−^, NO_2_^−^, ClO_4_^−^, OH^−^, HSO_4_^−^, S^2−^, AsO_4_^3−^, OCl^−^ and N_3_^−^, as revealed from (Fig. 9). In presence of AsO_2_^−^, the emission wavelength of the L is red shifted by 66 nm (λ_ex_, 338 nm, λ_em_, 384 nm). Spectrophotometric and fluorescence titration of L with AsO_2_^−^ is shown in Fig. 10. The emission band at 450 nm shows a sigmoidal growth up to a maximum of 101 fold upon the gradual addition of AsO_2_^−^(0–3000 µM) to L (Fig. S34, ESI). The linear portion of the plot (up to 2.5 µM) of emission intensity vs. [AsO_2_^−^] is prominent in Fig. S35 (ESI), which is helpful for determining low-level AsO_2_^−^. Thus AsO_2_^−^ can be detected as low as 2 × 10^−12^ M, which is far lower than the amount WHO^25^ considers safe for human consumption. The quantum yields of L and its AsO_2_^−^ adduct are 0.017 and 0.431 respectively.

Job’s plot (Fig. S36, ESI) and mass spectra verify that L forms a 1:1 adduct with AsO_2_^−^. Hill equation is used to calculate the binding constant for AsO_2_^−^, which is found to be 1.04 × 10^7^ M^−1^. When viewed in UV light, the [L- AsO_2_^−^] adduct appears green, but in bare eye, it appears green-yellow (Fig. S38, ESI).

The UV-vis. spectrum of L in HEPES-buffered DMSO/H_2_O (4/1, v/v; pH 7.4) solution, reveals an intra-molecular charge transfer (CT) transition at 330 nm. A new CT band appears at 406 nm (Fig. 10, right) that passes through an isobestic point at 348 nm when the formerly colorless L changes to greenish yellow as AsO_2_^−^ is added gradually (0–3000 µM). The changes in absorbance that occur at two distinct wavelengths, 330 nm and 406 nm. when AsO_2_^−^is added to L is shown in Fig. S39a (ESI). The ratio of A_406_/A_330_ increases 2.8-fold (Fig. S39b, ESI). The characteristics of emission and absorption spectra of L in the presence of AsO_2_^−^ vary with solvent (Fig. S47, ESI). Table S9 (ESI) compares the detection limits of the reported probes with the present probe.

**Scheme 5:**
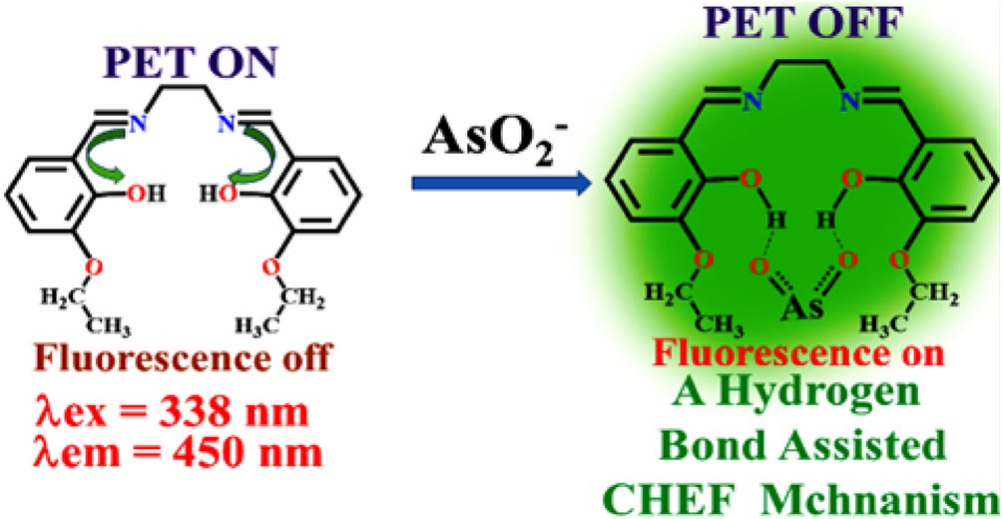
Hydrogen bond assisted AsO_2_^−^ binding with L leading to CHEF.

Scheme 5 shows that the strong hydrogen bond between L and AsO_2_^−^ is responsible for CHEF, at the cost of PET inhibition in L.

**Figure 11:**
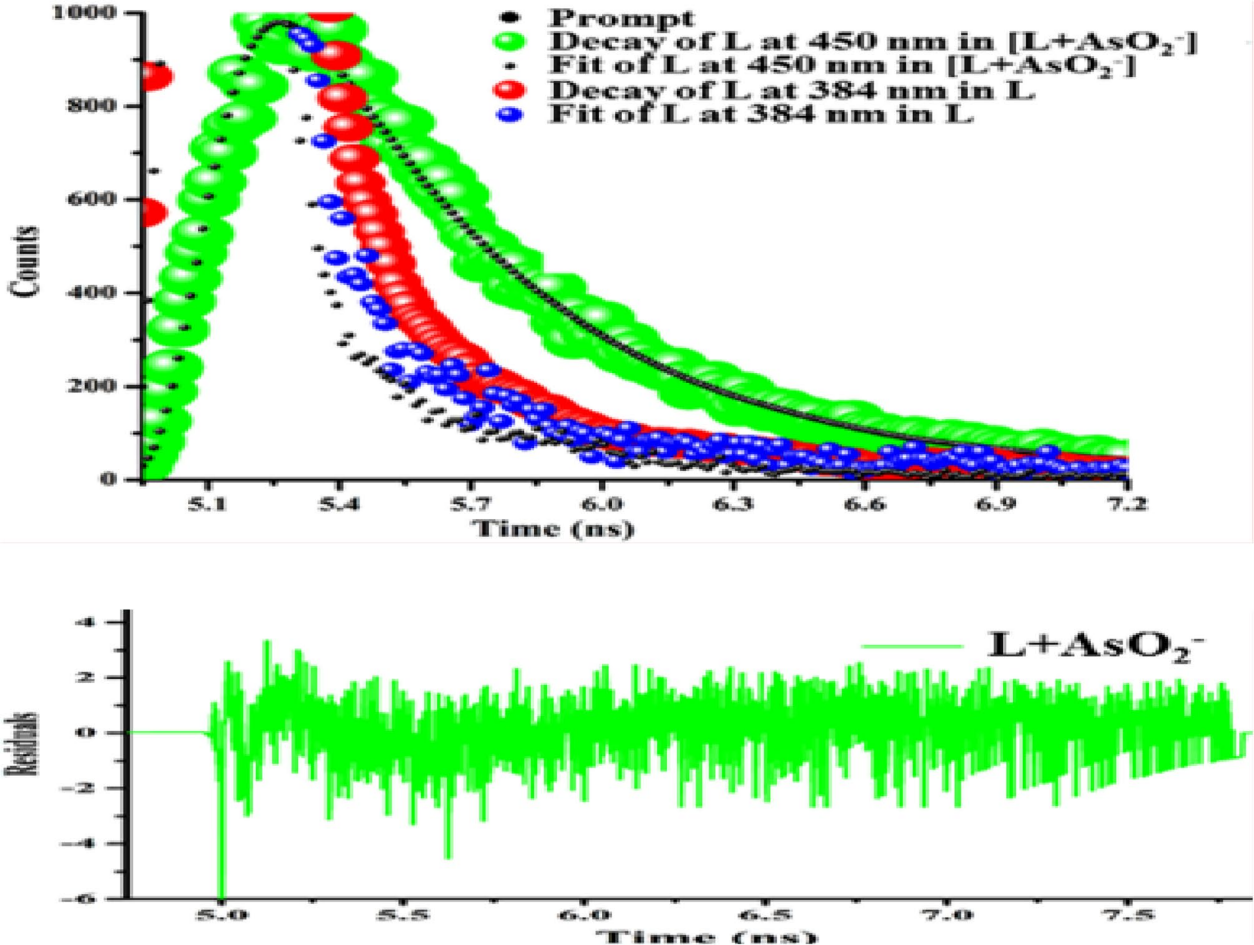
Time resolved fluorescence decay of L in absence and presence of AsO_2_^−^ in the medium mentioned *supra*, corresponding residual plot, *infra* (λ_ex_ = 338 nm, λ_em_ = 450 nm).

According to decay studies, the fluorescence lifetime of L increased in presence of AsO_2_^−^ from 0.2664 ns to 1.1208 ns (Table S10, ESI) (Fig. 11). Additional support for the speculated sensing mechanism is provided by constructing a receptor L1 (Figs. S39–S40, ESI) devoid of the phenol -OH group by replacing 3-ethoxy salicylaldehyde by benzaldehyde (Scheme S1, ESI). It has been determined (Fig. S42, ESI) that L1 has a negligible effect on AsO_2_^−^. Therefore, presence -OH group is crucial for AsO_2_^−^ recognition.

**Figure 12:**
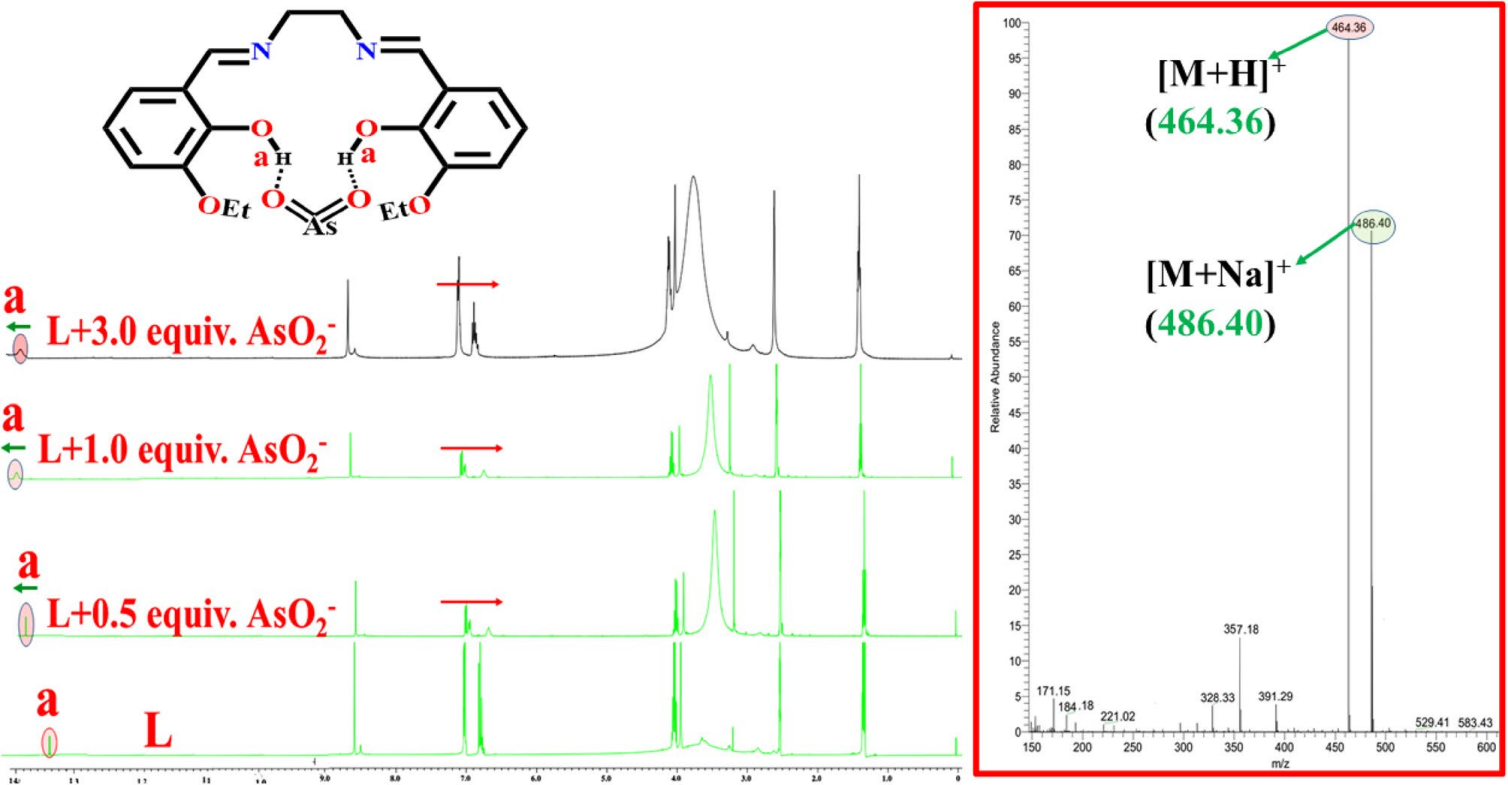
^1^HNMR titration of L with AsO_2_^−^ in DMSO-d_6_; QTOF-MS spectrum of [L-AsO_2_^−^] adduct (inset).

Due to participation in H-bonding with AsO_2_^−^, the IR band at 3221 cm^-1^ of L is shifted to 3293 cm^-1^ in the [L- AsO_2_^−^] adduct, showing a weakening of the O–H bond (of the salicylaldehyde moiety) (Fig. S43, ESI). The interaction between L and AsO_2_^−^ has also been confirmed by ^1^HNMR specturm in DMSO-d_6_ (Fig. 12). The addition of 0.5 equiv. AsO_2_^−^ to L caused an up-field shift in all aromatic proton, but no change in the position of the CH=N proton (8.6 ppm). This result indicates that the imine nitrogen is not participating in hydrogen bonding with AsO_2_^−^. A small up-field shift occurred in the aliphatic protons. As a result of interaction with the AsO_2_^−^ ion, the –OH proton of the 3-ethoxysalicyaldehyde moiety shifted down field by 0.286 ppm. The –OH proton of the 3-ethoxysalicyaldehyde moiety shifted down-field by 0.293 ppm when 1 equiv. AsO_2_^−^ was added to L, supporting their interaction.

All these findings, including the ESI-mass spectrometry studies (Fig. 12 and Fig. S9, ESI), indicate strong hydrogen bonds between the AsO_2_^−^ ion and the phenol –OH functionality. With the addition of AsO_2_^−^ ions, the molecular ion peak of L shifts from its original position at m/z, 357.18 for [M + H]^+^ to m/z, 464.36 for [M + H]^+^ and m/z, 486.40 for [M + Na]^+^, providing strong evidence for a 1:1 interaction (mole ratio) between L and AsO_2_^−^ ions (Fig. 12 (in set) and Fig. S9, ESI).

**Figure 13:**
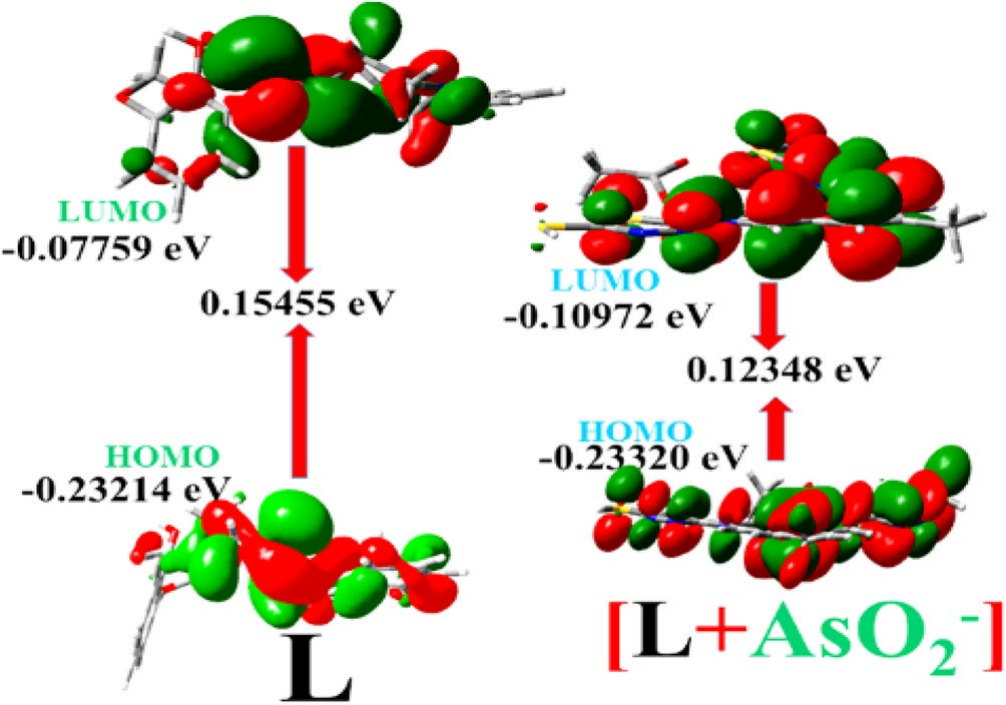
HOMO–LUMO energy gaps in L and its AsO_2_^−^ adduct.

Gaussian 09W is used to perform time-dependent density functional theoretical calculations (TDDFT) in the gas phase for optimal geometries of L and its AsO_2_^−^ adduct using the B3LYP (Becke's three-parameter hybrid functional implementing the LYP correlation functional)/LanL2DZ basis set. In the presence of AsO_2_^−^ ion, the HOMO and LUMO energy gap in L reduces from 0.15455 eV to 0.03107 eV (Fig. 13). Tables S6 and S11 (ESI) list the electronic transition energies that are most favourable for the most ubiquitous absorption bands.

### Application

#### Iron speciation studies

In the presence of Fe^2+^ and Fe^3+^ ions, L takes on two distinct and unique hues. The maximum absorption in case of Fe^2+^ is at 538 nm, while it is 606 nm for Fe^2+^. Probe L is utilized for the analysis of Fe^2+^ and Fe^3+^ in real samples^62,63^. Recovery procedures of the aforementioned ions are carried out in real samples, spiked with those metal ions at various concentrations in order to evaluate the efficacy of the approach. The methods used to prepare and analyse samples have been laid forth in detail in the ESI. Thus the developed, spectrophotometric method for iron speciation allows the detection and quantitative determination of both redox states of iron in a sample (Table 1, Fig. S44, ESI).

**Table 1:** Determination of unknown Fe^2+^ in real water using L.

Sample	Amount (µM)	Absorbance	Found (µM)	Recovery (%)
Mixture of Fe^2+^ and Fe^3+^	100	0.15	47	–
Mixture of Fe^2+^ and Fe^3+^, after KIO_4_ oxidation	100	0.31	95	95 ± 1.23
Only Fe^3+^	50	0.21	48	96 ± 1.20
Only Fe^2+^	50	0.16	47	94 ± 1.33

#### Discrimination of Fe^2+^ and Fe^3+^ in a mixture

In the laboratory, ferrocyanide and ferricyanide are commonly used for the qualitative detection of Fe^3+^ and Fe^2+^ in a mixture, respectively. Table S12 summarizes the available strategies for the ‘bare-eye’ detection/discrimination of Fe^2+^ and Fe^3+^, along with their limitations. Since Fe^2+^ and Fe^3+^ have different absorption maxima even at the ppb level, the present probe produces two distinct colors. With Fe^2+^, it turns intense red and with Fe^3+^, it turns intense violet.

### Determination of AsO_2_^−^ in real water

AsO_2_^−^ in real water samples has been determined using the conventional method (Table 2). Recovery AsO_2_^−^ at varying concentrations have been conducted to assess the reliability of the technique. The standard addition method is used to analyze the water sample collected from the industrial area of Durgapur-Asansol in West Bengal, India (Table 3). Separate experiments (Table 4) show that the probe can be used to determine arsenate(V) by oxidizing arsenite under suitable conditions^48,64^. These findings are significant advancement on the existing methods of analysis.

**Table 2:** Determination of AsO_2_^−^ in tap water using L.

Sample	Added (10^–6^ M)	Found (10^–6^ M)	Recovery (%)
T1	6.01	5.52	91.84 ± 1.03
T2	6.30	5.55	88.09 ± 1.13
T3	7.30	6.70	91.78 ± 1.11

**Table 3:** Determination of AsO_2_^−^ in industrial water using L.

Sample	Added (10^–6^ M)	Found (10^–6^ M)	Recovery (%)
R1	6.01	5.95	99 ± 1.11
R2	6.30	6.21	98 ± 1.03
R3	7.30	7.22	99 ± 1.12

**Table 4:** Determination of AsO_3_^−^ in tap water after treatment with Fenton's reagent.

Sample	Amount (µM)	Emission intensity (a. u.)	Found (µM)	Recovery (%)
Mixture of AsO_3_^−^ and AsO_2_^−^	50.10	75.12	24.12	–
Mixture of AsO_3_^−^ and AsO_2_^−^ after treatment with Fenton's reagent	50.09	10.23	48.03	96 ± 2.10 (quenching )
Only AsO_2_^−^	25.05	80.21	24.50	98 ± 2.11
Only AsO_3_^−^	25.07	7.08	–	–

### Solid-phase extractive removal of arsenite

Immobilisation of L on silica (100–200 mesh), following published procedure has been attempted to remove toxic arsenite(III) from its reservoir^65^. Comparison of the IR spectra of L immobilised silica prior and and post-sorption of arsenite(III) indicate a significant change (red-shift) of the characteristics functionalities (Fig. S45, ESI). The data relevant to the removal of arsenite(III) from its reservoir by silica-immobilized L is presented in Table 5.

**Table 5:** Solid-phase extractive removal of arsenite.

Sample	Added (10^–2^ M)	Found (10^–2^ M)	Recovery (%)
P1	7.01	6.92	99 ± 1.29
P2	11.20	10.90	97 ± 1.20
P3	13.12	12.95	99 ± 1.21

## Conclusion

A low cost method for instantaneous optical detection and quantitative determination of Fe ^2+^, Fe ^3+^ and AsO_2_^−^ in mixed aqueous organic media is established. Single crystal X-ray structures of the resulting iron complexes firmly establish the binding event of the probe with iron. The AsO_2_^−^ assisted 91-fold fluorescence enhancement of the probe is attributed to the inhibition of PET as a result of H-bond assisted CHEF process. Thus, the probe is suitable for solvent-dependent detection of both cation and anion at trace level which is a significant achievement. The detection and monitoring of concentration of Fe^2+^, Fe^3+^ and AsO_2_^−^ in real samples have been achieved.

### Supplementary Information

Supplementary Information.

## References

[1] Bell TW, Hext NM (2004). Supramolecular optical chemosensors for organic analytes. Chem. Soc. Rev..

[2] Suksai C, Tuntulani T (2003). Chromogenic anion sensors. Chem. Soc. Rev..

[3] Lehn JM (1985). Supramolecular chemistry: Receptors, catalysts, and carriers. Science.

[4] Stadler AM, Lehn JM (2014). Coupled nanomechanical motions: Metal-ion-effected, pH-modulated, simultaneous extension/contraction motions of double-domain helical/linear molecular strands. J. Am. Chem. Soc..

[5] Kumar A, Chhatwal MP, Singh AK, Singh V, Trivedi M (2014). A fast and selective probe for monitoring Pd^2+^ in aqueous medium via dual-optical readout. Chem. Commun..

[6] Kumar A, Chhatwal MP, Mondal C, Singh V, Singh AK, Cristaldi DA, Gupta RD, Gulino A (2014). A ternary memory module using low-voltage control over optical properties of metal-polypyridyl monolayers. Chem. Commun..

[7] McConnell AJ, Wood CS, Neelakandan PP, Nitschke JR (2015). Stimuli-responsive metal-ligand assemblies. Chem. Rev..

[8] Liang ZQ, Wang CX, Yang JX, Gao HW, Tian YP, Tao XT, Jiang MH (2007). A highly selective colorimetric chemosensor for detecting the respective amounts of iron (II) and iron (III) ions in water. New J. Chem..

[9] Sen S, Sarkar S, Chattopadhyay B, Moirangthem A, Basu A, Dhara K, Chattopadhyay P (2012). A ratiometric fluorescent chemosensor for iron: discrimination of Fe^2+^ and Fe^3+^ and living cell application. Analyst.

[10] Sivaraman G, Anand T, Chellappa D (2014). Pyrene based selective–ratiometric fluorescent sensing of zinc and pyrophosphate ions. Anal. Methods.

[11] Sivaraman G, Vidya B, Chellappa D (2014). Rhodamine based selective turn-on sensing of picric acid. RSC Adv..

[12] Dunn LL, Rahmanto YS, Richardson DR (2007). Iron uptake and metabolism in the new millennium. Trends Cell Biol..

[13] Weizman HH, Ardon O, Mester B, Libman J, Dwir O, Hadar Y, Chen Y, Shanzer A (1996). Fluorescently-labeled ferrichrome analogs as probes for receptor-mediated, microbial iron uptake. J. Am. Chem. Soc..

[14] Halliwell B, Chirico S (1993). Lipid peroxidation: Its mechanism, measurement, and significance. Am. J. Clin. Nutr..

[15] Walter PB (2002). Iron deficiency and iron excess damage mitochondria and mitochondrial DNA in rats. Proceed. Natl. Acad. Sci..

[16] Mei Q, Jiang C, Guan G, Zhang K, Liu B, Liu R, Zhang Z (2012). Fluorescent graphene oxide logic gates for discrimination of iron (3+) and iron (2+) in living cells by imaging. Chem. Commun..

[17] Ercal N, Gurer-Orhan H, Aykin-Burns N (2001). Toxic metals and oxidative stress part I: Mechanisms involved in metal-induced oxidative damage. Curr. Topics Med. Chem..

[18] Ta S, Nandi S, Ghosh M, Banerjee S, Das D (2017). Pyridine–antipyrine appended indole derivative for selective recognition of Fe^3+^: Concentration dependent coloration. Spectrochim. Acta, Part A.

[19] Wang J, Pantopoulos K (2011). Regulation of cellular iron metabolism. Biochem. J..

[20] Burdo JR, Connor JR (2003). Brain iron uptake and homeostatic mechanisms: An overview. Biometals.

[21] Martínez-Máñez R, Sancenón F (2003). Fluorogenic and chromogenic chemosensors and reagents for anions. Chem. Rev..

[22] Hasegawa H, Matsui M, Okamura S, Hojo M, Iwasaki N, Sohrin Y (1999). Arsenic speciation including ‘hidden’ arsenic in natural waters. Appl. Organomet. Chem..

[23] Cullen WR, Reimer KJ (1989). Arsenic speciation in the environment. Chem. Rev..

[24] Kaur H, Kumar R, Babu JN, Mittal S (2015). Advances in arsenic biosensor development–a comprehensive review. Biosens. Bioelectron..

[25] Yogarajah NH, Tsai SS (2015). Detection of trace arsenic in drinking water: Challenges and opportunities for microfluidics. Environ. Sci. Water Res. Technol..

[26] Hug SJ, Canonica L, Wegelin M, Gechter D, von Gunten U (2001). Solar oxidation and removal of arsenic at circumneutral pH in iron containing waters. Environ. Sci. Technol..

[27] Tokar EJ, Diwan BA, Waalkes MP (2010). Arsenic exposure transforms human epithelial stem/progenitor cells into a cancer stem-like phenotype. Environ. Health Perspect..

[28] Raizada M, Sama F, Ashafaq M, Shahid M, Khalid M, Ahmad M, Siddiqi ZA (2018). Synthesis, structure and magnetic studies of lanthanide metal–organic frameworks (Ln–MOFs): Aqueous phase highly selective sensors for picric acid as well as the arsenic ion. Polyhedron.

[29] Richardson SD (1999). Water analysis. Anal. Chem..

[30] Keimowitz AR, Mailloux BJ, Cole P, Stute M, Simpson HJ, Chillrud SN (2007). Laboratory investigations of enhanced sulfate reduction as a groundwater arsenic remediation strategy. Environ. Sci. Technol..

[31] Mitra A, Ramanujam B, Rao CP (2009). 1-(d-Glucopyranosyl-2′-deoxy-2′-iminomethyl)-2-hydroxynaphthalene as chemo-sensor for Fe^3+^ in aqueous HEPES buffer based on colour changes observable with the naked eye. Tetrahedron Lett..

[32] Wolf C, Mei X, Rokadia HK (2004). Selective detection of Fe(III) ions in aqueous solution with a 1,8-diacridylnaphthalene-derived fluorosensor. Tetrahedron Lett..

[33] Lohani CR, Lee KH (2010). The effect of absorbance of Fe^3+^ on the detection of Fe^3+^ by fluorescent chemical sensors. Sens. Actuators B.

[34] Singh N, Kaur N, Dunn J, MacKay M, Callan JF (2009). A new fluorescent chemosensor for iron (III) based on the β-aminobisulfonate receptor. Tetrahedron Lett..

[35] Lee DY, Singh N, Jang DO (2011). Fine tuning of a solvatochromic fluorophore for selective determination of Fe^3+^: A new type of benzimidazole-based anthracene-coupled receptor. Tetrahedron. Lett..

[36] Lin W, Long L, Yuan L, Cao Z, Feng J (2009). A novel ratiometric fluorescent Fe^3+^ sensor based on a phenanthroimidazole chromophore. Anal. Chim. Acta.

[37] Zhang M, Gao Y, Li M, Yu M, Li F, Li L, Zhu M, Zhang J, Yi T, Huang C (2007). A selective turn-on fluorescent sensor for Fe^III^ and application to bioimaging. Tetrahedron. Lett..

[38] Wang S, Meng X, Zhu M (2011). A naked-eye rhodamine-based fluorescent probe for Fe (III) and its application in living cells. Tetrahedron Lett..

[39] Kumar M, Kumar R, Bhalla V, Sharma PR, Kaur T, Qurishi Y (2011). Thiacalix[4]arene based fluorescent probe for sensing and imaging of Fe^3+^ ions. Dalton Trans..

[40] Zhan J, Wen L, Miao F, Tian D, Zhu X, Li H (2012). Synthesis of a pyridyl-appended calix [4] arene and its application to the modification of silver nanoparticles as Fe^3+^ colorimetric sensor. New J. Chem..

[41] Sahoo SK, Sharma D, Bera RK, Crisponi G, Callan JF (2012). Iron (III) selective molecular and supramolecular fluorescent probes. Chem. Soc. Rev..

[42] Liu JM, Zheng QY, Yang JL, Chen CF, Huang ZT (2002). A new fluorescent chemosensor for Fe^3+^ and Cu^2+^ based on calix [4] arene. Tetrahedron Lett..

[43] Ocak Ü, Ocak M, Surowiec K, Liu X, Bartsch RA (2009). Metal ion complexation in acetonitrile by upper-rim allyl-substituted, di-ionized calix [4] arenes bearing two dansyl fluorophores. Tetrahedron.

[44] Bricks JL, Kovalchuk A, Trieflinger C, Nofz M, Büschel M, Tolmachev AI, Daub J, Rurack K (2005). On the development of sensor molecules that display FeIII-amplified fluorescence. J. Am. Chem. Soc..

[45] Xiang Y, Tong A (2006). A new rhodamine-based chemosensor exhibiting selective feiii-amplified fluorescence. Org. Lett..

[46] Mao J, Wang L, Dou W, Tang X, Yan Y, Liu W (2007). Tuning the selectivity of two chemosensors to Fe (III) and Cr (III). Org. Lett..

[47] Adhikari S, Ghosh A, Ghosh M, Guria S, Das D (2017). Ratiometric sensing of Fe^3+^ through PET-CHEF-FRET processes: Live cell imaging, speciation and DFT studies. Sens. Actuators, B.

[48] Ghosh M, Mandal S, Ta S, Das D (2017). Detection and discrimination of Al^3+^ and Hg^2+^ using a single probe: Nano-level determination, human breast cancer cell (MCF7) imaging, binary logic gate development and sea fish sample analysis. Sens. Actuators.

[49] Pathak RK, Dessingou J, Hinge VK, Thawari AG, Basu SK, Rao CP (2013). Quinoline driven fluorescence turn on 1,3-Bis-calix [4] arene conjugate-based receptor to discriminate Fe^3+^ from Fe^2+^. Anal. Chem..

[50] Yadav N, Singh AK (2016). Dual anion colorimetric and fluorometric sensing of arsenite and cyanide ions. RSC Adv..

[51] Lohar S, Pal S, Sen B, Mukherjee M, Banerjee S, Chattopadhyay P (2014). Selective and sensitive turn-on chemosensor for arsenite ion at the ppb level in aqueous media applicable in cell staining. Anal. Chem..

[52] Lakowicz, J. R. Instrumentation for Fluorescence Spectroscopy. in *Principles of Fluorescence Spectroscopy* (ed. Lakowicz, J. R.) 25–61 (Springer US, 1999). 10.1007/978-1-4757-3061-6_2.

[53] Goldys, E. M. *Fluorescence Applications in Biotechnology and Life Sciences.* (John Wiley & Sons, 2009).

[54] Giepmans BNG, Adams SR, Ellisman MH, Tsien RY (2006). The fluorescent toolbox for assessing protein location and function. Science.

[55] Baker A (2001). Fluorescence excitation−emission matrix characterization of some sewage-impacted rivers. Environ. Sci. Technol..

[56] A, T. Hormone synthesis: Thyroid iodine metabolism. *Werner and Ingbar’sThe Thyroid* (1996)

[57] Bauer M, London ED, Silverman DHS, Rasgon N, Kirchheiner J, Whybrow PC (2003). Thyroid, brain and mood modulation in affective disorder: insights from molecular research and functional brain imaging. Pharmacopsychiatry.

[58] Ghosh A, Ta S, Ghosh M, Karmakar S, Banik A, Dangar TK, Mukhopadhyay SK, Das D (2015). Dual mode ratiometric recognition of zinc acetate: nanomolar detection with in vitro tracking of endophytic bacteria in rice root tissue. Dalton Trans..

[59] Ghosh M, Ghosh A, Ta S, Matalobos JS, Das D (2017). ESIPT-based nanomolar Zn2+ sensor for human breast cancer cell (MCF7) imaging. ChemistrySelect.

[60] Kumari B, Lohar S, Ghosh M, Ta S, Sengupta A, Banerjee PP, Chattopadhyay A, Das D (2016). Structurally characterized Zn^2+^ selective ratiometric fluorescence probe in 100% water for HeLa cell imaging: experimental and computational studies. J. Fluores..

[61] Rai A, Singh AK, Sonkar AK, Prakash A, Roy JK, Nagarajan R, Mishra L (2016). A smart switchable module for the detection of multiple ions via turn-on dual-optical readout and their cell imaging studies. Dalton Trans..

[62] Skoog, D. A., West, D. M., Holler, F. J. & Crouch, S. R*. Fundamentals of Analytical Chemistry*. (Cengage Learning, 2013).

[63] Welcher FJ (1963). A text-book of quantitative inorganic analysis including elementary instrumental analysis (Vogel, Arthur I.). J. Chem. Educ..

[64] Nandi S, Das D (2016). Smart probe for multianalyte signaling: Solvent dependent selective recognition of I^–^. ACS Sens..

[65] Mahmoud ME, Al Saadi MSM (2001). Selective solid phase extraction and preconcentration of iron (III) based on silica gel-chemically immobilized purpurogallin. Anal. Chim. Acta.

