# Supplementary material for: A single probe for solvent dependent optical recognition of iron(II/III) and arsenite: discrimination between iron redox states with single crystal X-ray structure evidence
Source: Sci Rep. 2023 Oct 21;13:18039. doi: 10.1038/s41598-023-43154-2 (PMC10590385; doi:10.1038/s41598-023-43154-2)
Supplement: Supplementary file 1 — Supplementary Information. [file 41598_2023_43154_MOESM1_ESM.docx]

**Supporting Information**

A single probe for solvent dependent optical recognition of iron(II/III) and arsenite: discrimination between iron redox states with single crystal X-ray structure evidence

Jayanta Das, Milan Ghosh, Prasenjit Mandal, Sangita Maji and Debasis Das*

Department of Chemistry, The University of Burdwan, Burdwan, 713104, West Bengal, India

Correspondence: <ddas100in@yahoo.com>; phone, +91-342- 2533913; fax, +91-342-2530452

**1. General method of UV-Vis. and fluorescence titration**

Absorption and emission spectroscopic studies have been performed using cells of 1cm path length. The stock solutions of L (20 µM) for UV-Vis and fluorescence studies have been prepared in HEPES (20 µM) buffered MeOH/ H_2_O (4/1, v/v, pH 7.4) and DMSO/ H_2_O (4/1, v/v, pH 7.4) respectively. Corresponding working solutions have been prepared from the stock solutions by appropriate dilution. The slit width used for fluorescence measurement is 5 nm × 5 nm. All the spectral data have been collected after a waiting period of 10 minutes of mixing the constituents.

**2. Determination of quantum yield**

Fluorescence quantum yield (Ф) is estimated by integrating the area under the fluorescencecurves using the equation.^1^


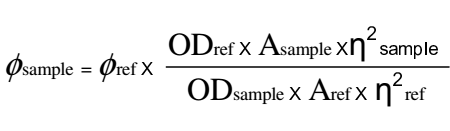


Where A is the area under the emission spectrum and OD is the optical density of the compound at the excitation wavelength, Ƞ is the refractive index of the solvent. In case of L, the fluorescence quantum yield has been calculated in absence and presence of AsO_2_^-^ in DMSO/H_2_O (4/1, v/v, pH 7.4) HEPES (20 µM) buffer.

**3. Job’s plot from fluorescence/ absorbance experiment**

A series of solutions containing **L**, FeCl_3_.6H_2_O/ Mohr salt are prepared such that the total concentration of the respective ions and **L** remain constant (20 µM) in all the sets. The mole fraction (X) of **L** is varied from 0.1 to 0.9. The absorbance/ emission are plotted against the mole fraction of the **L** in solution. Job’s plot revealed that maximum absorbance/ emission at 1:1 (mole ratio) for all the three systems. The corresponding binding constant values are calculated using Hill equation.

**4**. **Determination of detection limit**

To determine the detection limit, fluorescence or UV-Vis. titration of **L** with respective ion have been carried out by gradual addition of micromolar concentration of those ions and the equation used to calculate the detection limit (DL)^1^ is DL = CL × CT

CL = Conc. of L; CT = Conc. of ion at which absorbance/ fluorescence enhanced.

5**. Separation of ions using L immobilized silica**

A methanol solution of the **L** (4 × 10^-2^ M) has been refluxed with silica (100-200 mesh) for 2h followed by slow evaporation of the solvent results coating of L over silica beads. The L coated silica beads are then air dried and used for experiment. A glass column (10 cm × 1 cm) is packed with L immobilized silica at a bed height of 2 cm. Then the solution of ion to be separated is passed through the column. Following the standard, protocol of washing etc., the sorbed ions are eluted with the solution composed of corresponding media and accurately transferred to 4 mL sample vial and absorption/ fluorescence intensity has been measured. Interestingly, the L immobilized silica show distinct colors with Fe^2+^/ Fe^3+^ ions in bare eye while with AsO_2_^-^ upon UV light irradiation.

**6. Real sample analysis**

In order to evaluate the feasibility of **L** towards determination of Fe^2+^ / Fe^3+^/ AsO_2_^-^, real samples have been analyzed. For that purpose, a known amount of mixture of iron salts (containing Fe^2+^ and Fe^3+^) is treated with **L** in the appropriate media and absorbance/ emission is measured at appropriate wavelength. From the calibration curve (absorbance/ emission vs. concentration), the concentration of the ions^2-3^ are determined.

7**. Hill equation**

Hill equation describes the degree of co-operatively of the L binding to the receptor. log[Y/(1-Y)] = nlog[G] + logK_app_, where Y, n, [G] and K_app_ represent the fraction of L binding sites filled, Hill co-efficient, concentration of guest and apparent association constant respectively. Y is determined by the equation, (I-I_0_)/ (I_max_ - I_0_), where I_0_, I, and I_max_ are the emission intensity and absorbance at corresponding wavelengths in absence, presence and at excess of corresponding ions.


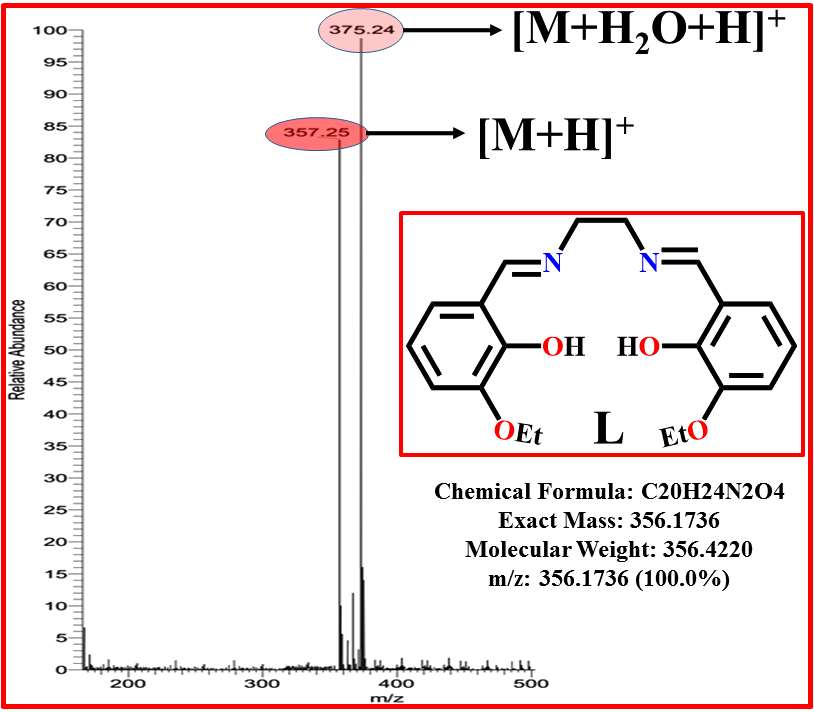


**Fig. S1** QTOF Mass spectrum of L in MeOH


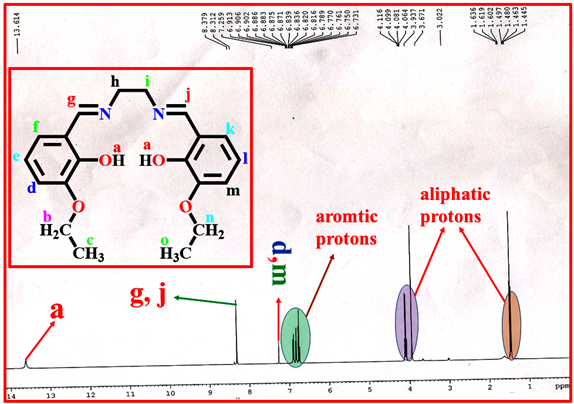


**Fig. S2a** ^1^HNMR spectrum of L in CDCl_3_


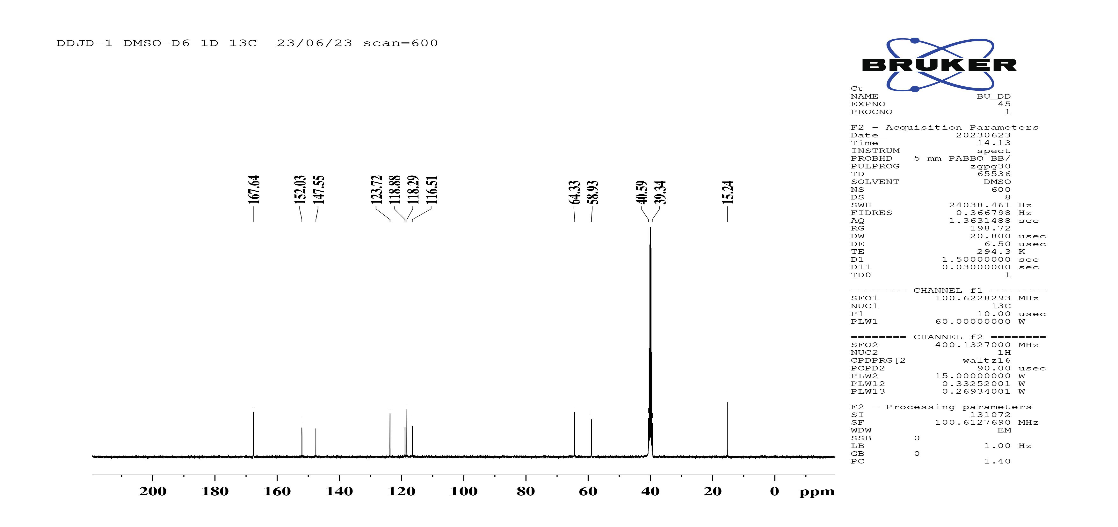


**Fig. S2b** ^13^C spectrum of L in DMSO-d_6_


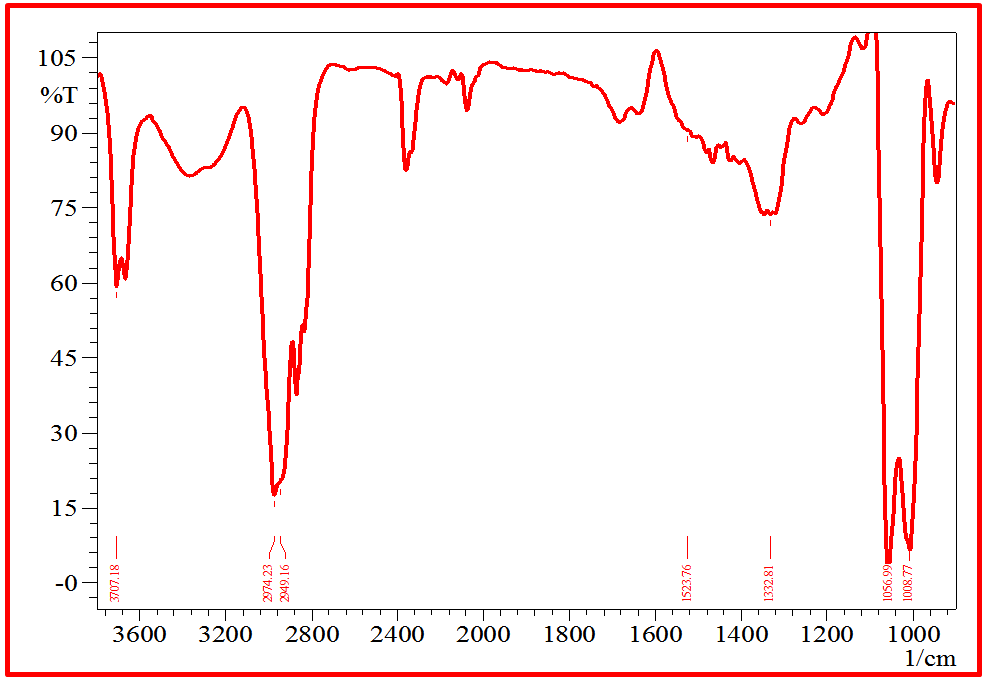


**Fig. S3** FTIR spectrum of L


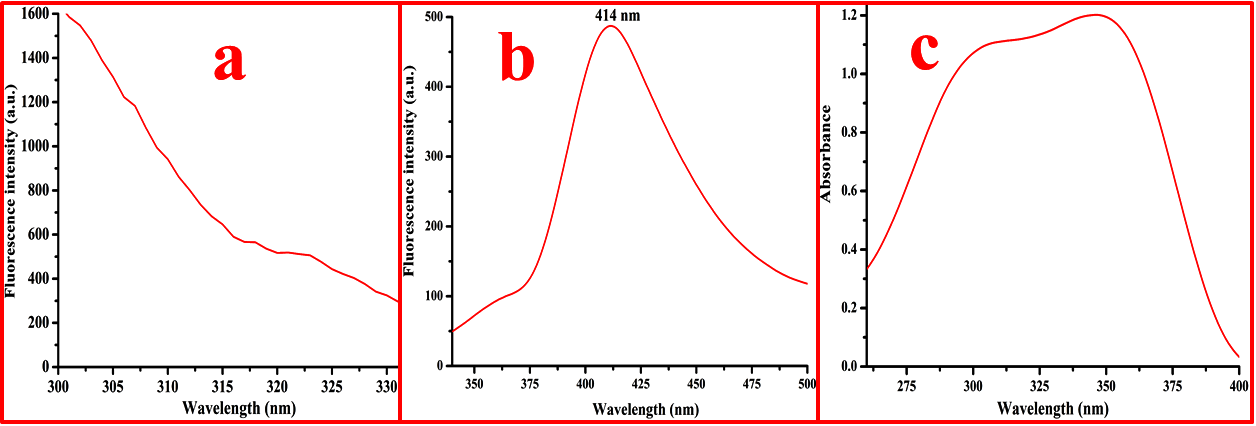


**Fig. S4** (a) Excitation, (b) emission and (c) absorption spectra of L (MeOH/H_2_O, 4/1, v/v, pH 7.4)


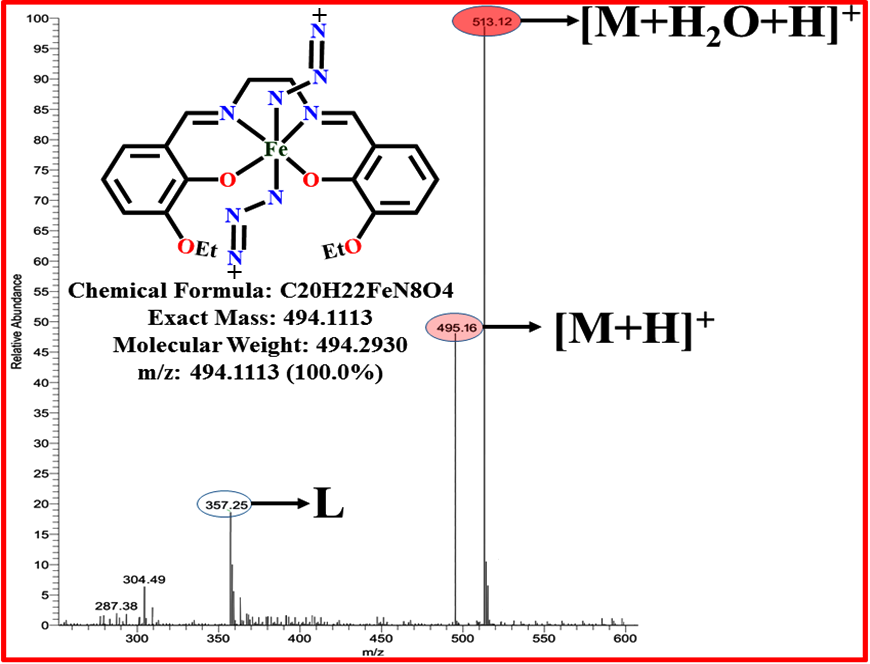


**Fig. S5** QTOF-MS spectrum of [L-Fe^2+^] complex in MeOH


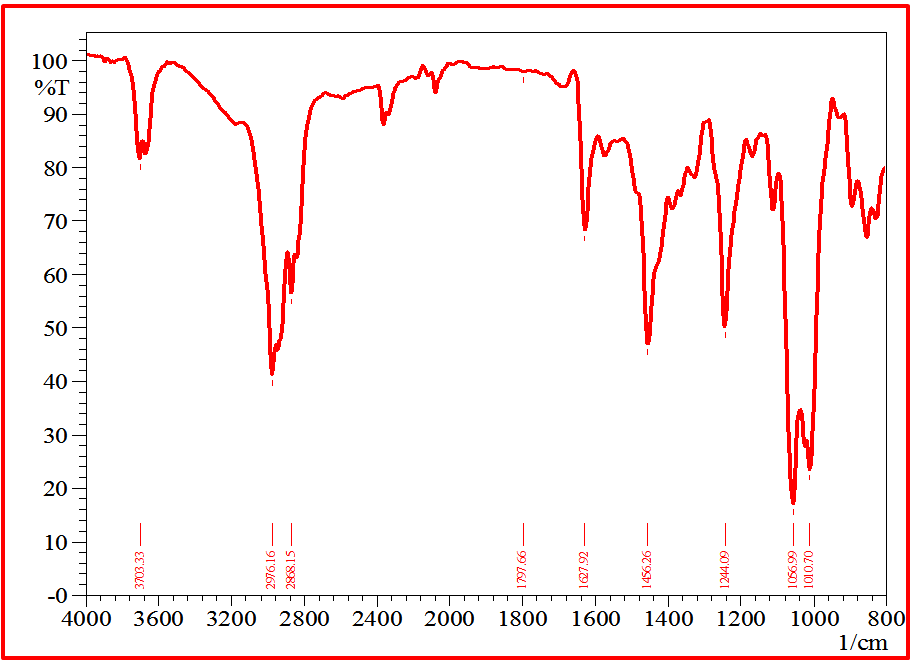


**Fig. S6** FTIR spectrum of [L-Fe^2+^] complex


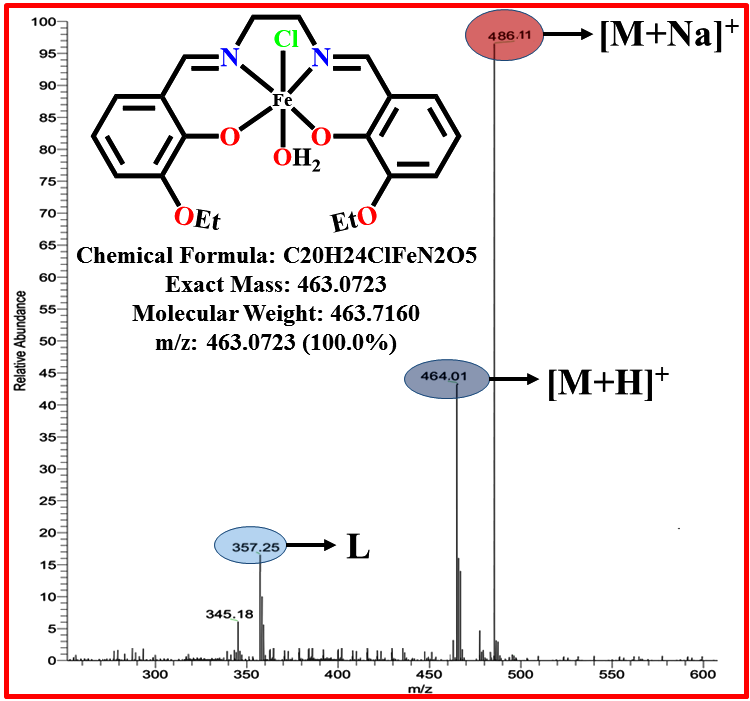


**Fig. S7** QTOF-MS spectrum of [L-Fe^3+^] complex in MeOH


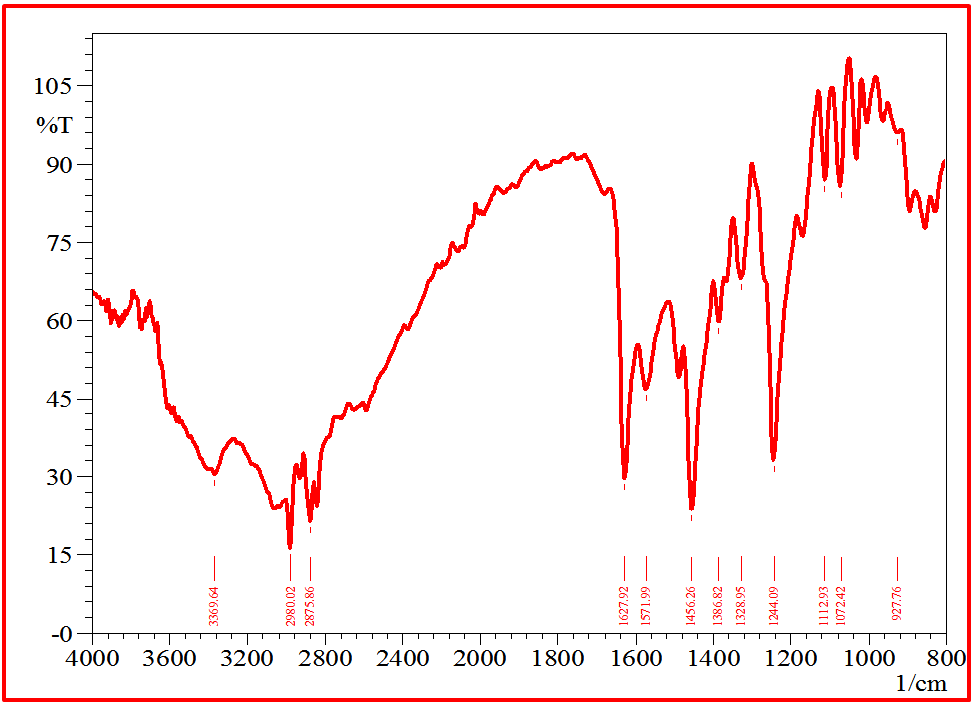


**Fig. S8** FTIR spectrum of [L-Fe^3+^]


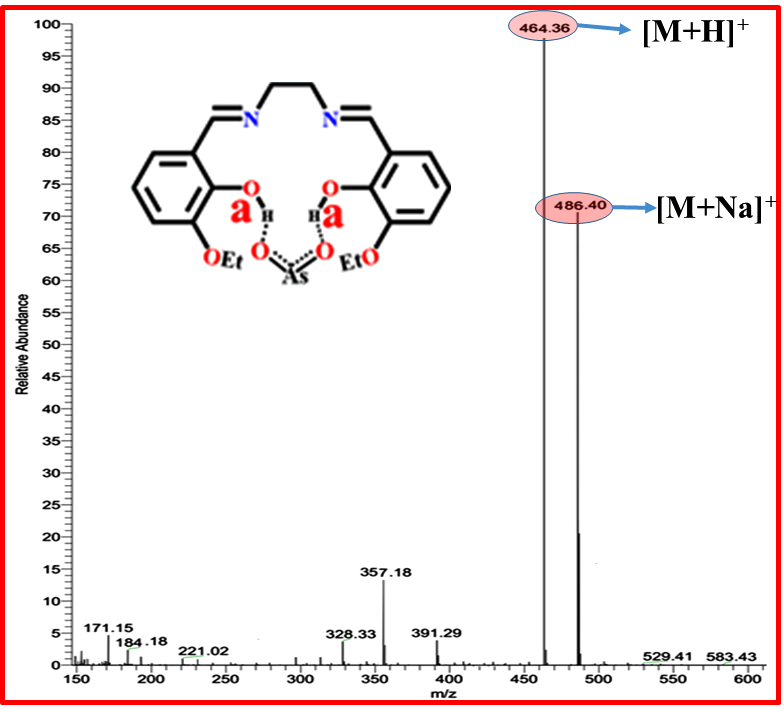


**Fig. S9** QTOF mass spectrum of [L-AsO_2_^-^] in MeOH


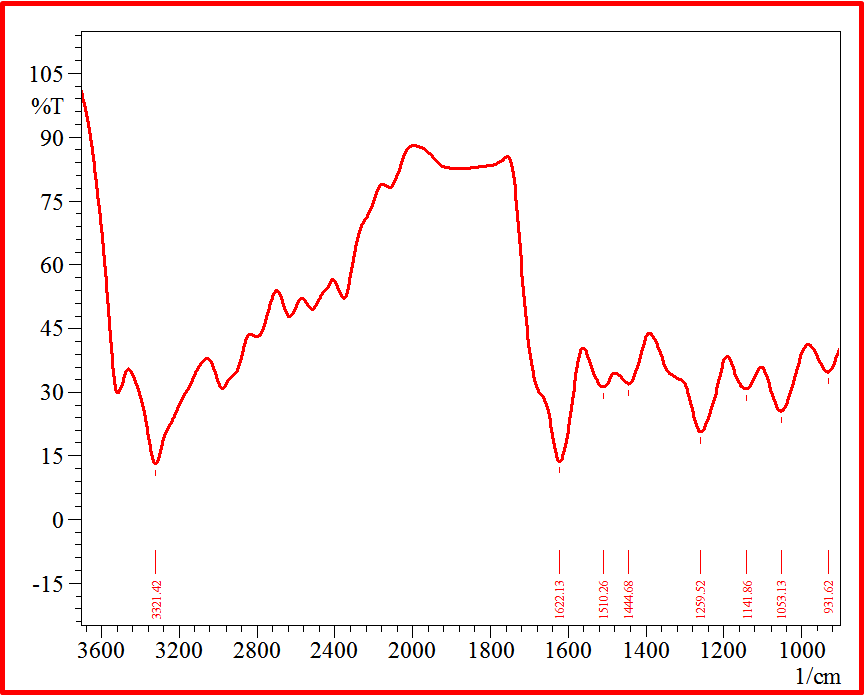


**Fig. S10** FTIR spectrum of [L-AsO_2_^-^]


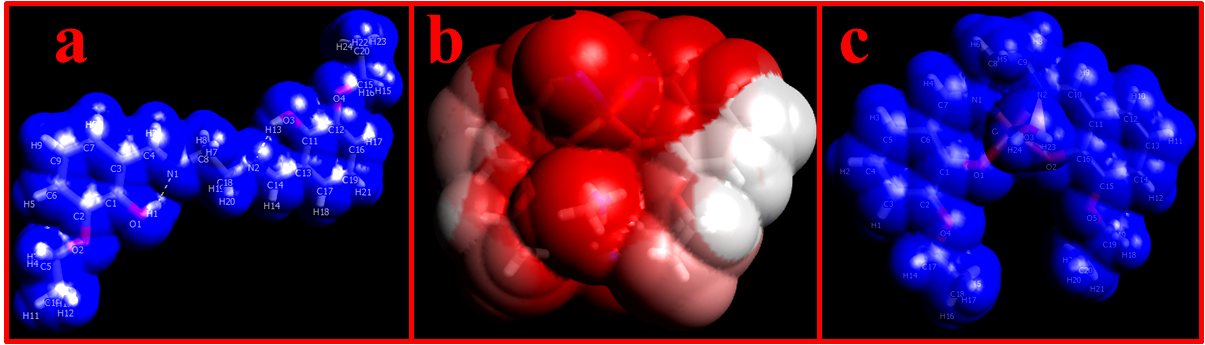


**Fig. S11** View of 3D- connectivity in terms of van der Waals charge distribution with different layers in (a) L (b) [Fe(L)(N_3_)_2_(NH_3_CH_2_CH_2_NH_3_)] and (C) [Fe(L)(Cl) (H_2_O)]


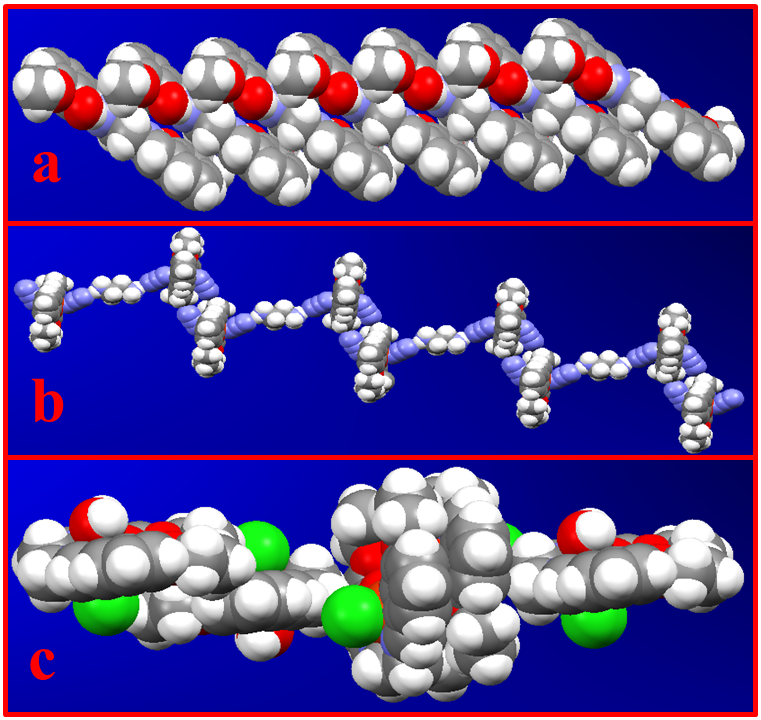


**Fig. S12** A space-fill view of (a) L; (b) [L-Fe^2+^] complex and (c)[L-Fe^3+^] complex

| 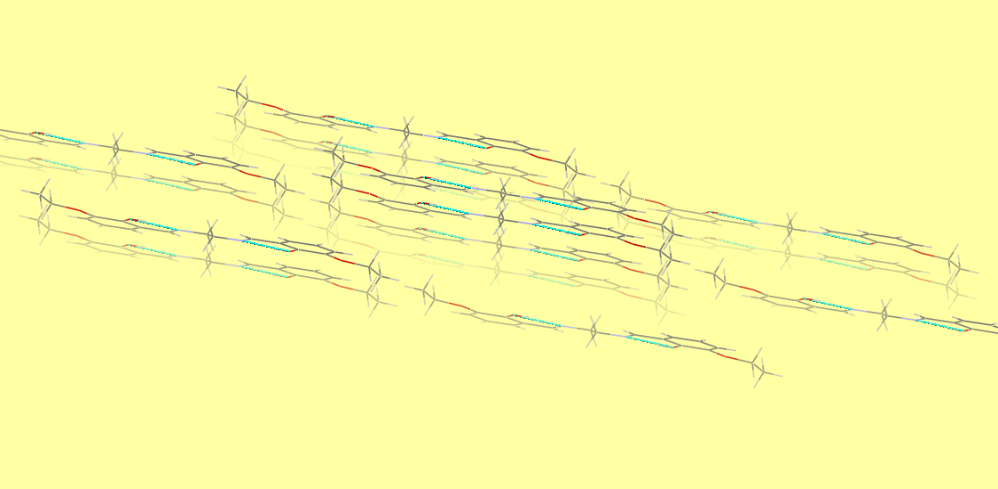  **a** | |  |
| --- | --- | --- |
| 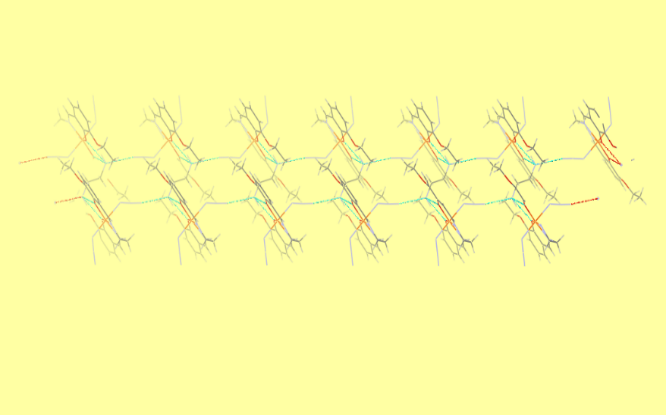  **b** | 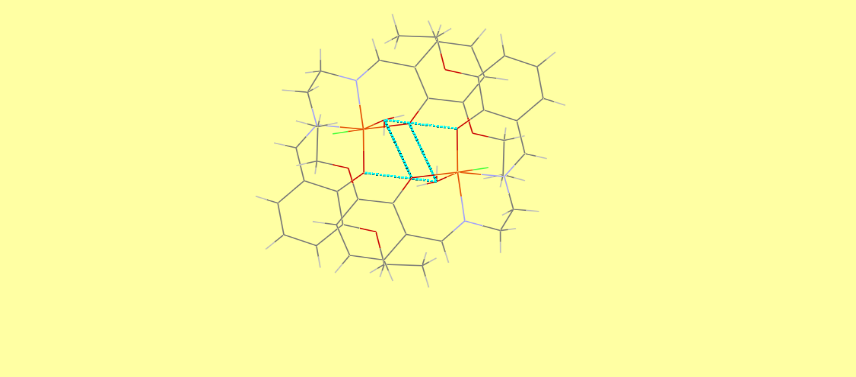  **c** | |

**Fig. S13** Crystal structure as 1-D network of (a) (L) showing intra-molecular O-H···N bonds as sky dashed lines. The inter-molecular potential is depicted in white dashed lines; (b) [L-Fe^2+^] complex showing inter-molecular hydrogen bonds as sky dashed lines; (c) [L-Fe^3+^] complex showing inter-molecular hydrogen bonds as sky dashed lines.


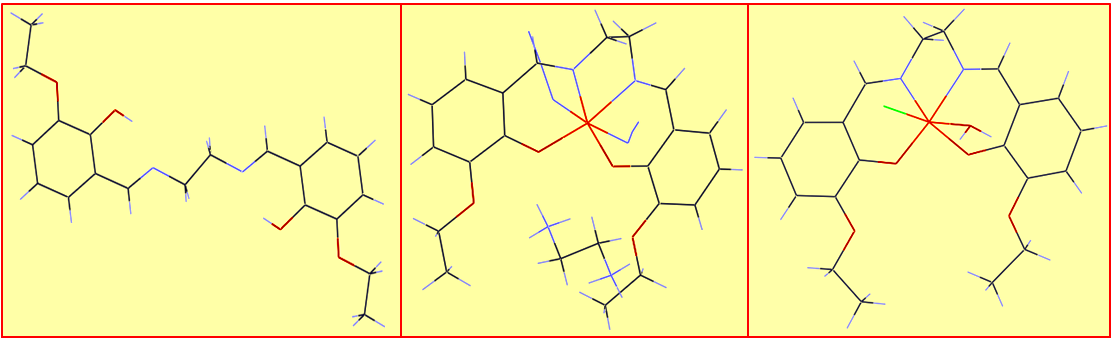


**Fig. S14** 3D view of crystal structures in wireframe model of (a) L; (b) ([L-Fe^2+^] and (c) ([L-Fe^3+^] complex


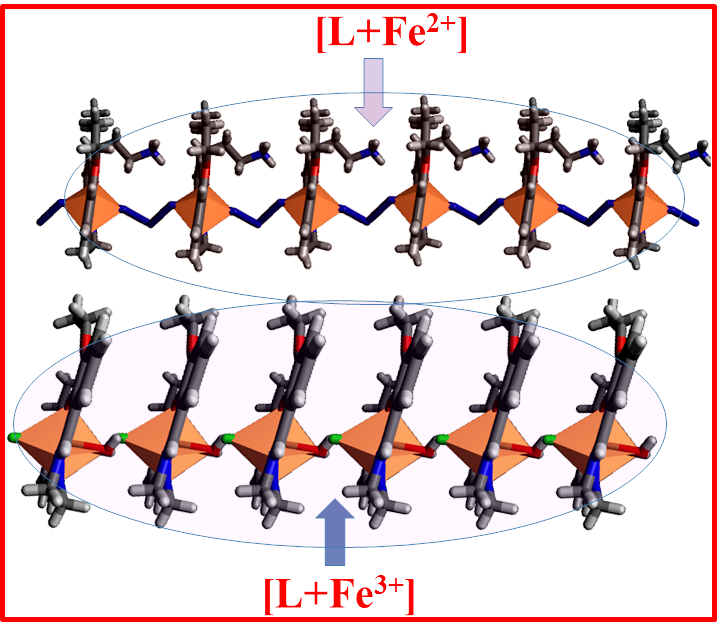


**Fig. S15** Polygon (3D framework) view of [L-Fe^2+^] and[L-Fe^3+^] complexes


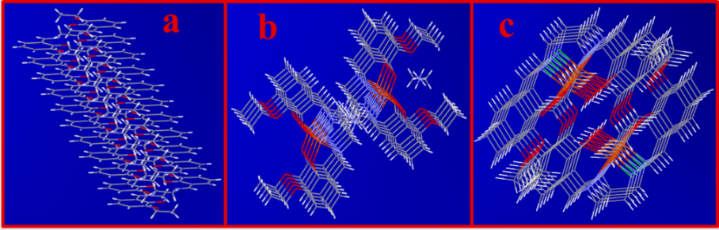


**Fig.S16 3D** view of crystal packing of (a) L in wireframes model down c-axis, pointing towards channels; (b) [L-Fe^2+^] complex and (C) [L-Fe^3+^] complex


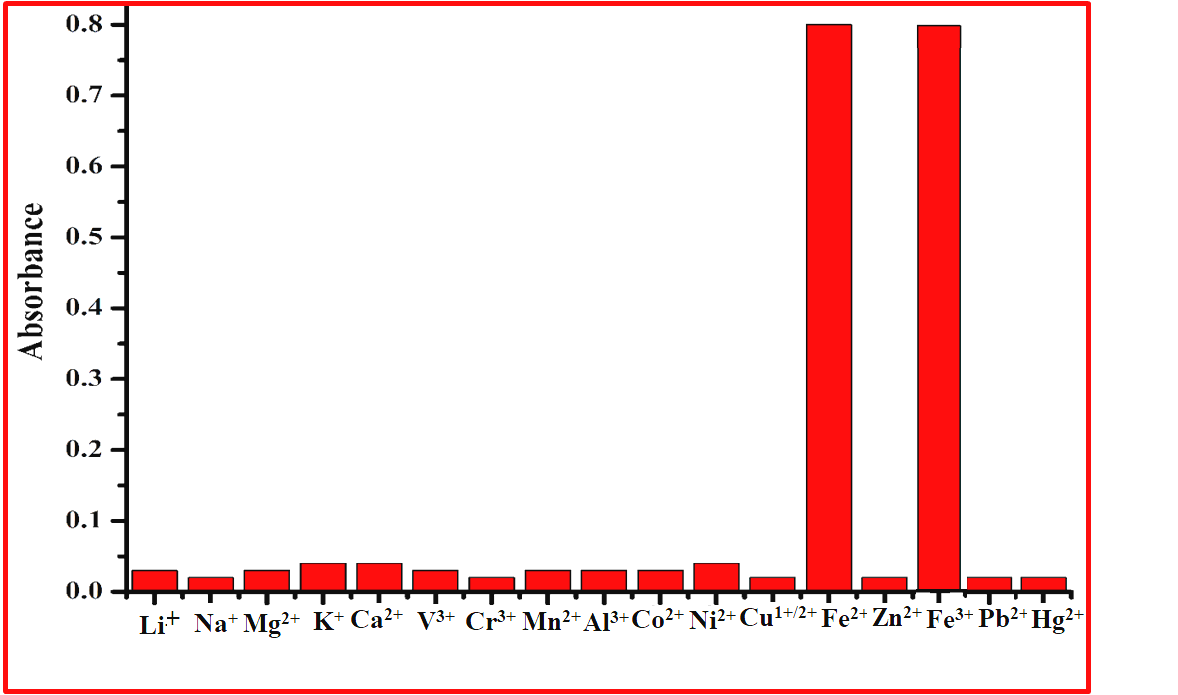


**Fig. S17** Absorbance of L in presence of Fe^2+^ and Fe^3+^with other metal ions (Li^+^, Na^+^, K^+^, Ca^2+^, Mg^2+^, V^3+^, Mn^2+^, Ni^2+^, Cr^3+^, Fe^3+^, Fe^2+^, Cu^+^/^2+^, Co^2+^, Zn^2+^, Pb^2+^ and Hg^2+^), λ_max_ = 538 nm for Fe^2+^, λ_max_ = 606 nm for Fe^3+^, media: MeOH/H_2_O (4/1, v/v, pH 7.4)


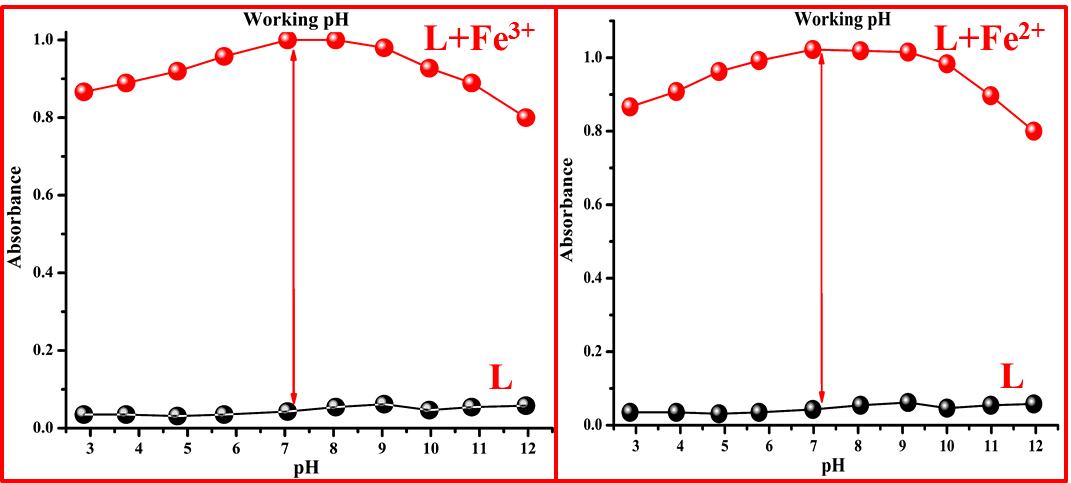


**Fig. S18** Effect of pH on the absorbance of free L (20 μM) and its Fe-complexes (MeOH/H_2_O, 4/1, v/v, pH 7.4)


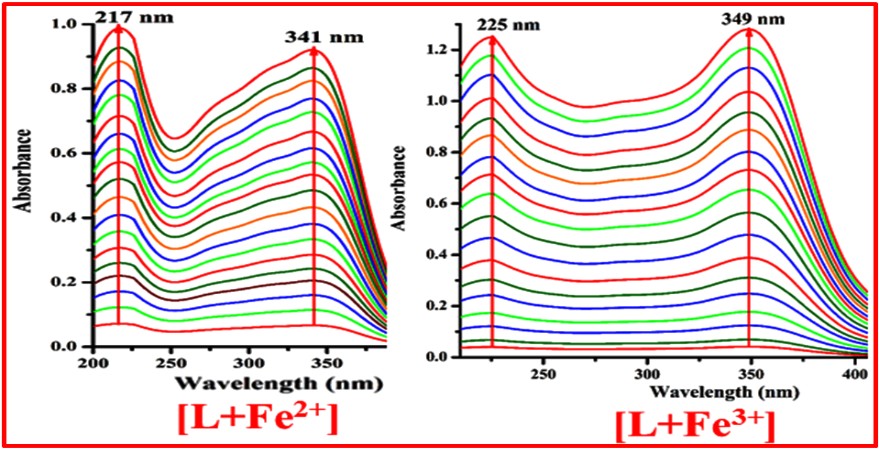


**Fig. S19** Changes in the absorption spectra of L (20 μM) in 20 mM HEPES-buffered MeOH/H_2_O (4/1, v/v, pH 7.4) upon gradual addition of Fe^2+/3+^ ions (μM)


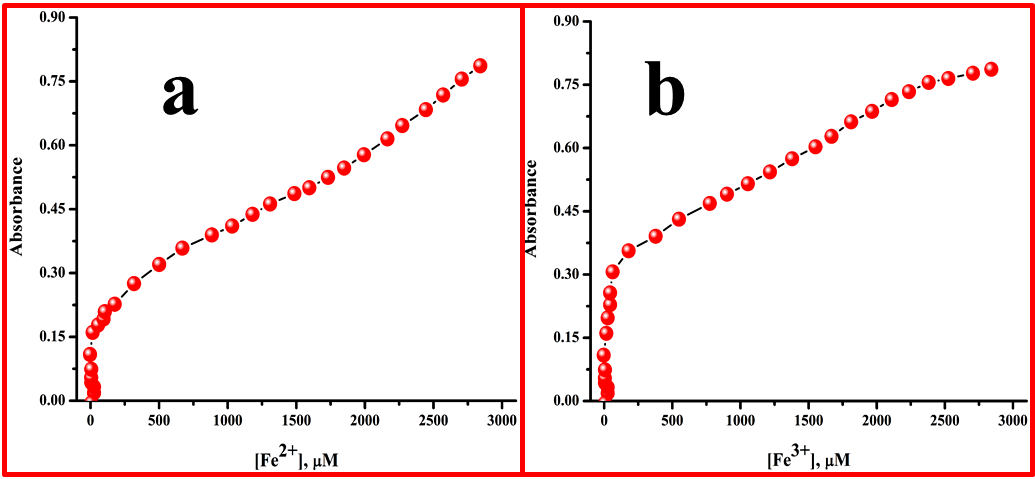


**Fig. S20** Plots of absorbance of L (20 μM) as a function of externally added (a) Fe^2+^ and (b) Fe^3+^ (0-3000 μM) (MeOH/H_2_O, 4/1, v/v, pH 7.4)


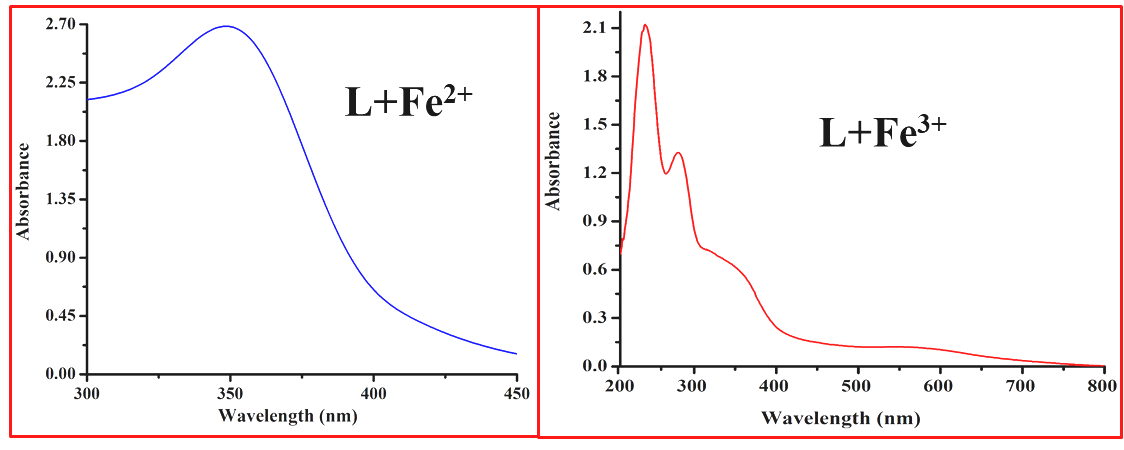


**Fig. S21** Plots of absorbance of L (20 μM) in presence Fe^2+^and Fe^3+^(MeOH/H_2_O, 4/1, v/v, pH 7.4)


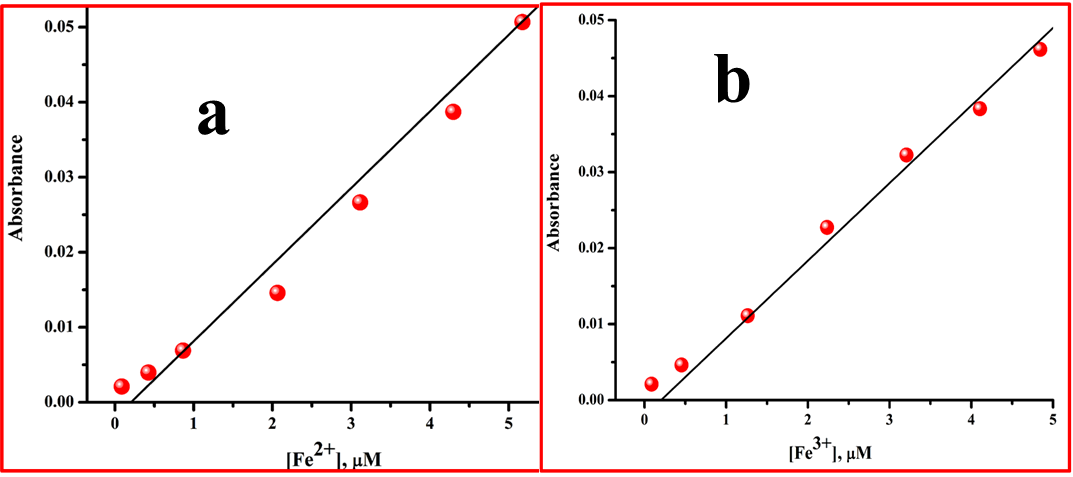


**Fig. S22** Liner region of the plot of concentration vs. absorbance for (a) Fe^2+^ and (b) Fe^3+^ (MeOH/H_2_O, 4/1, v/v, pH 7.4)


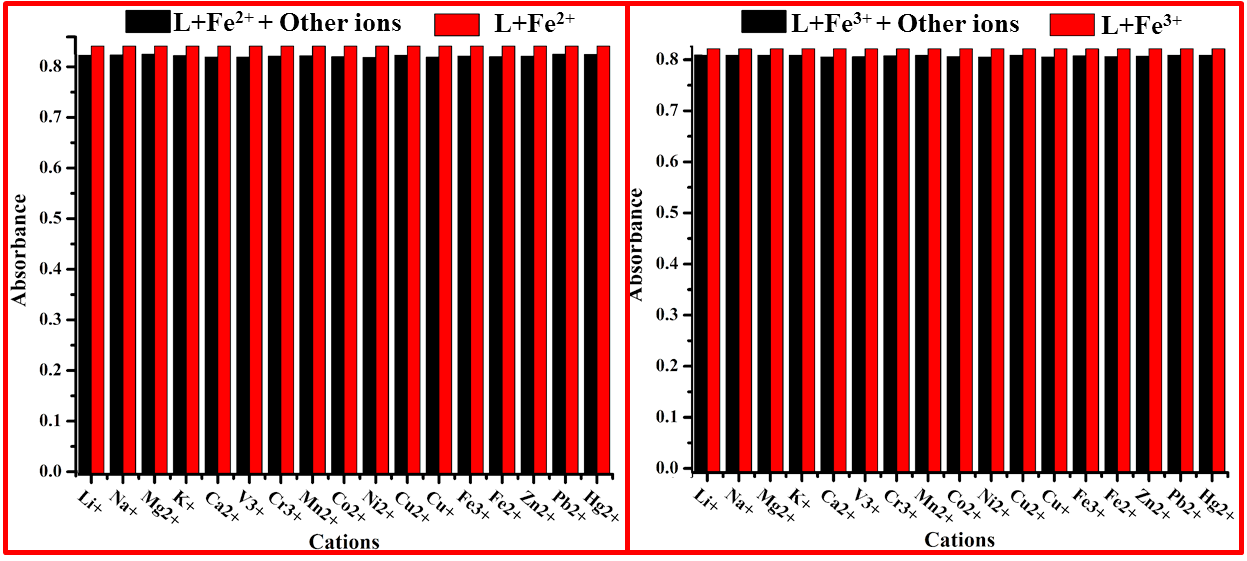


**Fig. S23** Absorbance of L (20 µM) in presence of different cations (100 µM): Li^+^, Na^+^, K^+^, Ca^2+^, Mg^2+^, V^3+^, Mn^2+^, Ni^2+^, Cr^3+^, Fe^3+^, Fe^2+^, Cu^2+^, Cu^+^, Co^2+^, Zn^2+^, Pb^2+^ and Hg^2+^ in 20 mM HEPES buffered MeOH/H_2_O (4/1, v/v, pH 7.4), (λ= 538 nm (for Fe^2+^), λ = 606 nm) (for Fe^3+^)


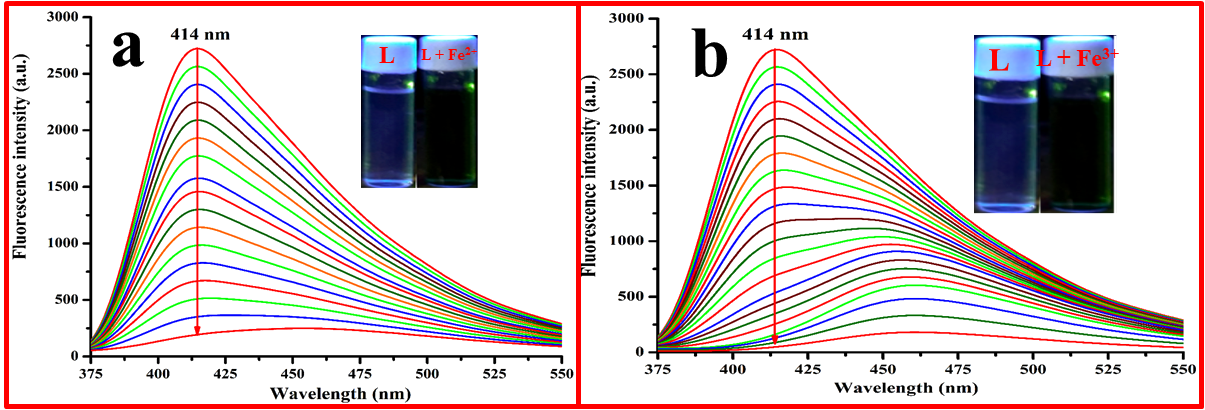


**Fig. S24** Emission spectra of L (20 μM) in 20 mM HEPES-buffered MeOH/H_2_O (4/1, v/v, pH 7.4) upon gradual addition (0.0, 0.05, 0.1, 0.5, 1.0, 5.0, 10, 20, 50, 75, 100, 200, 300, 500, 1000, 1200, 1600, 2000 and 3000 μM) of (a) Fe^2+^ (λ_ex_ = 322 nm) and (b) Fe^3+^ (λ_ex_ = 322 nm)


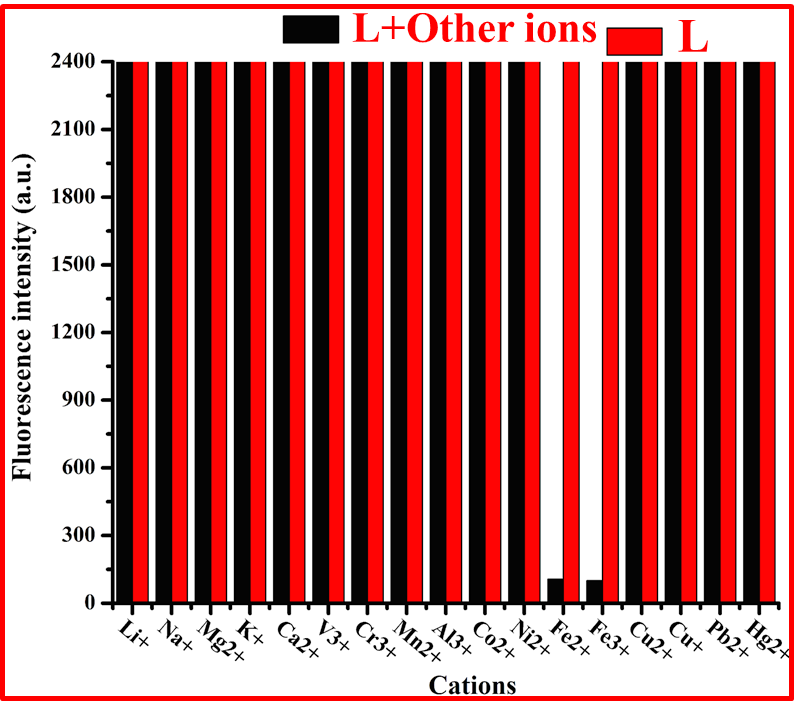


**Fig. S25** Emission intensity of L (20 µM) at 414 nm in presence of different cations (100 µM): Li^+^, Na^+^, K^+^, Ca^2+^, Mg^2+^, V^3+^, Mn^2+^, Ni^2+^, Cr^3+^, Fe^3+^, Cu^2+^, Cu^+^, Co^2+^, Al^3+^, Zn^2+^, Pb^2+^ and Hg^2+^ in 20 mM HEPES buffered MeOH/H_2_O (4/1, v/v, pH 7.4), (λ_ex_ = 322 nm, λ_em_ = 414 nm)


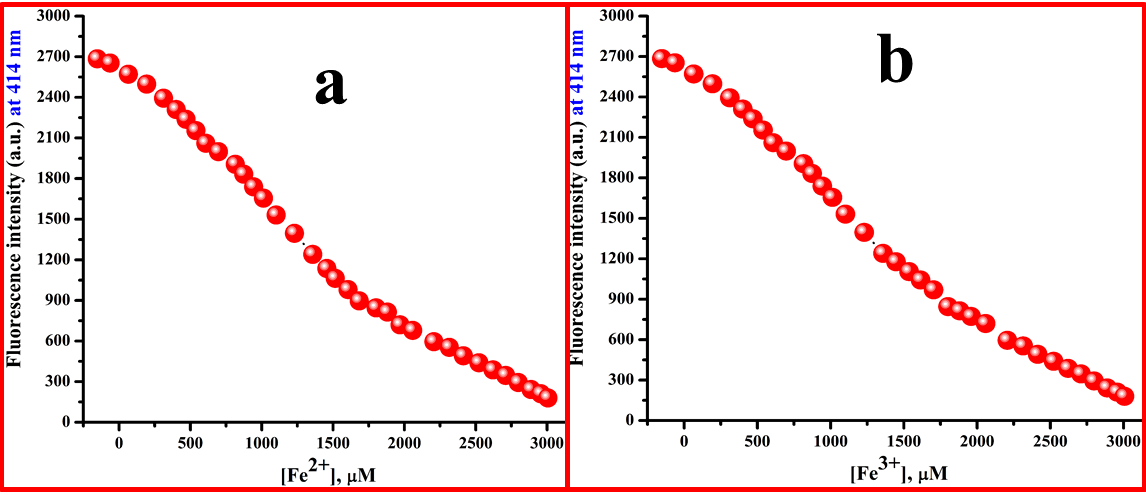


**Fig. S26** Plot of emission intensities of L (20 μM, MeOH/H_2_O, 4/1, v/v, 20 mM HEPES buffer, pH 7.4), λ_ex_= 322 nm, λ_em_ = 414 nm) as a function of externally added (a) Fe^2+^ (0-3000 μM), (b) Fe^3+^ (0-3000 μM)


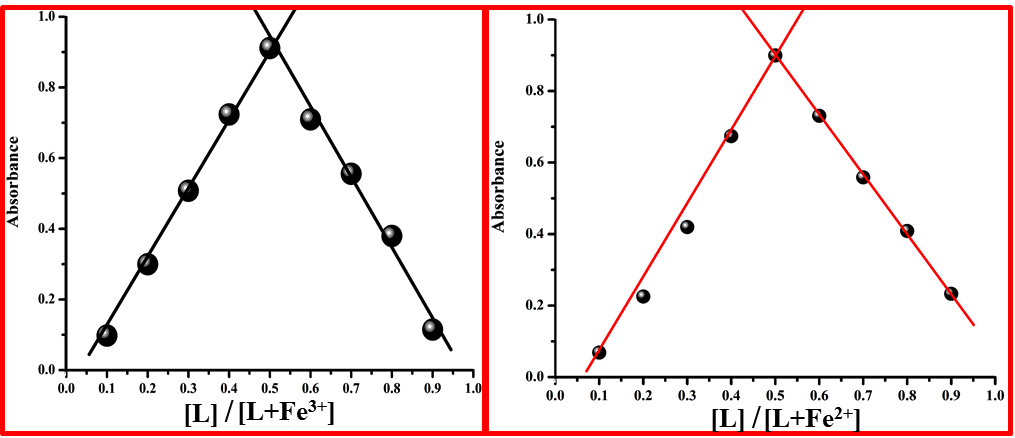


**Fig. S27** Job’s plot for determination of stoichiometry of the [L- Fe^2+^] and [L- Fe^3+^] complex (MeOH/H_2_O, 4/1, v/v, pH 7.4)


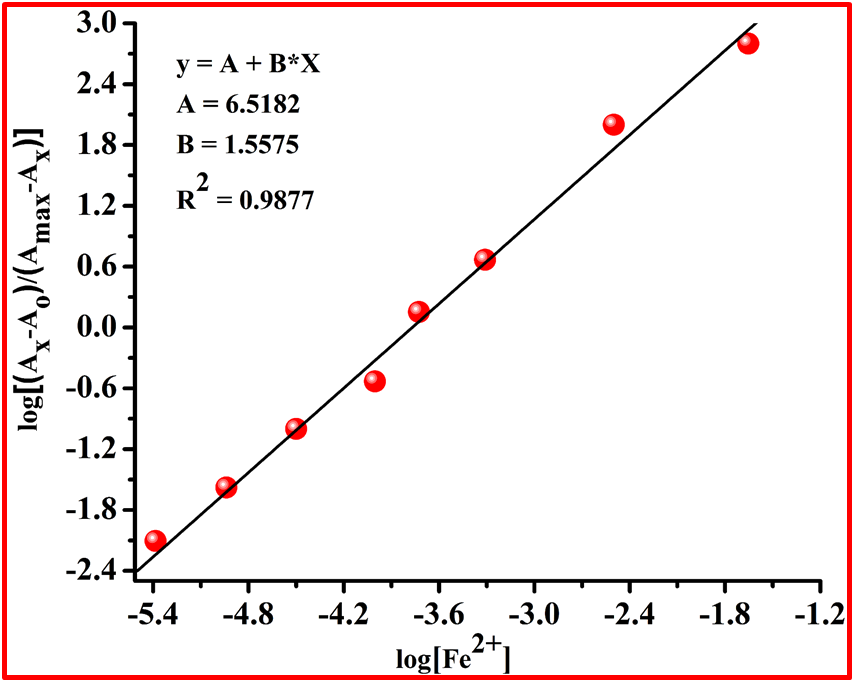


**Fig. S28** Hill plot for determination of binding constant of L for Fe^2+^ in MeOH/H_2_O (4/1, v/v, pH 7.4)


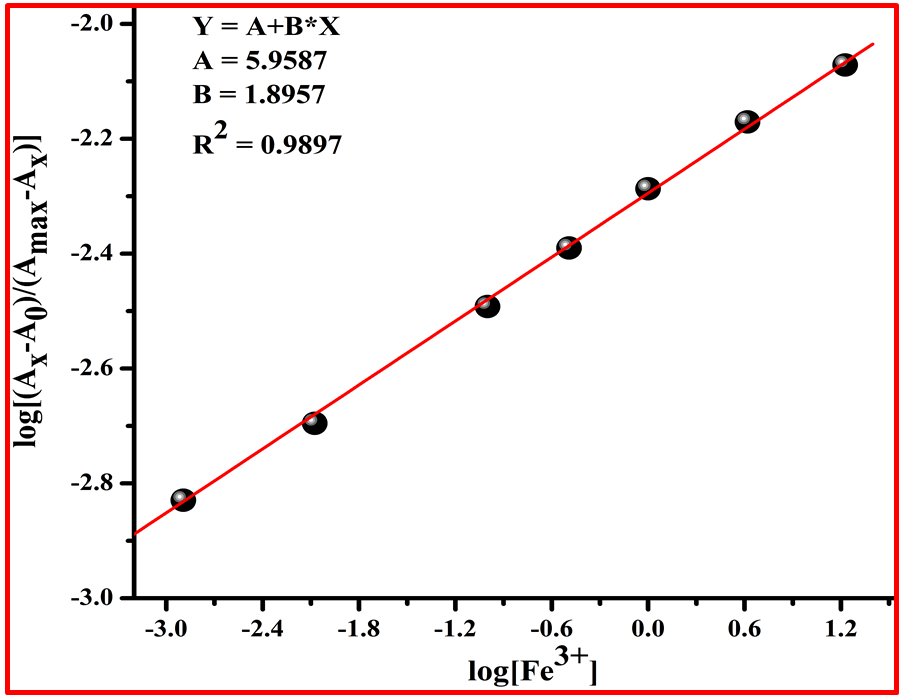


**Fig. S29** Hill plot for determination of binding constant of Lfor Fe^3+^ in MeOH/H_2_O (4/1, v/v, pH 7.4)


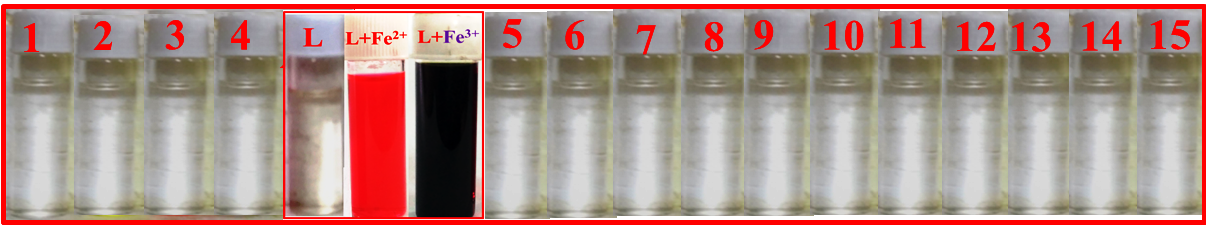


**Fig. S30** The colors of L in bare eye after addition of common metal ions (1 = Li^+^, 2 = Na^+^, 3 = Mg^2+^, 4 = K^+^, 5 = Ca^2+^, 6 = Al^3+^, 7 = V^3+^, 8 = Cr^3+^, 9 = Mn^2+^, 10 = Co^2+^, 11 = Ni^2+^, 12 = Cu^2+^, 13 = Hg^2+^, 14 = Zn^2+^ and Pb^2+^


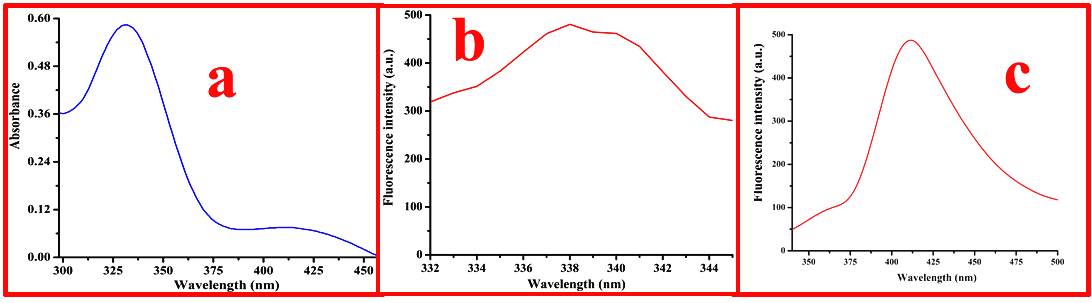


**Fig. S31** (a) Absorption, (b) excitation and (c) emission spectra of L in DMSO/H_2_O (4/1, v/v, pH 7.4)


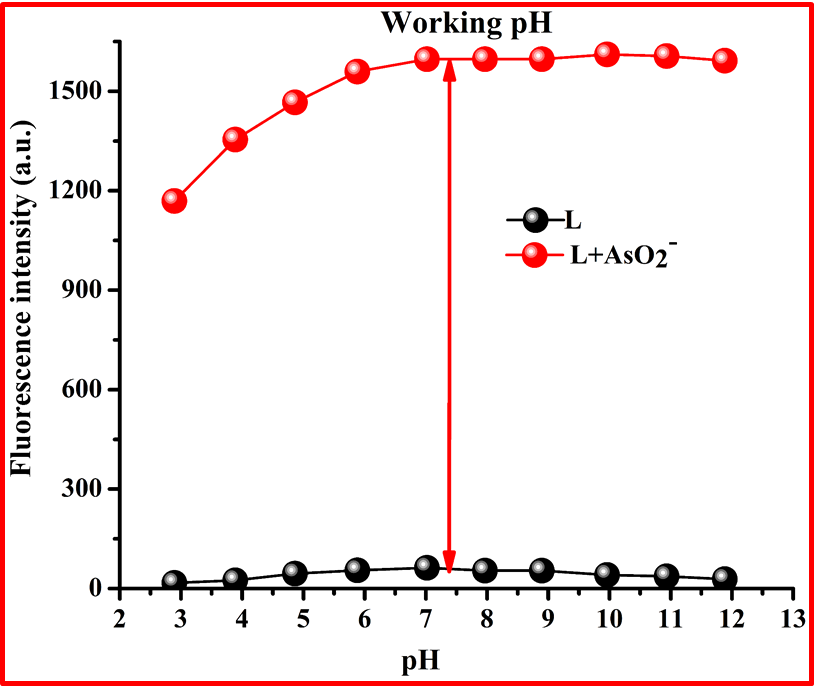


**Fig. S32** Effect of pH on the emission intensities of free L (20 μM) and its [L-AsO_2_^-^] adduct in DMSO/H_2_O, 4/1, v/v


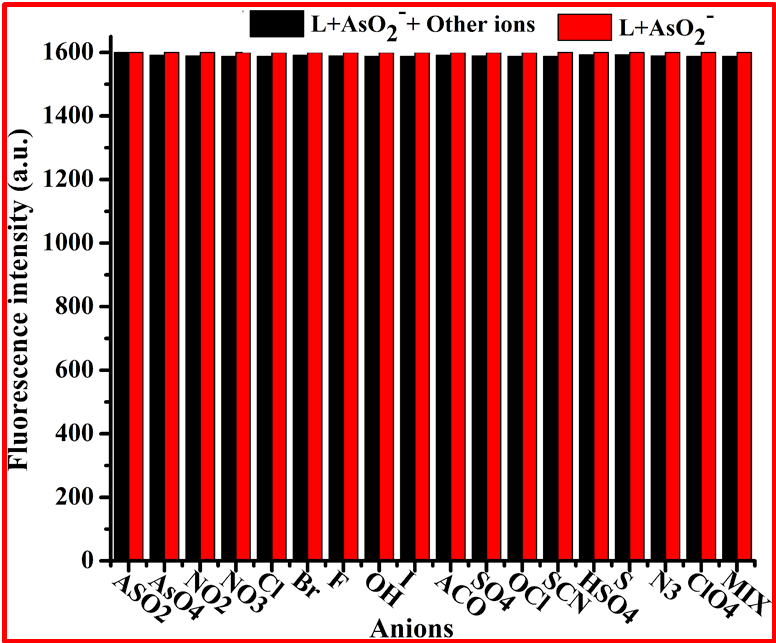


**Fig. S33** Emission intensity of L (20 µM) at 450 nm in presence of different anions (100 µM): AsO_2_^-^, AsO_4_^3-^, NO_2_^-^, NO_3_^-^, Cl^-^, Br^-^, F^-^, OH^-^, I^-^, AcO^-^, SO_4_^2-^, HSO_4_^-^, OCl^-^, SCN^-^, S^2-^, N_3_^-^, ClO_4_^-^ and mix in 20 mM HEPES buffered DMSO/H_2_O (4/1, v/v, pH 7.4), (λ_ex_ = 338 nm, λ_em_ = 450 nm)


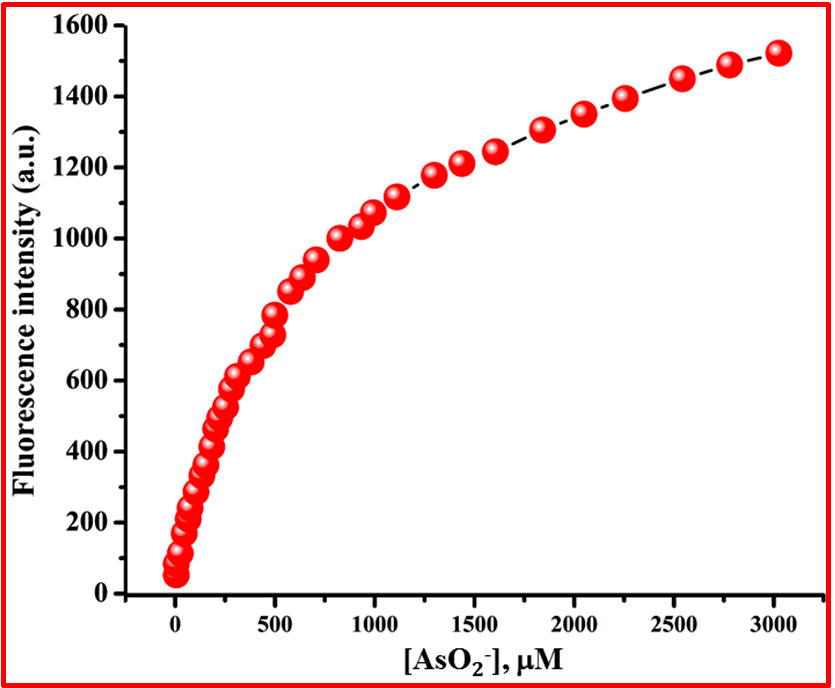


**Fig. S34** Plot of emission intensities of L(20 μM; DMSO/H_2_O, 4/1, v/v; HEPES buffer, pH 7.4) as a function of externally added AsO_2_^-^ (0.005-3000 μM)


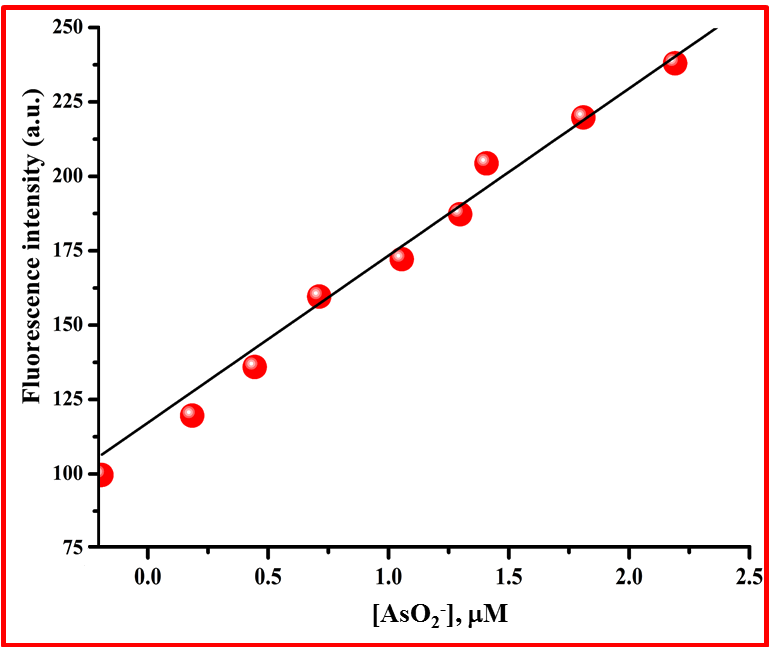


**Fig. S35** Plot of emission intensities of L(20 μM; DMSO/H_2_O, 4/1, v/v; HEPES buffer, pH 7.4) as a function of externally added AsO_2_^-^ (0.005-2.5 μM)


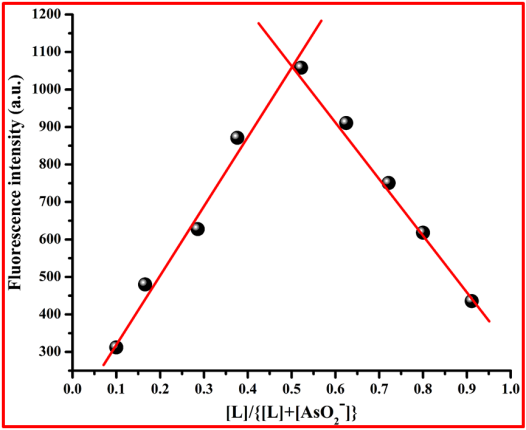


**Fig. S36**Job’s plot for determination of stoichiometry of the [L-AsO_2_^-^] adduct (λ_ex_ = 338 nm, λ_em_ = 450 nm) (DMSO/H_2_O, 4/1, v/v, pH 7.4)


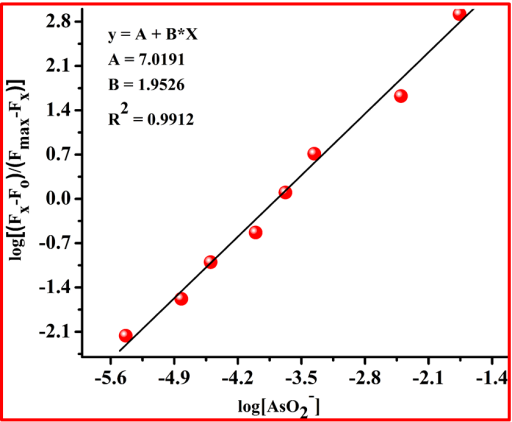


**Fig. S37** Hill plot for determination of binding constant of L for AsO_2_^-^ in DMSO/H_2_O (4/1, v/v, pH 7.4)


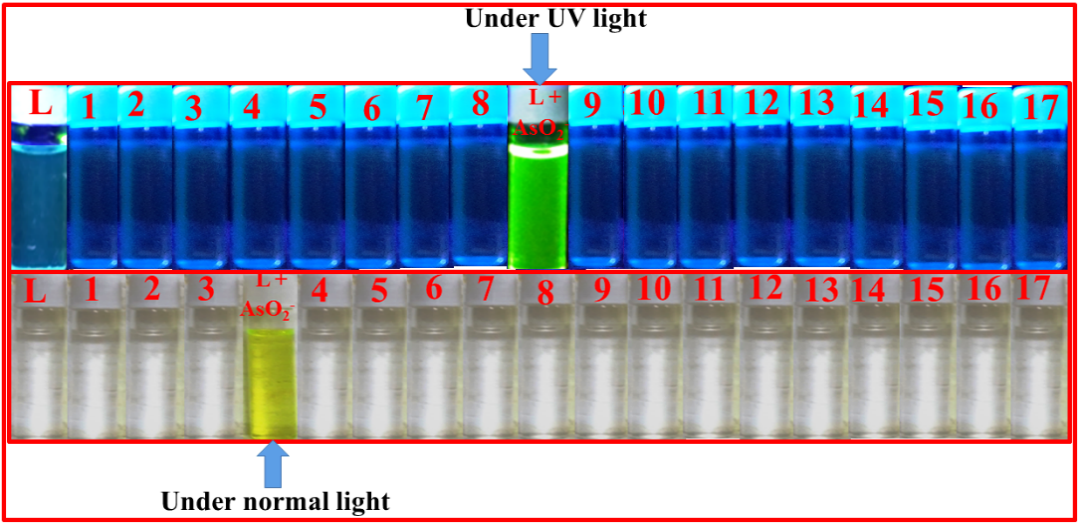


**Fig. S38** Changes in color of L under (a) UV and (b) visible light upon addition of common anions (20 µM, DMSO/H_2_O, 4/1, v/v, pH 7.4) ( 1= AsO_4_^3-^, 2 = NO_2_^-^, 3 = NO_3_^-^, 4 = Cl^-^, 5 = Br^-^, 6 = F^-^, 7 = OH^-^, 8 = I^-^, 9 = AcO^-^, 10 = SO_4_^2-^, 11 = HSO_4_^-^, 12 = OCl^-^, 13 = H_2_PO_4_^-^, 14 = SCN^-^, 15 = S^2-^, 16 = N_3_^-^, 17 = ClO_4_^-^)


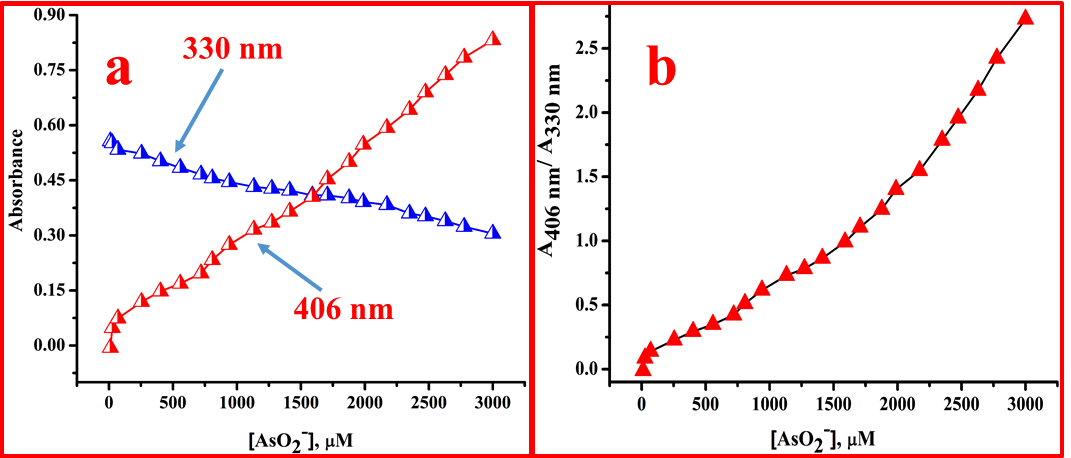


**Fig. S39**  (a) Plot of absorbance of L (λ, 330 nm and 406 nm) as a function of added AsO_2_^-^ (0-3000 μM); (b) corresponding ratiometric change (DMSO/H_2_O, 4/1, v/v, pH 7.4)

**Synthesis of L1**

The probe, L1 has been synthesized by refluxing the mixture of benzaldehyde, (0.50 g, 2.11 mmol) and ethylenediamine (0.06 g, 1.05 mmol) in methanol for 6h at 60^0^C (Scheme S1). The yellow compound is obtained after slow evaporation of the solvent. Anal. calcd (%): C, 77.00; H, 9.01 and N, 10.88; found: C, 77.01; H, 9.10 and N, 10.81. QTOF–MS ES^+^ (Fig.S39, ESI) m/z calcd. for C_16_H_16_N_2_: 236.13, found: 237.19 ([M+H] ^+^), 259.20 ([M+ Na] ^+^). FTIR (cm^−1^) (Fig.S40, ESI): υ (C-H, aromatic) 2981, 2879, υ (CH=N, imine bond) 1664, υ (C=C, stretch) 1452, υ (C-N, stretch) 1240.





**Scheme S1** Synthetic protocols (**a** = MeOH, Reflux, 60°C)


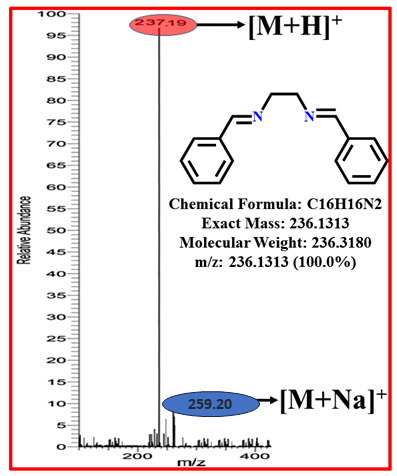


**Fig. S40** QTOF Mass spectrum of L1 in MeOH


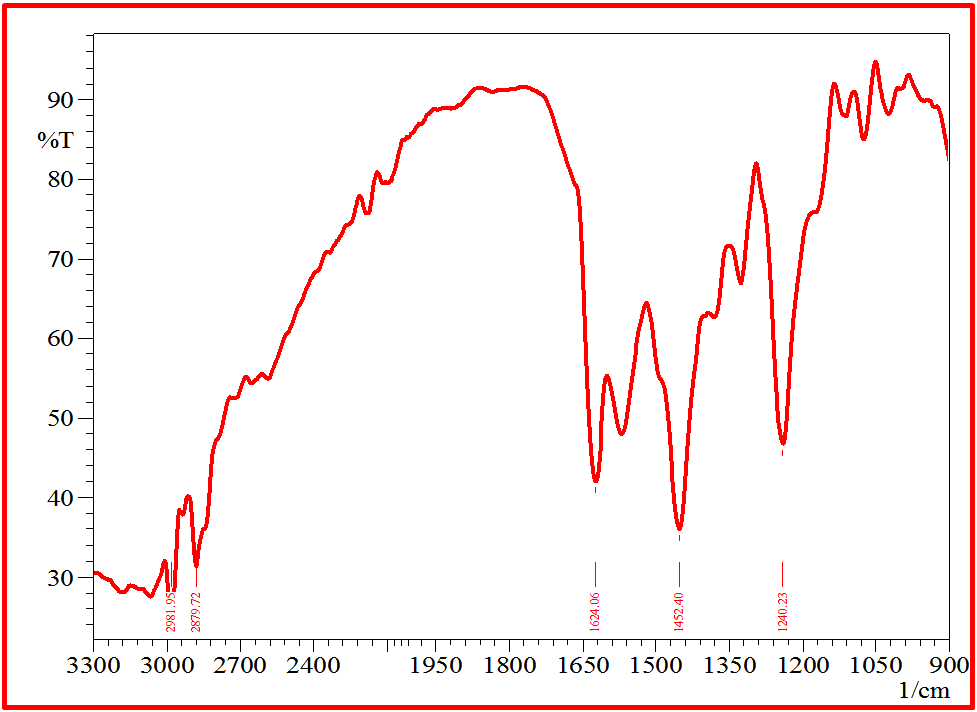


**Fig. S41**FTIR spectrum of Ll


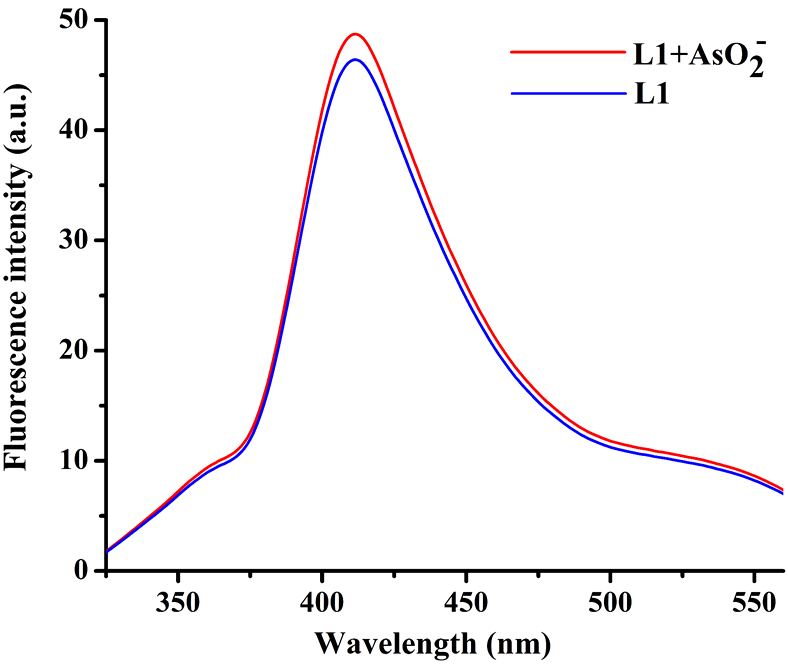


**Fig. S42** Changes in emission spectra of L1(20 μM) in HEPES-buffered (20 mM, DMSO/ H_2_O, 4/1, v/v, pH 7.4) solution upon addition of AsO_2_^-^ (20 μM)


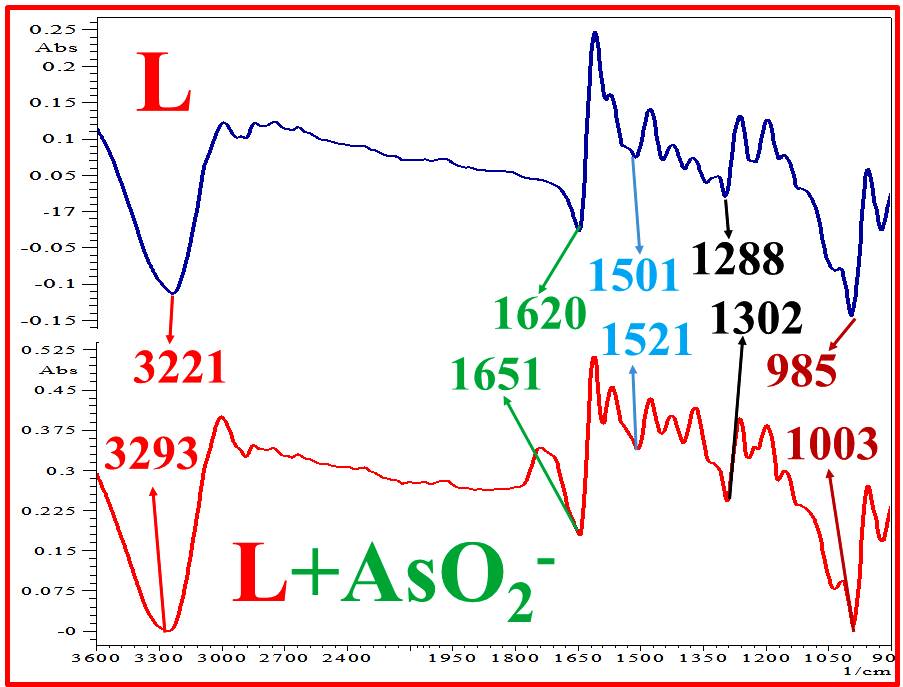


**Fig. S43** Overlay of FTIR spectra of L and [L- AsO_2_^-^] adduct


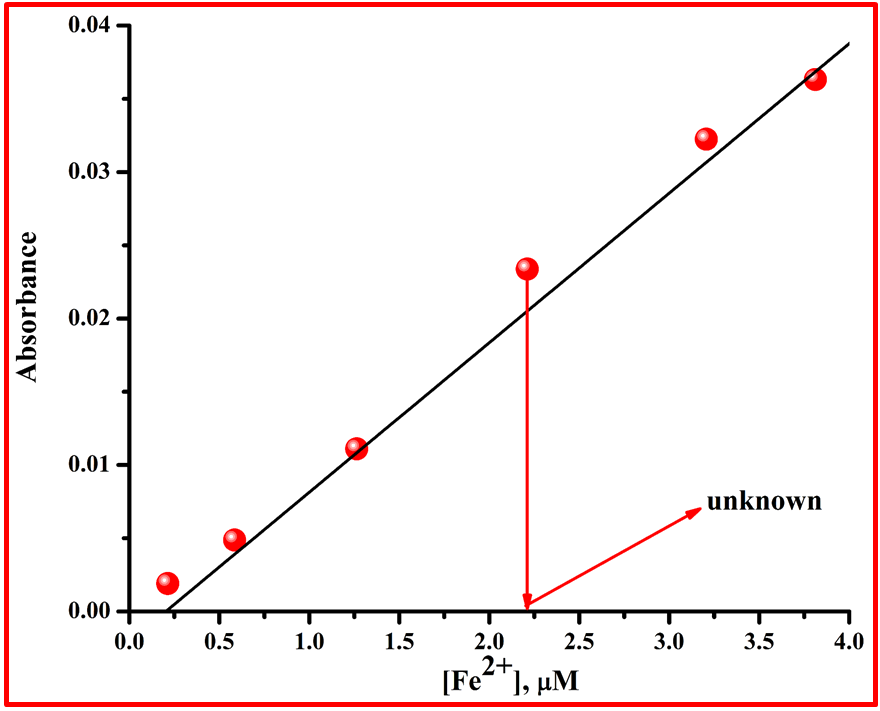


**Fig. S44** Calibration graph for estimation of Fe^2+^


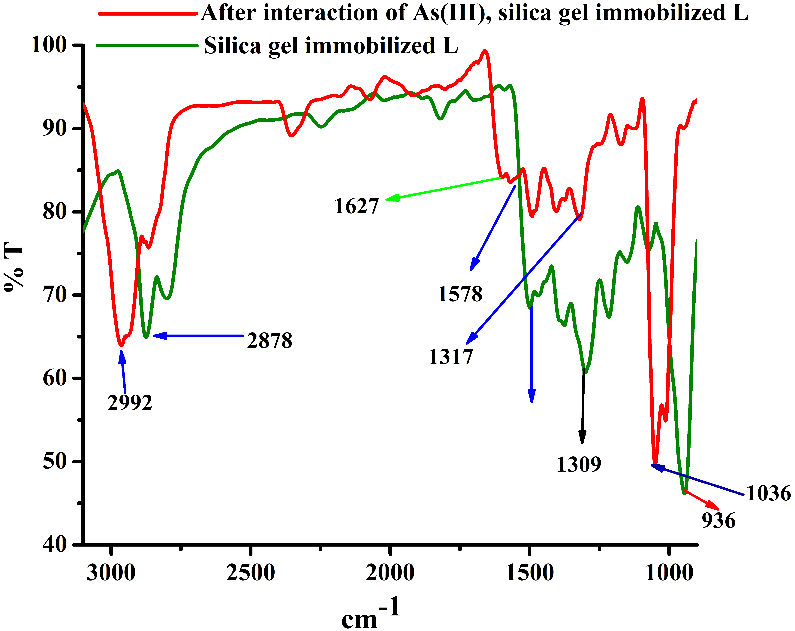


**Fig. S45** FTIR spectra of L immobilized silica (green) and its AsO_2_^-^sorbed form (red)


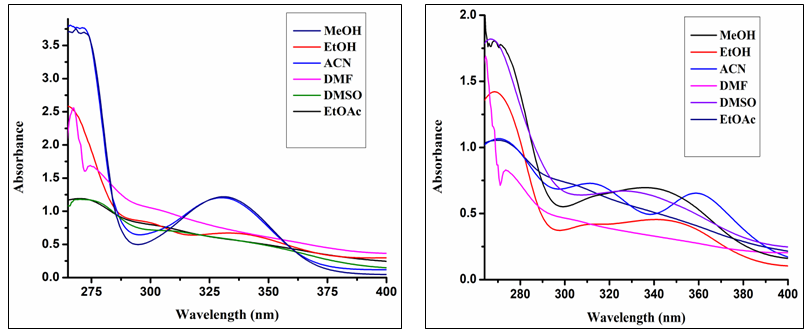


**Fig. S46** Effect of solvent on the absorption spectra of L in presence of Fe^2+^(left) and Fe^3+^(right)


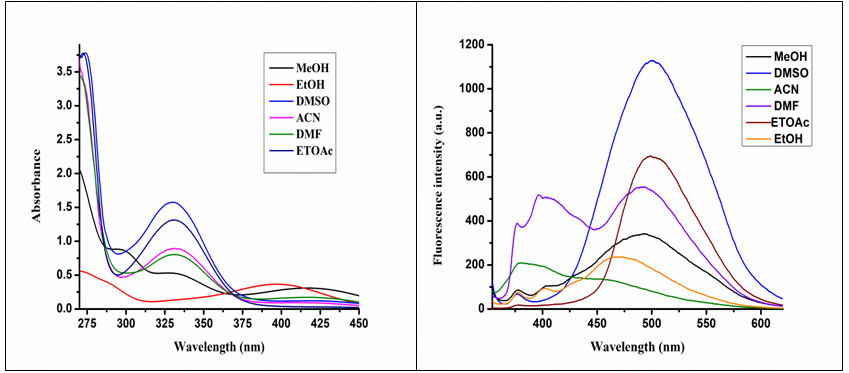


**Fig. S47** Effect of solvent on the absorption (left) and emission (right, λ_ex_ = 338 nm) spectra of L in presence of AsO_2_^-^





**Figure S48** Absorption spectroscopic monitoring of inter-conversion of iron red-ox states in the [L-Fe] complex.

**Table S1** Crystal parameters

| **Crystal parameters** | **L (CCDC NO. 1571936)** | **[L-Fe^2+^] (CCDC NO. 1576234)** | **[L-Fe^3+^] (CCDC NO. 1542015)** |
| --- | --- | --- | --- |
| Empirical formula | C_20_H_24_N_2_O_4_ | C_22_H_32_Fe N_10_ O_4_ | C_20_H_24_ClFeN_2_O_5_ |
| Formula weight | 356.41 g/mol | 525.35 g/mol | 463.71 g/mol |
| Temperature | 296(2) K | 296(2) K | 100 K |
| Wavelength | 0.71073 Å | 0.71073Å | 0.71073Å |
| Crystal system | triclinic | monoclinic | monoclinic |
| Space group | 'P -1' | 'P 21/c' | P 21/n |
| Hall group | '-P 1' | '-P 2ybc' | -P 2yn |
| Space group IT | 2 | 14 | 14 |
| Unit cell dimensions | a = 6.4949(9)Å; α= 90.930(9)°, b = 6.9114(8)Å; β=105.743(9)°, c = 10.7186(13)Å; γ = 97.123(10)° | a = 9.2248(3); α = 90°  b = 15.6509(5); β = 103.898(2)°  c = 16.8228(5);γ = 90° | a = 10.1000(5)Å ;α = 90°  b = 16.2227(8)Å ;β = 110.389(3)°  c = 13.6315(5)Å;γ = 90° |
| Volume | 458.90(10) | 2357.71 | 2093.58(17) |
| Z | 1 | 4 | 4 |
| Density (calculated) | 1.290 | 1.480 | 1.471 |
| Mu (mm-1) | 0.074 | 0.688 | 0.882 |
| F000 | 162.0 | 1208 | 964.0 |
| F000’ | 190.09 | 1097.74 | 966.35 |
| h, k,lmax | 8,9,14 | 10,18,19 | 13,21,18 |
| Tmin | 0.981 | 1.802 | 0.849,0.942 |
| Data completeness | 1.000 | 1.000 | 1.000 |
| Theta(max) | 29.733 | 24.696 | 28.282 |
| Reflections threshold_expression | I > 2\s(I) | I > 2\s(I) | I > 2\s(I) |
| Factor coef | Fsqd | Fsqd | Fsqd |
| Absorption coefficient | 0.082 mm^-1^ | 1.308 mm^-1^ | 0.092 mm^-1^ |

**Table S2** Selective bond angles and bond lengths of L

| L (CCDC NO. 1571936) | | | |
| --- | --- | --- | --- |
| ATOMS | ANGLES | ATOMS | LENGTHS |
| C004 - O001- H001 | 109.5 | O001 - C004 | 1.3450(18) |
| C005 - O002 - C008 | 118.07(13) | O001 - H001 | 0.8200 |
| C007- N003 - C00B | 119.51(15) | O002 - C005 | 1.3653(19) |
| O001 - C004 - C006 | 122.01(15) | O002 - C008 | 1.425(2) |
| O001 - C004 - C005 | 118.25(14) | N003 - C007 | 1.265(2) |
| C006 - C004 - C005 | 119.74(15) | N003 - C00B | 1.457(2) |
| O002 - C005 - C009 | 125.70(15) | C004 - C006 | 1.402(2) |
| O002 - C005 - C004 | 114.34(14) | C004 - C005 | 1.401(2) |
| C009 - C005 - C004 | 119.95(15) | C005 - C009 | 1.380(2) |
| C004 - C006 - C00A | 118.85(15) | C006 - C00A | 1.398(2) |
| C004 - C006 - C007. | 120.10(15) | C006 - C007 | 1.450(2) |
| C00A - C006 - C007 | 121.05(15) | C007 - H007 | 0.9300 |
| N003 - C007 - C006 | 122.68(15) | C008 - C00D | 1.497(3) |
| N003 - C007 - H007 | 118.7 | C008 - H00A | 0.9700 |
| C006 - C007 - H007 | 118.7 | C008 - H00B | 0.9700 |
| O002 - C008 - C00D | 106.73(14) | C009 - C00C | 1.394(3) |
| O002 - C008 - H00A | 110.4 | C009 - H009 | 0.9300 |
| C00D - C008 - H00A | 110.4 | C00A - C00C | 1.365(2) |
| C00D - C008 - H00B | 110.4 | C00B - C00B | 1.502(3) |
| H00A - C008 - H00B | 108.6 | C00B - H00D | 0.9700 |
| C005 - C009 - C00C | 120.26(16) | C00B - H00E | 0.9700 |
| C005 - C009 - H009 | 119.9 | C00C - H00F | 0.9300 |
| C00C - C009 - H009 | 119.9 | C00D - H00G | 0.9600 |
| C00C - C00A - C006 | 121.21(16) | C00D -H00H | 0.9600 |
| C00C - C00A - H00C | 119.4 | C00D - H00I | 0.9600 |
| H1W1 - O1W - H1W2 | 120.4 |  |  |
| C006 - C00A - H00C | 119.4 |  |  |
| N003 - C00B - C00B | 109.88(18) |  |  |
| N003 - C00B - H00D | 109.7 |  |  |
| C00B - C00B - H00D | 109.7 |  |  |
| N003 - C00B - H00E | 109.7 |  |  |
| C00B - C00B - H00E | 109.7 |  |  |
| C00A - C00C - C009 | 119.99(15) |  |  |

**Table S3** Selective bond angles and bond lengths of [L-Fe^2+^] complex

| **L-Fe^2+^ (CCDC NO. 1576234)** | | | |
| --- | --- | --- | --- |
| **ATOMS** | ANGLES | ATOMS | LENGTHS |
| O002-Fe01-O003 | 102.38(17) | Fe01-O002 | 1.922(4) |
| O002-Fe01-N00A | 92.2(2) | Fe01-O003 | 1.938(4) |
| O003-Fe01-N00A | 91.9(2) | Fe01-N00A | 2.061(6) |
| O002-Fe01-N009 | 167.3(2) | Fe01-N009 | 2.081(5) |
| O003-Fe01-N009 | 89.96(19) | Fe01-N008 | 2.103(5) |
| N00A-Fe01-N009 | 90.4(2) | Fe01-N00B | 2.132(6) |
| O002-Fe01-N008 | 89.7(2) | O002-C00D | 1.324(7) |
| O003-Fe01-N008 | 167.9(2) | O003-C00F | 1.329(7) |
| N00A-Fe01-N008 | 86.5(2) | O004-C00I | 1.372(7) |
| N009-Fe01-N008 | 78.0(2) | O004-C00J | 1.441(7) |
| O002-Fe01-N00B | 87.9(2) | O005-C00G | 1.369(7) |
| O003-Fe01-N00B | 93.6(2) | O005-C00L | 1.434(7) |
| N00A-Fe01-N00B | 174.3(2) | N1-N00C | 1.151(7) |
| N009-Fe01-N00B | 88.4(2) | N1-N00B | 1.187(7) |
| N008-Fe01-N00B | 87.8(2) | N007-C00M | 1.499(8) |
| C00D-O002-Fe01 | 130.9(4) | N007-H00A | 0.8900 |
| C00F-O003-Fe01 | 129.0(4) | N008-C00K | 1.265(8) |
| C00I-O004-C00J | 117.4(5) | N008-C00R | 1.476(8) |
| C00G-O005-C00L | 117.8(5) | N009-C00O | 1.274(8) |
| N00C-N1-N00B | 176.2(7) | N009-C00P | 1.482(8) |
| C00M-N007-H00A | 109.5 | N00A-N2 | 1.168(8) |
| C00M-N007-H00B | 109.5 | C00D-C00G | 1.402(8) |
| H00A-N007-H00B | 109.5 | C00D-C00H | 1.418(8) |
| C00M-N007-H111 | 109.5 | N2-N3 | 1.151(9) |
| H00A-N007-H111 | 109.5 | C00F-C00I | 1.402(9) |
| H00B-N007-H111 | 109.5 | C00F-C00Q | 1.422(9) |
| C00K-N008-C00R | 119.8(6) | C00G-C00T | 1.393(9) |
| C00K-N008-Fe01 | 125.0(5) | C00H-C00X | 1.418(9) |
| C00R-N008-Fe01 | 115.0(4) | C00H-C00K | 1.439(9) |
| C00O-N009-C00P | 121.5(6) | C00I-C00W | 1.386(9) |
| C00O-N009-Fe01 | 124.0(5) | C00J-C00V | 1.495(9) |
| C00P-N009-Fe01 | 114.3(4) | C00J-H112 | 0.9700 |
| N2-N00A-Fe01 | 128.7(5) | C00J-H00D | 0.9700 |
| N1-N00B-Fe01 | 133.0(5) | C00K-H00K | 0.9300 |
| O002-C00D-C00G | 117.9(5) | C00L-C00N | 1.478(9) |
| O002-C00D-C00H | 123.6(6) | C00L-H00E | 0.9700 |
| C00G-C00D-C00H | 118.4(6) | C00L-H00F | 0.9700 |
| N3-N2-N00A | 177.3(10) | C00M-H00G. | 0.9700 |
| O003-C00F-C00I | 117.9(6) | C00M-H00H | 0.9700 |
| O003-C00F-C00Q | 123.1(6) | C00N-H00I | 0.9600 |
| C00I-C00F-C00Q | 118.9(6) | C00N-H00J | 0.9600 |
| N3-N2-N00A | 177.3(10) | C00N-H00L | 0.9600 |
| O003-C00F-C00I | 117.9(6) | C00O-C00Q | 1.441(9) |
| O005-C00G-C00T | 123.9(6) | C00O-H00O | 0.9300 |
| O005-C00G-C00D | 115.2(5) | C00P-C00R | 1.475(9) |
| C00T-C00G-C00D | 120.9(6) | C00R-H00P | 0.9700 |
| C00D-C00H-C00X | 118.1(7) | C00R-H00Q | 0.9700 |
| C00D-C00H-C00K | 124.0(6) | C00S-C00U | 1.347(10) |
| O004-C00I-C00W | 123.2(6) | C00S-H00S | 0.9300 |
| O004-C00I-C00F | 116.1(5) | C00T-C00Y | 1.383(10) |
| C00W-C00I-C00F | 120.7(6) | C00T-H00T | 0.9300 |
| O004-C00J-C00V | 109.3(6) | C00U-H00U | 0.9300 |
| O004-C00J-H112 | 109.8 | C00V-H00R | 0.9600 |
| C00V-C00J-H112 | 109.8 | C00V-H00V | 0.9600 |
| O004-C00J-H00D | 109.8 | C00V-H00W | 0.9600 |
| C00V-C00J-H00D | 109.8 | C00W-H00$ | 0.9300 |
| N008-C00K-C00H | 126.7(6) | C00X-C00Y | 1.337(10) |
| N008-C00K-H00K | 116.7 | C00X-H00X | 0.9300 |

**Table S4** Selective bond angles and bond lengths of [L-Fe^3+^] complex

| L-Fe^3+^(CCDC NO. 1542015) | | | |
| --- | --- | --- | --- |
| ATOMS | ANGLE | ATOMS | LENGTH |
| O2 - Fe1 - O1 | 101.23(6) | Fe1 - O2 | 1.8993(14) |
| O2 - Fe1 - N2 | 89.29(6) | Fe1 - O1 | 1.9006(13) |
| O1 - Fe1 - N2 | 166.40(6) | Fe1 - N2 | 2.0875(16) |
| O2 -Fe1 -N1 | 164.32(6) | Fe1 - N1 | 2.0897(16) |
| O1 - Fe1 - N1 | 88.62(6) | Fe - O1W | 2.2145(15) |
| N2 - Fe1 - N1 | 79.35(6) | Fe1 - Cl1 | 2.3454(6) |
| O2 - Fe1 - O1W | 87.88(6) | N1 - C7 | 1.285(2) |
| O1 - Fe1 - O1W | 87.58(6) | N1 - C8 | 1.471(2) |
| N2 - Fe1 - O1W | 84.18(6) | N2 - C10 | 1.282(3) |
| N1 - Fe1 - O1W | 80.31(6) | N2 - C9 | 1.465(3) |
| O2 - Fe1 - Cl1 | 99.86(5) | O1 - C1 | 1.316(2) |
| O1 - Fe1 - Cl1 | 97.19(4) | O2 - C16 | 1.317(2) |
| N2 - Fe1- Cl1 | 89.38(5) | O1W - H1W | 1 0.79(3) |
| N1- Fe1 - Cl1 | 90.86(5) | O1W - H1W | 2 0.79(3) |
| O1W - Fe1 - Cl1 | 169.89(4) | C1 - C6 | 1.410(3) |
| C7 - N1 - C8 | 121.19(17) | C1 - C2 | 1.424(3) |
| C7 - N1 - Fe1 | 124.71(14) | C2 - O3 | 1.361(2) |
| C8 - N1 - Fe1 | 114.01(12) | C2 - C3 | 1.376(3) |
| C10 - N2 - C9 | 121.32(17) | C3 - C4 | 1.398(3) |
| C10 - N2- Fe1 | 125.73(14) | C3 - H3 | 0.9500 |
| C9 - N2 - Fe1 | 112.92(12) | C4 - C5 | 1.370(3) |
| C1 - O1 - Fe1 | 128.56(12) | C4 - H4 | 0.9500 |
| C16 - O2 - Fe1 | 131.86(13) | C5 - C6 | 1.414(3) |
| Fe1 - O1W - H1W1 | 114(2) | C5 - H5 | 0.9500 |
| Fe1 - O1W - H1W2 | 108(2) | C6- C7 | 1.451(3) |
| H1W1 - O1W - H1W2 | 103(3) | C7 - H7 | 0.9500 |
| O1 - C1 - C6 | 123.83(17) | C8 - C9 | 1.526(3) |
| O1 - C1- C2 | 117.21(17) | C8 - H8A | 0.9900 |
| C6 - C1- C2 | 118.95(17) | C8 - H8B | 0.9900 |
| O3 - C2 - C3 | 125.97(18) | C9 - H9A | 0.9900 |
| O3 - C2 - C1 | 113.22(16) | C9 - H9B | 0.9900 |
| C3 - C2 - C1 | 120.81(18) | C10 - C11 | 1.448(3) |
| C2 - C3 - C4 | 119.60(19) | C10- H10 | 0.9500 |
| C2 - C3 - H3 | 120.2 | C11 - C12 | 1.412(3) |
| C4 - C3 - H3 | 120.2 | C11- C16 | 1.417(3) |
| C5 - C4 - C3 | 120.97(19) | C12 - C13 | 1.368(3) |
| C5 - C4 - H4 | 119.5 | C12 - H12 | 0.9500 |
| C3 - C4 - H4 | 119.5 | C13 - C14 | 1.396(3) |
| C4 - C5 - C6 | 120.7(2) | C13 - H13 | 0.9500 |
| C4 - C5 - H5 | 119.6 | C14 - C15 | 1.383(3) |
| C6 - C5 - H5 | 119.6 | C14 - H14 | 0.9500 |
| C1 - C6 - C5 | 118.91(18) | C15 - O4 | 1.368(2) |
| C1 - C6 - C7 | 123.45(18) | C15 - C16 | 1.416(3) |
| C5 - C6 - C7 | 117.55(18) | O3 - C17 | 1.437(2) |
| N1- C7 - C6 | 124.36(19) | C17 - C18 | 1.501(3) |
| N1 - C7 - H7 | 117.8 | C17 - H17A | 0.9900 |
| C6 - C7 - H7 | 117.8 | C17 - H17B | 0.9900 |
| N1 - C8 - C9 | 107.72(16) | O4 - C19 | 1.429(2) |
| N1 - C8 - H8A | 110.2 | C18 - H18A | 0.9800 |
| C9 - C8 - H8A | 110.2 | C18 - H18B | 0.9800 |
| N1 - C8 - H8B | 110.2 | C18 - H18C | 0.9800 |
| C9 - C8 - H8B | 110.2 | C19 - C20 | 1.498(3) |
| H8A - C8 - H8B | 108.5 | C19 - H19A | 0.9900 |
| N2 - C9 - C8 | 108.58(16) | C19 - H19B | 0.9900 |
| N2 - C9 - H9A | 110.0 | C20 - H20A | 0.9800 |
| C8 - C9- H9A | 110.0 | C20 - H20B | 0.9800 |
| N2 - C9 - H9B | 110.0 | C20 - H20C | 0.9800 |
| C8 - C9 - H9B | 110.0 |  |  |
| H9A - C9 - H9B | 108.4 |  |  |
| N2 - C10 - C11 | 125.94(18) |  |  |
| N2 - C10 - H10 | 117.0 |  |  |
| C11 - C10 - H10 | 117.0 |  |  |
| C12 - C11 - C16 | 119.29(19) |  |  |
| C12 - C11 - C10 | 117.96(18) |  |  |
| C16 - C11- C10 | 122.74(18) |  |  |
| C13 - C12 - C11 | 121.09(19) |  |  |
| C13 - C12 - H12 | 119.5 |  |  |
| C11 - C12 - H12 | 119.5 |  |  |
| C12 - C13 - C14 | 120.33(19) |  |  |
| C12 - C13 - H13 | 119.8 |  |  |
| C14 - C13 - H13 | 119.8 |  |  |
| C15 - C14 - C13 | 119.91(19) |  |  |
| C15 - C14 - H14 | 120.0 |  |  |
| C13 - C14 - H14 | 120.0 |  |  |
| O4 - C15 - C14 | 125.46(18) |  |  |
| O4 - C15 - C16 | 113.39(16) |  |  |
| C14 - C15 - C16 | 121.15(18) |  |  |
| C2 - O3 - C17 | 118.21(15) |  |  |
| O2 - C16 - C15 | 117.69(17) |  |  |
| O2 - C16 - C11 | 124.12(18) |  |  |
| C15 - C16 - C11 | 118.17(17) |  |  |
| O3 - C17 - C18 | 106.90(16) |  |  |
| O3 - C17 - H17A | 110.3 |  |  |
| C18 - C17 - H17A | 110.3 |  |  |
| O3 - C17 - H17B | 110.3 |  |  |
| C18 - C17 - H17B | 110.3 |  |  |
| H17A - C17 - H17B | 108.6 |  |  |
| C15 - O4 - C19 | 118.30(15) |  |  |
| C17 - C18 - H18A | 109.5 |  |  |
| C17 - C18 - H18B | 109.5 |  |  |
| H18A - C18 - H18B | 109.5 |  |  |
| C17 - C18 - H18C | 109.5 |  |  |
| H18A - C18 - H18C | 109.5 |  |  |
| H18B - C18 -H18C | 109.5 |  |  |
| O4 - C19- C20 | 107.45(16) |  |  |
| O4 - C19 - H19A | 110.2 |  |  |
| C20 - C19 - H19A | 110.2 |  |  |
| O4 - C19 - H19B | 110.2 |  |  |
| C20 - C19 - H19B | 110.2 |  |  |
| H19A - C19 - H19B | 108.5 |  |  |
| C19 - C20 - H20A | 109.5 |  |  |
| C19 - C20 - H20B | 109.5 |  |  |
| H20A - C20 - H20B | 109.5 |  |  |
| C19 - C20 - H20C | 109.5 |  |  |
| H20A - C20 - H20C | 109.5 |  |  |
| H20B - C20 - H20C | 109.5 |  |  |

**Table S5** Comparison with reported Fe^2+^ and Fe^3+^ selective probes

| **Probe type** | **Medium** | **Mechanism** | **LOD** | **Ref.** | **Limitation** |
| --- | --- | --- | --- | --- | --- |
| 1,3-Bis-calix [4] arene  conjugate-based | CH_3_CN | LMCT | 0.334 ± 0.020 μM(Fe^3+^) | *Anal. Chem.*,**2013***, 85*, 3707−3714 | 1. Unable to proper discrimination between Fe^2+^ and Fe^3+^  2. X-ray structural evidence of iron binding absent  3. Unable to detect at nano-molar level |
| Schiff base (Thiophene based) | Acetonitrile/ water (1/4, v/v) | CHEF | 2.0 μM (Fe^2+^) and 3.5 μM (Fe^3+^) | *Analyst,* **2012***, 137*, 3335–3342 | 1. Unable to proper discrimination between Fe^2+^ and Fe^3+^  2. X-ray structural evidence of iron binding absent  3. Unable to detect at nano-molar level |
| Anthracene-Based Probe (Salen-type) | DMF | LMCT | 0.44 μM (Fe^3+^) and 0.69 μM (Fe^2+^) | *Inorg. Chem.*, **2019**, *58*, 20, 13796–13806 | 1. Unable to proper discrimination between Fe^2+^ and Fe^3+^  2. X-ray structural evidence of iron binding absent  3. Unable to detect at nano-molar level |
| Fluorescent Schiff base probe | DMF-deionized water solution (1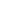: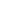1, v/v) | CHEQ | 2.17 x 10^-6^ M for Fe^3+^, 2.06 x 10^-6^ M for Fe^2+^ | [*Anal. Methods*](https://doi.org/10.1039/1759-9679/2009), **2019**, *11*, 642-647 | 1. Unable to proper discrimination between Fe^2+^ and Fe^3+^  2. X-ray structural evidence of iron binding absent  3. Unable to detect at nano-molar level |
| Schiff base | Methanol | Electron transfer as Fe(III) is a strong Lewis acid | 20.85 μM for Fe^3+^ | *RSC Adv****.***, **2014**, *4*, 48516-48521 | 1. Unable to proper discrimination between Fe^2+^ and Fe^3+^  2. X-ray structural evidence of iron binding absent  3. Unable to detect at nano-molar level |
| Benzothiazole conjugated quinoline derivative appended with rhodamine-6G | MeOH/H_2_O (2/3, v/v) | FRET | 5.39 x 10^-8^ M for Fe^3+^ | *New J. Chem.*, **2016**, *40*, 6414-6420 | 1. Unable to proper discrimination between Fe^2+^ and Fe^3+^  2. X-ray structural evidence of iron binding absent |
| Schiff base (Juloidine-imidazole based) | DMF | MLCT | Fe^2+^ (7.4 μM) and Fe^3+^ (6.8 μM) | [*Sensors and Actuators B: Chemical*](https://www.sciencedirect.com/journal/sensors-and-actuators-b-chemical), **2014**, *194*, 343-352 | 1. Unable to proper discrimination between Fe^2+^ and Fe^3+^  2. X-ray structural evidence of iron binding absent  3. Unable to detect at nano-molar level |
| Schiff base (Chitosan, PVA and 9anthracenealdehyde based fluorescent hydrogel) | Aqueous | CHEF for Fe^3+^ and CHEQ for Fe^2+^ | Fe^2+^ (0.138 nM) and Fe^3+^ (0.124 nM) | [*Carbohydrate Polymers*](https://www.sciencedirect.com/journal/carbohydrate-polymers), **2018**, *193*, 119-128 | 1. Unable to proper discrimination between Fe^2+^ and Fe^3+^  2. X-ray structural evidence of iron binding absent |
| Polyvinylpyrrolidone stabilized copper nanocluster (CuNCs@PVP) | NaOAc-HOAc buffer (10 mM, pH = 3.6) | Static quenching | Fe^2+^ (0.008 μM) and Fe^3+^ (0.14 μM) | [*Analytical Biochemistry*](https://www.sciencedirect.com/journal/analytical-biochemistry), **2021**, *623*, 114171 | 1. Unable to proper discrimination between Fe^2+^ and Fe^3+^  2. X-ray structural evidence of iron binding absent  3. Unable to detect at nano-molar level |
| SME capped AgNps (SME=Sapindusmukorossiextract) | Aqueous | LMCT | Fe^2+^ (1 μM) and Fe^3+^ (5 μM) | *New J. Chem.*, **2021**, *45*, 9936-9943 | 1. Unable to proper discrimination between Fe^2+^ and Fe^3+^  2. X-ray structural evidence of iron binding absent  3. Unable to detect at nano-molar level |
| Morpholine based  Rhodamine B derivatives | CH_3_CN:H_2_O (9:1, v/v) | Transfer of  resonance energy from the morpholine-type moiety to the RhB framework | - | [*Sensors and Actuators B: Chemical*](https://www.sciencedirect.com/journal/sensors-and-actuators-b-chemical),**2017**, *248*, 646-656 | 1. Unable to proper discrimination between Fe^2+^ and Fe^3+^  2. X-ray structural evidence of iron binding absent |
| Ferrocene based  receptors | CH_3_CN/H_2_O (2/8, v/v) | LMCT | Fe^2+^ (60 nM) and Fe^3+^ (45 nM) | *Organometallics***, 2017,** *36*, 11, 2141–2152 | 1. Unable to proper discrimination between Fe^2+^ and Fe^3+^  2. X-ray structural evidence of iron binding absent |
| Pyridyl /phenolic/  benzothiazole functionalized chemosensor | Ethanol/water | Ligand based transitions after coordination | Fe^2+^ (2.5 μM) and Fe^3+^ (5.5 μM) | [*Dyes and Pigments*](https://www.sciencedirect.com/journal/dyes-and-pigments), **2018**, *155*,249-257 | 1. Unable to proper discrimination between Fe^2+^ and Fe^3+^  2. X-ray structural evidence of iron binding absent  3. Unable to detect at nano-molar level |
| Pyrimidine based Schiff-base | Acetonitrile (ACN) -aqueous buffer (4:1) | Static or purely dynamic quenching  due to the formation of a  ground state non fluorescent complex | Fe^2+^ (1.57 × 10^-5^ M) and Fe^3+^ (1.28 × 10^-5^ M) | *Journal of Molecular Structure*, **2019**,*1184*,102-109 | 1. Unable to proper discrimination between Fe^2+^ and Fe^3+^  2. X-ray structural evidence of iron binding absent  3. Unable to detect at nano-molar level |
| Schiff base (Salicyaldehyde based) | MeOH/ H_2_O (4/1, v/v) HEPES buffer (10 mM) | CHEQ | 0.008 µM (Fe^2+^) and 0.005 µM (Fe^3+^) | **Present work** | 1. Capable to discriminate between Fe^2+^ and Fe^3+^  2. X-ray structural evidence of iron binding is present  3. Capable to detect at nano-molar level |

**Table S6** Theoretical TDDFT results of L

| **Compound** | **Electronic** **Transitions** | **Energy^a^(eV)** | **Wavelength**  **(nm)** | **f^b^** | **Transitions**  **involved** |
| --- | --- | --- | --- | --- | --- |
| L | S_0_→S_1_  S_0_→S_2_  S_0_→S_3_  S_0_→S_4_  S_0_→S_5_  S_0_→S_6_ | 2.2852 eV  2.4550 eV  2.6976 eV  2.9634 eV  3.0825 eV  3.1846 eV | 542.56 nm  505.02 nm  459.61 nm  418.38 nm  402.22 nm  389.33 nm | 0.0020  0.0073  0.0194  0.0174  0.0090  0.0139 | HOMO-2→LUMO  HOMO-2→LUMO+1  HOMO-2→LUMO  HOMO-2→LUMO+1  HOMO-4→LUMO+1  HOMO→LUMO+1  HOMO→LUMO  HOMO-2→LUMO  HOMO-5→LUMO  HOMO-1→LUMO  HOMO-1→LUMO+1  HOMO→LUMO  HOMO-5→LUMO  HOMO-3→LUMO  HOMO-2→LUMO  HOMO-2→LUMO+1  HOMO-1→LUMO  HOMO→LUMO |

**Table S7** Theoretical TDDFT results of [L-Fe^2+^] complex

| **Compound** | **Electronic** **Transitions** | **Energy^a^(eV)** | **Wavelength**  **(nm)** | **f^b^** | **Transitions**  **involved** |
| --- | --- | --- | --- | --- | --- |
| **[**L-Fe^2+^**]** | S_0_→S_2_  S_0_→S_2_  S_0_→S_3_  S_0_→S_4_  S_0_→S_5_  S_0_→S_6_ | 0.1568 eV  0.5510 eV  0.6614 eV  0.8717 eV  1.0766 eV  1.2713 eV | 1909.38 nm  1450.10 nm  1374.70 nm  1022.29 nm  751.60 nm  645.23 nm | 0.0001  0.0023  0.0010  0.0019  0.0088  0.0256 | HOMO-2→LUMO  HOMO-1→LUMO  HOMO→LUMO  HOMO-4→LUMO  HOMO-3→LUMO-1  HOMO-3→LUMO  HOMO-2→LUMO  HOMO-2→LUMO+1  HOMO-1→LUMO+1  HOMO→LUMO+1  HOMO-3→LUMO  HOMO-3→LUMO+1  HOMO-2→LUMO  HOMO→LUMO  HOMO-1→LUMO+2  HOMO→LUMO+2  HOMO-4→LUMO  HOMO-3→LUMO  HOMO-3→LUMO+1  HOMO-2→LUMO  HOMO-2→LUMO+1  HOMO-1→LUMO  HOMO-1→LUMO+1  HOMO-3→LUMO  HOMO-3→LUMO+1  HOMO-2→LUMO  HOMO-2→LUMO+1  HOMO-1→LUMO  HOMO-1→LUMO+1  HOMO-7→LUMO  HOMO-7→LUMO+1  HOMO-6→LUMO  HOMO-4→LUMO  HOMO-4→LUMO+1  HOMO-2→LUMO+1 |

**Table S8** Data from theoretical TDDFT studies of [L-Fe^3+^] complex

| Compound | Electronic Transitions | Energy^a^(eV) | Wavelength (nm) | f^b^ | Transitions  involved |
| --- | --- | --- | --- | --- | --- |
| [L-Fe^3+^] | S_0_→S_1_  S_0_→S_2_  S_0_→S_3_  S_0_→S_4_  S_0_→S_5_  S_0_→S_6_ | 0.6505 eV  0.7748 eV  1.8137 eV  1.8516 eV  2.0495 eV  2.0774 eV | 1600.29 nm  683.61 nm  669.62 nm  651.62 nm  604.94 nm  596.81 nm | 0.0002  0.0003  0.0071  0.0012  0.0035  0.0019 | HOMO-1→LUMO+1  HOMO-1→LUMO  HOMO-3→LUMO-1  HOMO-4→LUMO+3  HOMO-4→LUMO+1  HOMO-4→LUMO-1  HOMO-5→LUMO+1  HOMO-1→LUMO  HOMO-1→LUMO-1  HOMO-1→LUMO  HOMO-3→LUMO+5  HOMO-3→LUMO+4  HOMO-3→LUMO+2  HOMO-3→LUMO+1  HOMO→LUMO  HOMO-1→LUMO+5  HOMO-1→LUMO+4  HOMO-1→LUMO+2  HOMO-1→LUMO  HOMO-1→LUMO-1  HOMO-3→LUMO+5  HOMO-3→LUMO+3  HOMO-3→LUMO+1  HOMO-3→LUMO  HOMO→LUMO+1  HOMO→LUMO  HOMO-1→LUMO  HOMO-1→LUMO-1  HOMO-2→LUMO  HOMO-3→LUMO+5  HOMO-6→LUMO+3  HOMO-3→LUMO+5  HOMO-3→LUMO  HOMO-4→LUMO+5  HOMO-4→LUMO+4  HOMO-4→LUMO+2  HOMO-5→LUMO+5  HOMO→LUMO  HOMO-1→LUMO  HOMO-1→LUMO+2  HOMO-1→LUMO+3  HOMO-1→LUMO  HOMO-2→LUMO  HOMO-2→LUMO-1  HOMO-3→LUMO+5  HOMO-6→LUMO+5  HOMO-3→LUMO+5  HOMO-3→LUMO+2  HOMO-4→LUMO+5  HOMO-4→LUMO+4  HOMO-4→LUMO+2  HOMO-5→LUMO+5  HOMO→LUMO  HOMO-1→LUMO |

**Table S9** Comparison with available AsO_2_^-^ selective probes

| **Probe type** | **Media** | **LOD** | **Ref.** |
| --- | --- | --- | --- |
| Schiff-base (DFP based) | DMSO/ H_2_O (1/9,  v/v) HEPES buffer (1 mM) at 25°C | 54.91x10^-9^ M | *Anal. Chem.,* **2014**, 86, 11357–11361 |
| Schiff-base (naphthalene based) | DMF/H_2_O (9/1, v/v) HEPES buffer, pH 7.2. | 66 nM | *RSC Adv.,* **2016**, 6, 100136-100144 |
| Schiff-base (salicyaldehyde based), **H- bond assisted CHEF process** | DMSO/ H_2_O (4/1,  v/v) HEPES buffer (10 mM) at 25°C | **2x10^−12^M** | **Present work** |

**Table S10** Fluorescence life time data of L and its AsO_2_^-^ adduct

| Life time data | B_1_ | τ_1_/ns | B_1_ | τ_2_/ns | ˂τ˃/ns |
| --- | --- | --- | --- | --- | --- |
| **L** at 384 nm | 0.3171 | 0.105 | 0.116 | 2.011 | 0.2664 |
| [**L**-AsO_2_^-^] at 450 nm | 0.1956 | 0.311 | 0.8236 | 1.287 | 1.1208 |

**Table S11** Theoretical TDDFT results on [L-AsO_2_^-^] adduct

| **Compound** | **Electronic** **transitions** | **Energy^a^ (eV)** | **Wavelength**  **(nm)** | **f^b^** | **Transitions**  **involved** |
| --- | --- | --- | --- | --- | --- |
| [**L-**AsO_2_^-^] | S_0_→S_1_  S_0_→S_2_  S_0_→S_3_  S_0_→S_4_  S_0_→S_5_  S_0_→S_6_ | 0.3266 eV  0.3999 eV  0.4740 eV  0.8895 eV  1.1565 eV  1.5027 eV | 1703.35 nm  1405.22 nm  1319.19 nm  1277.48 nm  1072.04 nm  825.05 nm | 0.0077  0.0194  0.0011  0.0189  0.0038  0.0108 | HOMO-5→LUMO-1  HOMO-3→LUMO-1  HOMO-2→LUMO-1  HOMO-1→LUMO-1  HOMO-5→LUMO-1  HOMO-4→LUMO-1  HOMO-2→LUMO-1  HOMO-1→LUMO-1  HOMO-7→LUMO-1  HOMO-3→LUMO-1  HOMO-2→LUMO-1  HOMO-3→LUMO  HOMO-3→LUMO-1  HOMO-4→LUMO-1  HOMO-4→LUMO  HOMO-2→LUMO-1  HOMO-1→LUMO-1  HOMO-1→LUMO  HOMO-3→LUMO  HOMO-2→LUMO  HOMO-1→LUMO-1  HOMO-3→LUMO  HOMO-3→LUMO+1  HOMO-2→LUMO  HOMO-2→LUMO+1  HOMO-1→LUMO  HOMO→LUMO  HOMO→LUMO+1  HOMO-1→LUMO+1 |

**Table S12** Comparison as analytical reagent for identification and determination Fe^2+^ and Fe^3+^ in a mixture

| **Serial no.** | **Test and regents** | **Fe^2+^** | **Fe^3+^** | **mixture of Fe^2+^ and Fe^3+^** | **Identification** | **Remarks** |
| --- | --- | --- | --- | --- | --- | --- |
| 1 | NH_4_OH | no | yes | yes | precipitation | Al^3+^, Cr^3+^, Co^2+^, Ni^2+^, Zn^2+^ and Mg^2+^ interfere |
| 2 | H_2_S and sodium acetate | yes | no | no | black precipitation | Insufficient solubility of FeS |
| 3 | Potassium cyanide | yes | no | no | yellow-brown | Decomposition of the complex results toxic CO. |
| 4 | NH_4_SCN | no | yes | yes | red | Cu^2+^, Ag^+^, Zn^2+^, Pb^2+^ and Hg^2+^ interferes |
| 5 | 2,2’-Bipyridyl | yes | no | no | red | halides, sulphate and Fe^3+^ interferes |
| 6 | Dimethylglyoxime (DMG) | yes | no | no | red | Ni^2+^, Co^2+^ and Cu^2+^ interferes |
| 7 | 1,10-phenanthroline | yes | no | no | red | Aerial oxidation of Fe^2+^ reduces efficiency, hydroxylamine hydrochloride is used |
| 8 | Cupferron | no | yes | yes | Red-brown | Toxic, less soluble, decomposition in alkali media |
| 9 | Ferron reagent | no | yes | no | Green- blue | Cu^2+^ interferes |
| 10 | Potassium ferricyanide | no | yes | - | Prussian blue | Fe^2+^, decomposes in conc. HCl |
| 11 | Potassium ferrocyanide | yes | no | - | Turnbull blue | Fe^3+^, aerial oxidation of Fe^2+^ |
| 12 | **PRESENT PROBE** | Yes (538 nm | Yes (606 nm) | Yes (538 nm) | Intense blood red for Fe^2+^ and intense violet for Fe^3+^ | No interference, L is very stable, synthesis is very simple and inexpensive. |

**Reference**

1. Ghosh, M., Ghosh, A., Ta, S., Matalobos, J. S. & Das, D. ESIPT-Based Nanomolar Zn^2+^ Sensor for Human Breast Cancer Cell (MCF7) Imaging. *ChemistrySelect***2**, 7426–7431 (2017).
2. Skoog, D. A., West, D. M., Holler, F. J. & Crouch, S. R. *Fundamentals of Analytical Chemistry*. (Cengage Learning, 2013).
3. Welcher, F. J. A text-book of quantitative inorganic analysis including elementary instrumental analysis (Vogel, Arthur I.). *J. Chem. Educ.***40**, A68 (1963).
